# Supplementary material for: Prevalence patterns of overweight and obesity in the world: An age-period-cohort analysis
Source: PLoS One. 2025 Jun 18;20(6):e0324733. doi: 10.1371/journal.pone.0324733 (PMC12176128; doi:10.1371/journal.pone.0324733)
Supplement: S1 File — This file contains plots of trends of obesity and overweight alongside the corresponding APC analysis on the study subgroups. The other file, titled gather-checklist.docx, contains details and points that should be explained based on the study design. Accordingly, the methodological issues and details that should be observed are addressed in this file. (DOCX) [file pone.0324733.s001.docx]

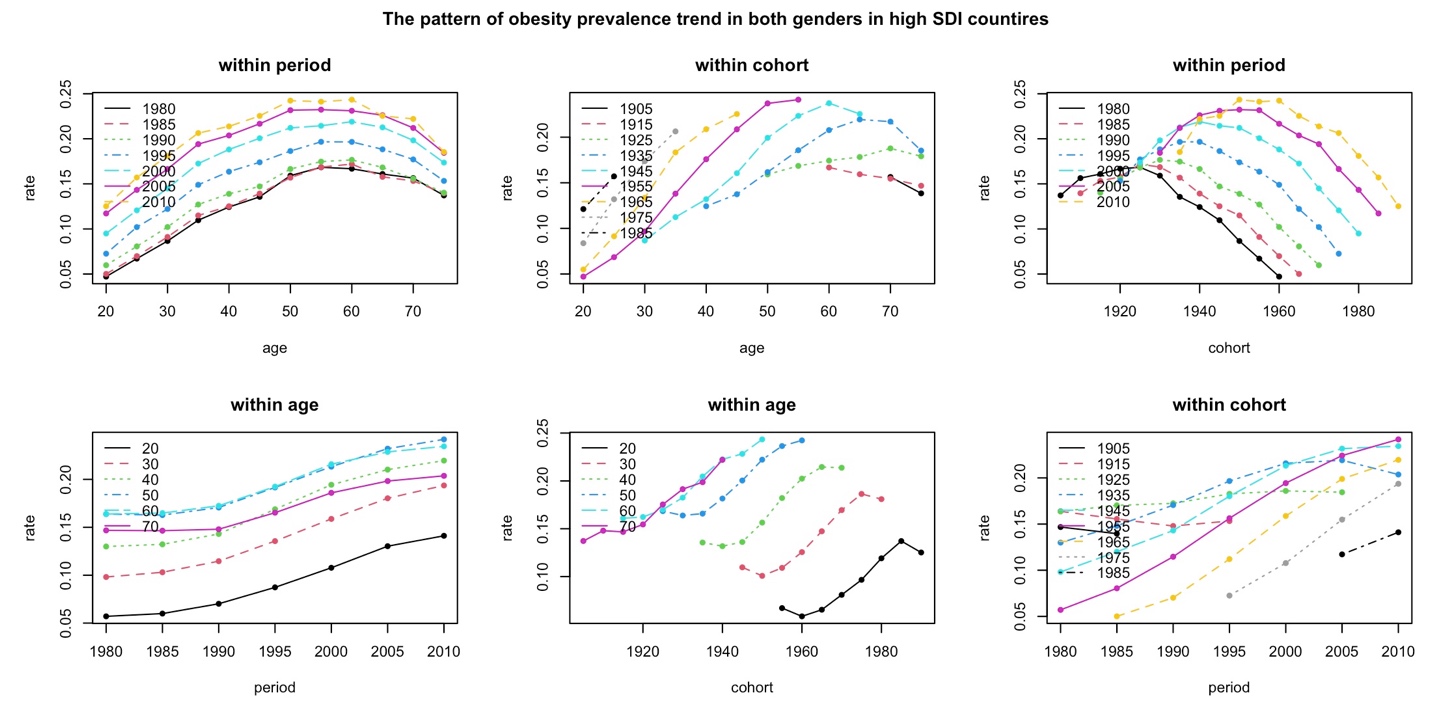


Supplementary Supplementary Figure 1. The trend of obesity prevalence rate within period-age, cohort-age, and period-cohort for both genders in High SDI countries


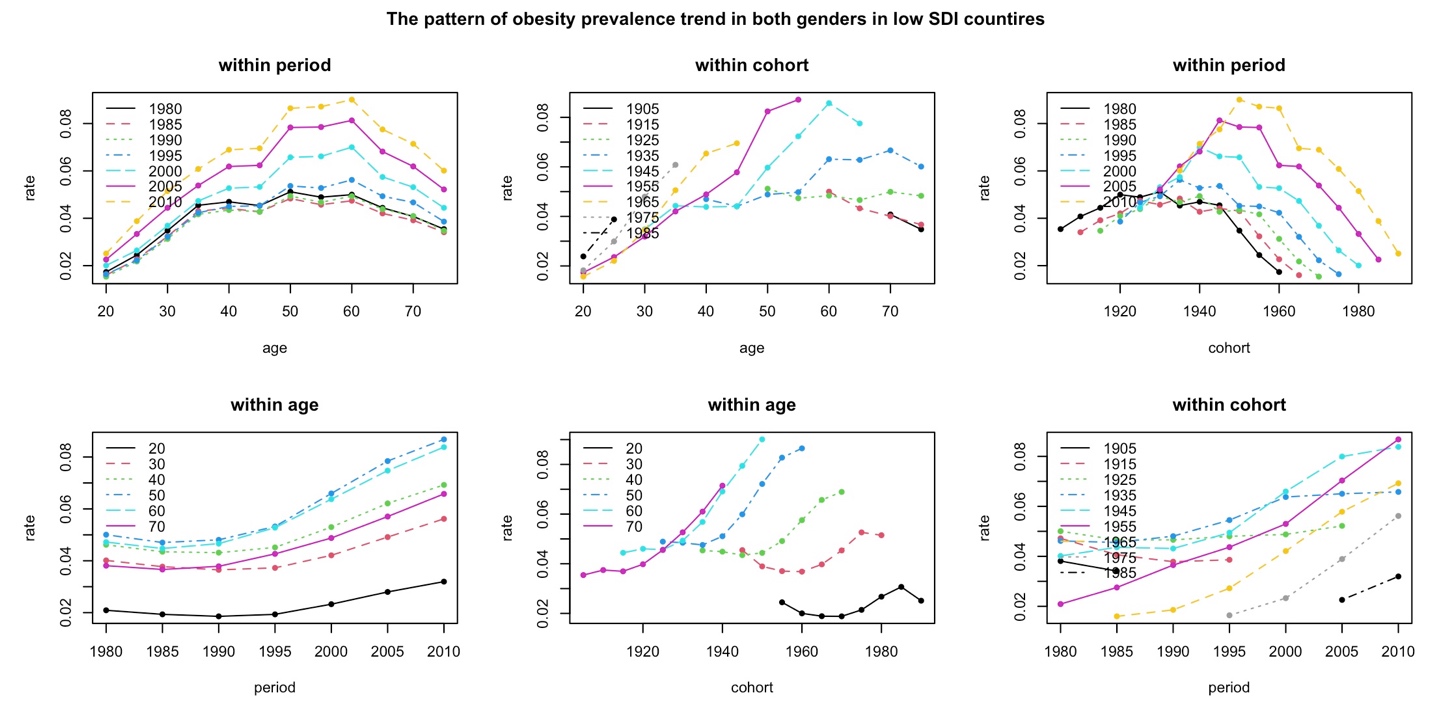


Supplementary Supplementary Figure 2. The trend of obesity prevalence rate within period-age, cohort-age, and period-cohort for both genders in Low SDI countries


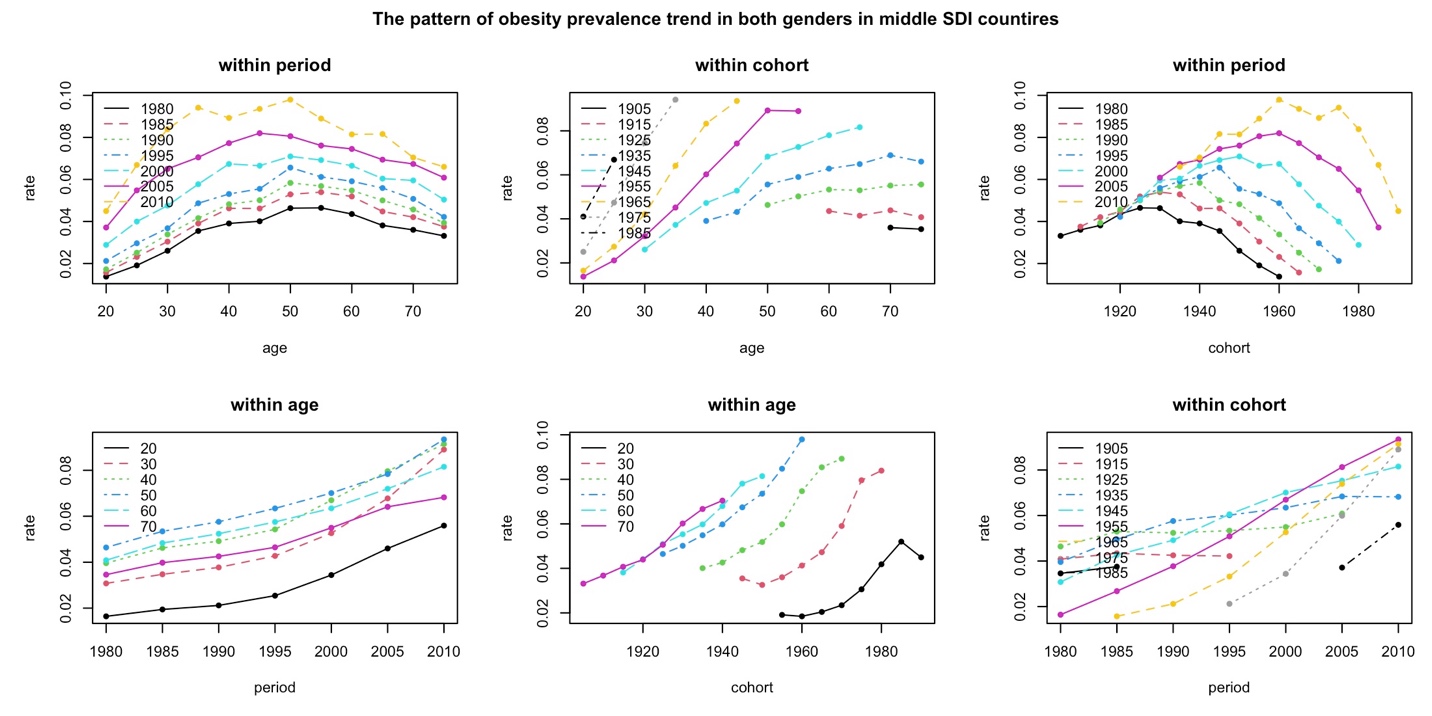


Supplementary Supplementary Figure 3. The trend of obesity prevalence rate within period-age, cohort-age, and period-cohort for both genders in Middle SDI countries


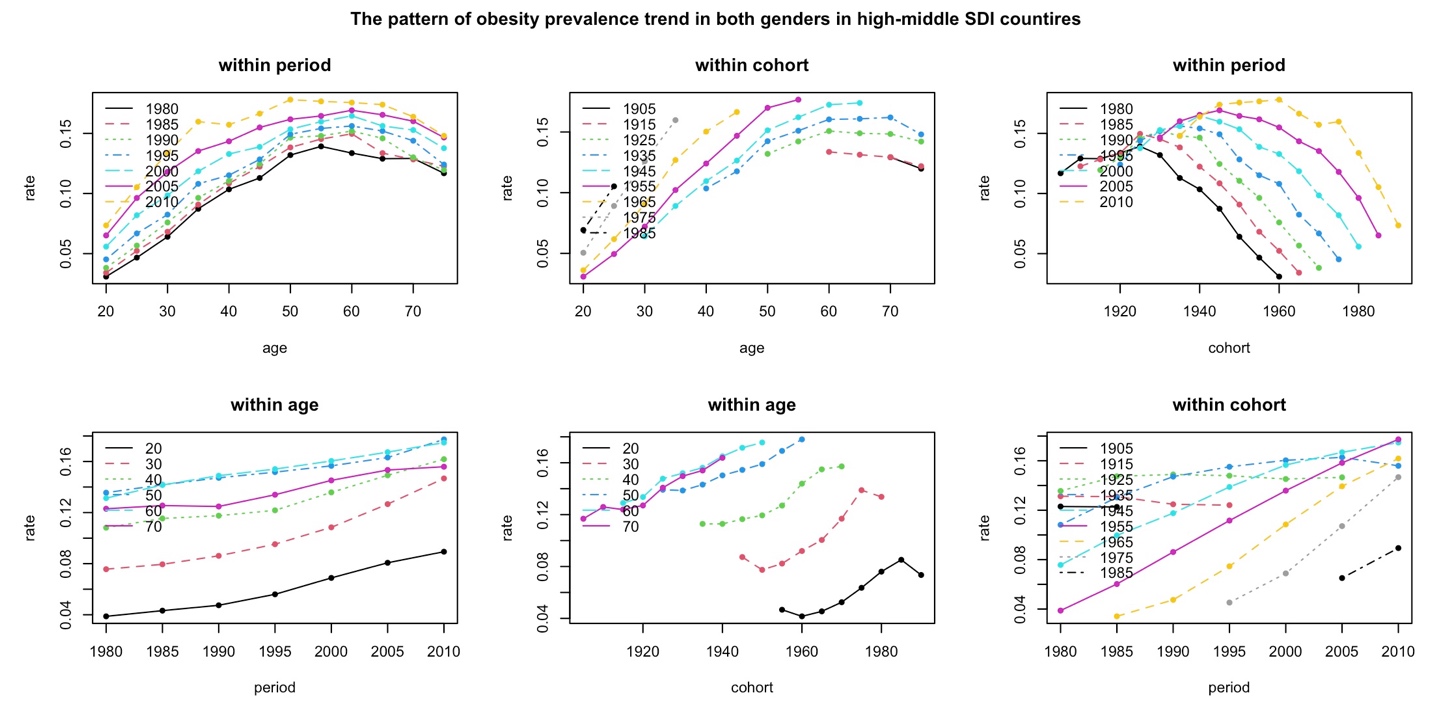


Supplementary Supplementary Figure 4. The trend of obesity prevalence rate within period-age, cohort-age, and period-cohort for both genders in High-Middle SDI countries


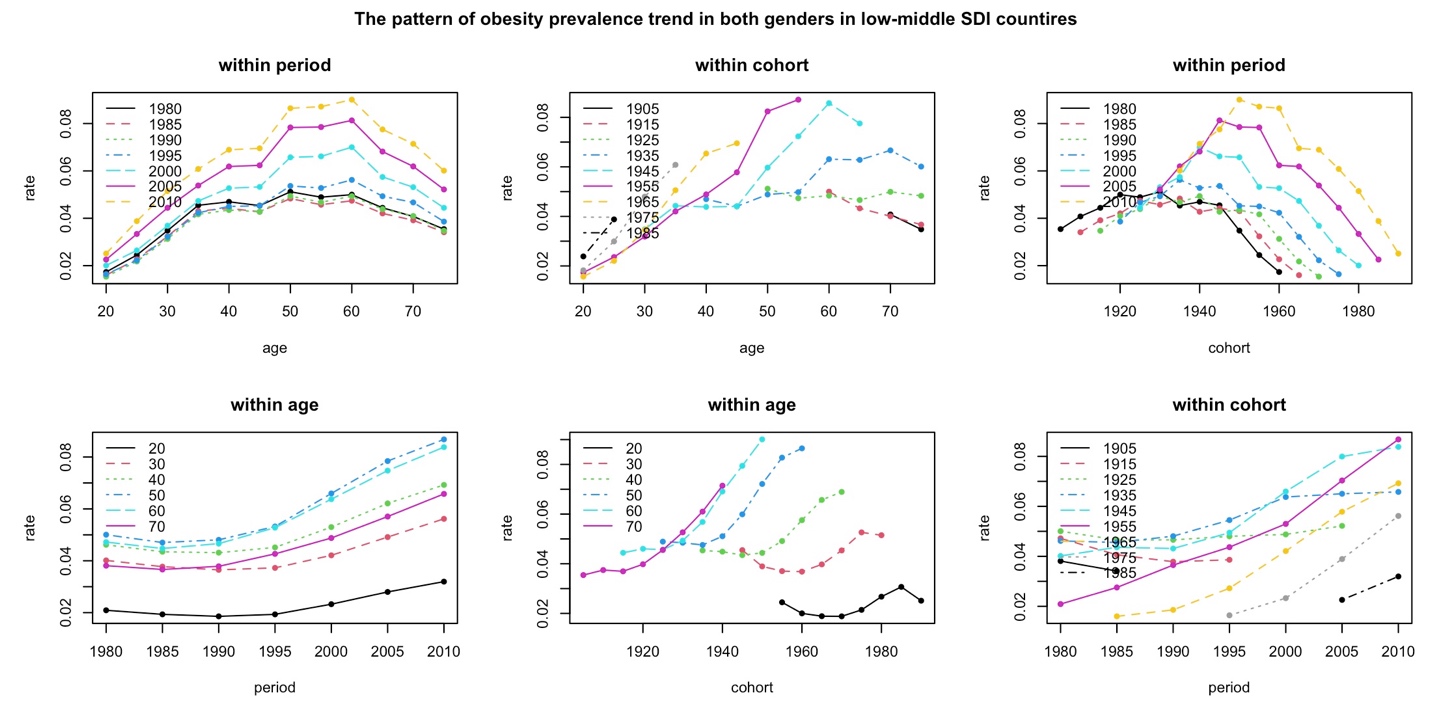


Supplementary Supplementary Figure 5. The trend of obesity prevalence rate within period-age, cohort-age, and period-cohort for both genders in Low-Middle SDI countries


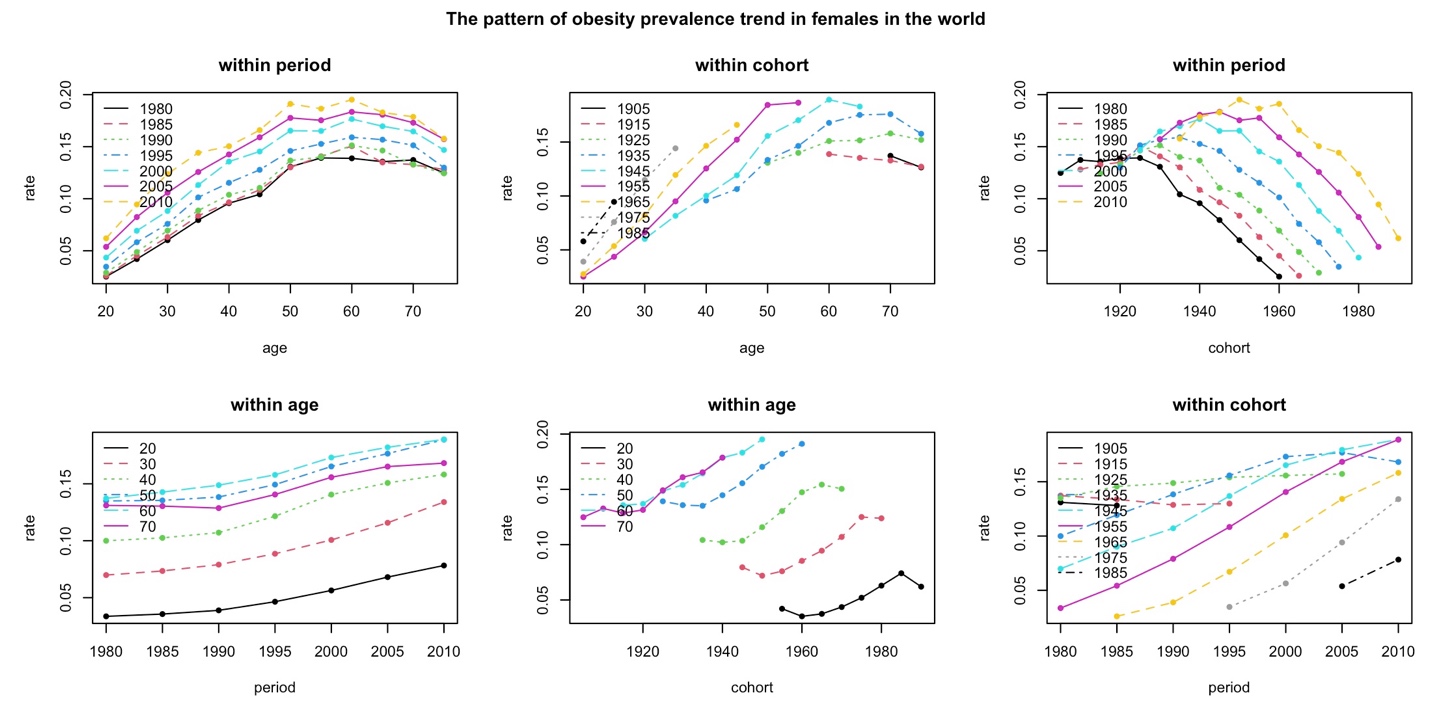


Supplementary Supplementary Figure 6. The trend of obesity prevalence rate within period-age, cohort-age, and period-cohort for females in world.


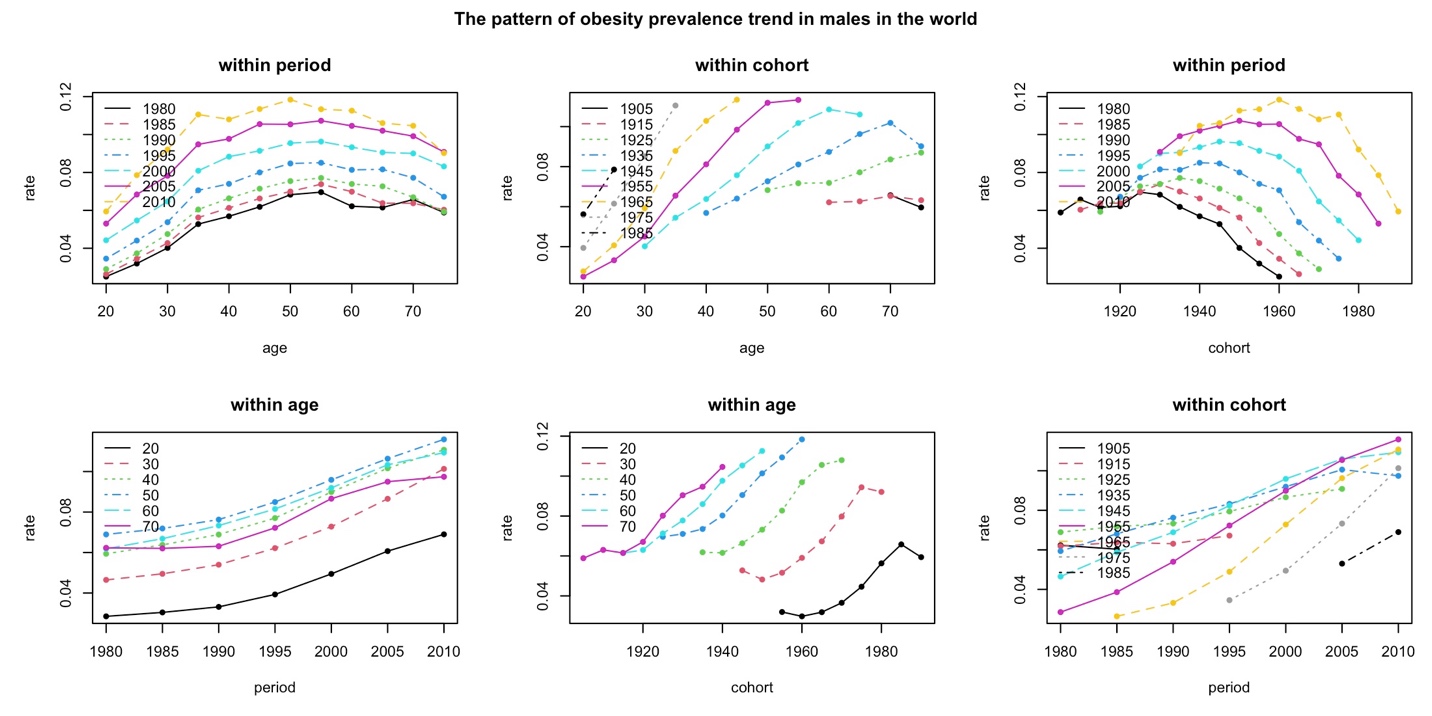


Supplementary Supplementary Figure 7. The trend of obesity prevalence rate within period-age, cohort-age, and period-cohort for males in world


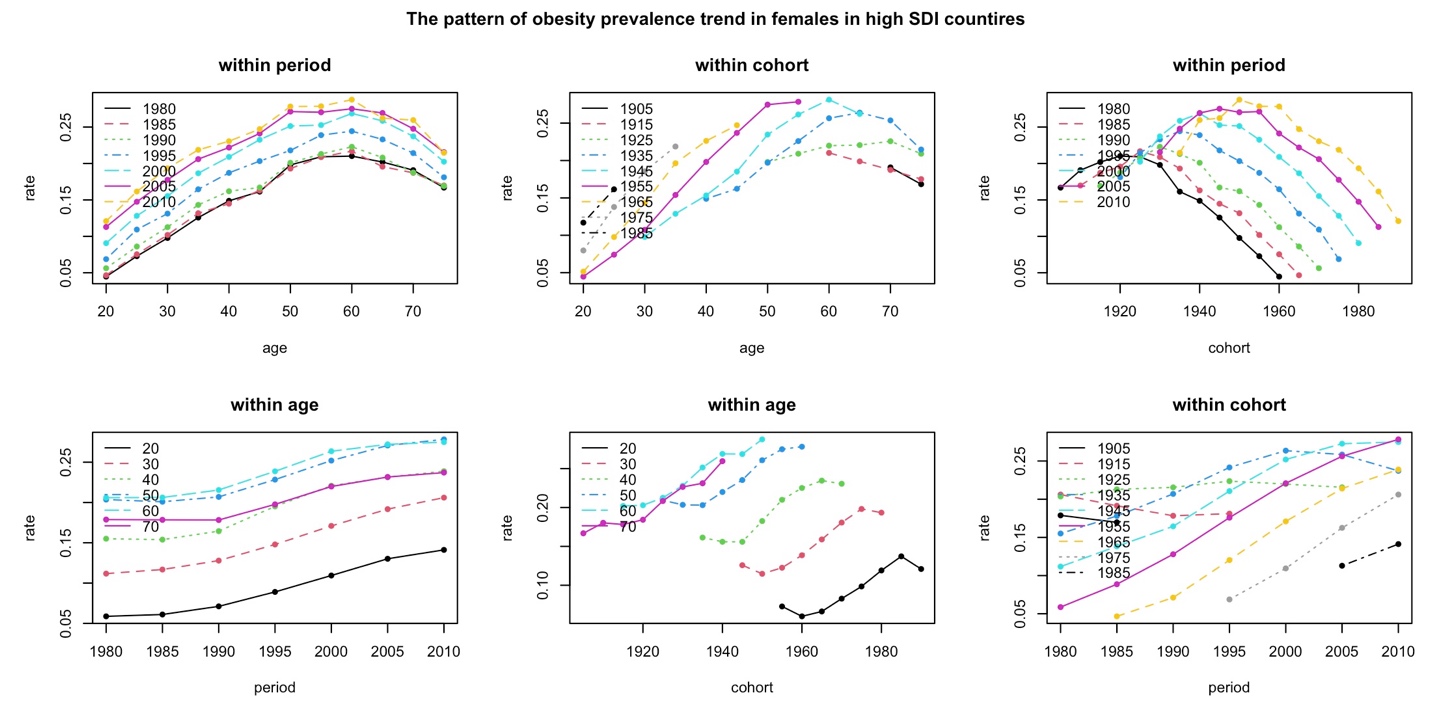


Supplementary Figure 8. The trend of obesity prevalence rate within period-age, cohort-age, and period-cohort for females in High SDI countries.


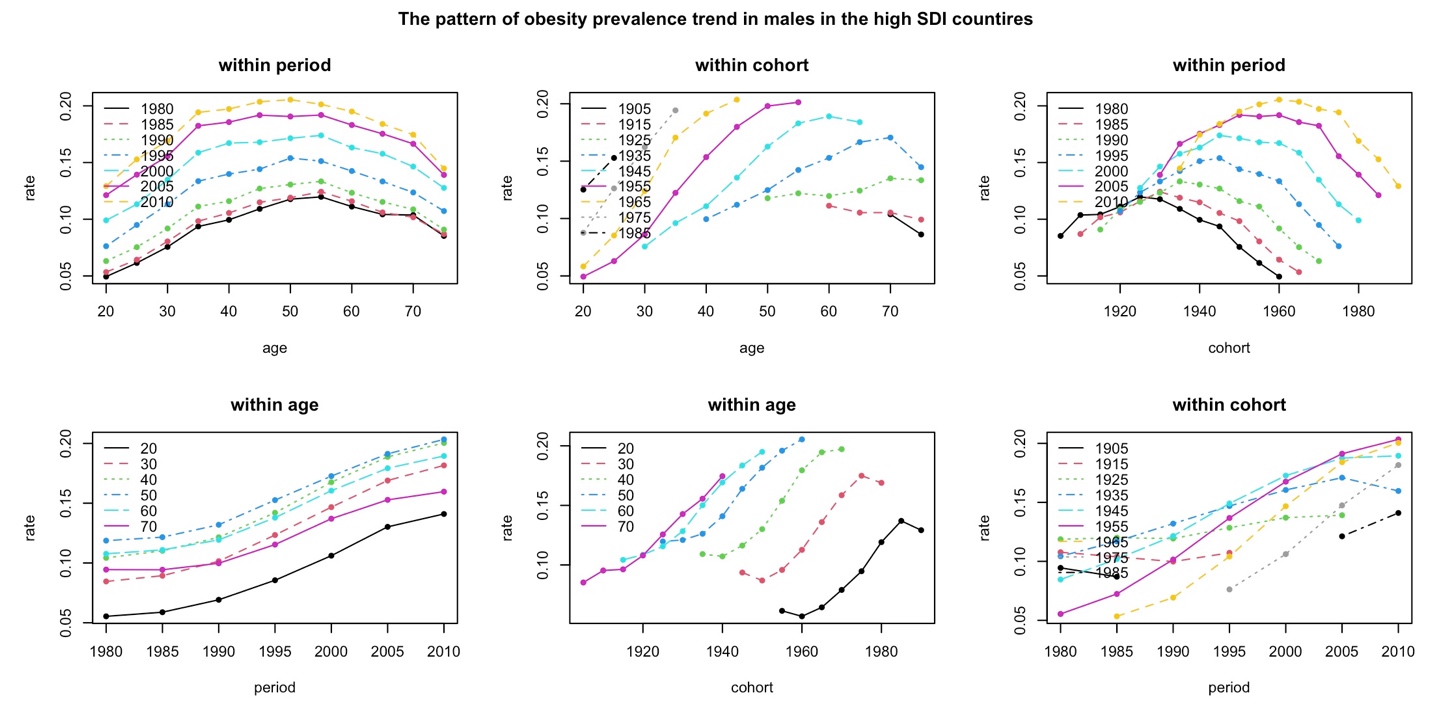


Supplementary Figure 9. The trend of obesity prevalence rate within period-age, cohort-age, and period-cohort for males in High SDI countries.


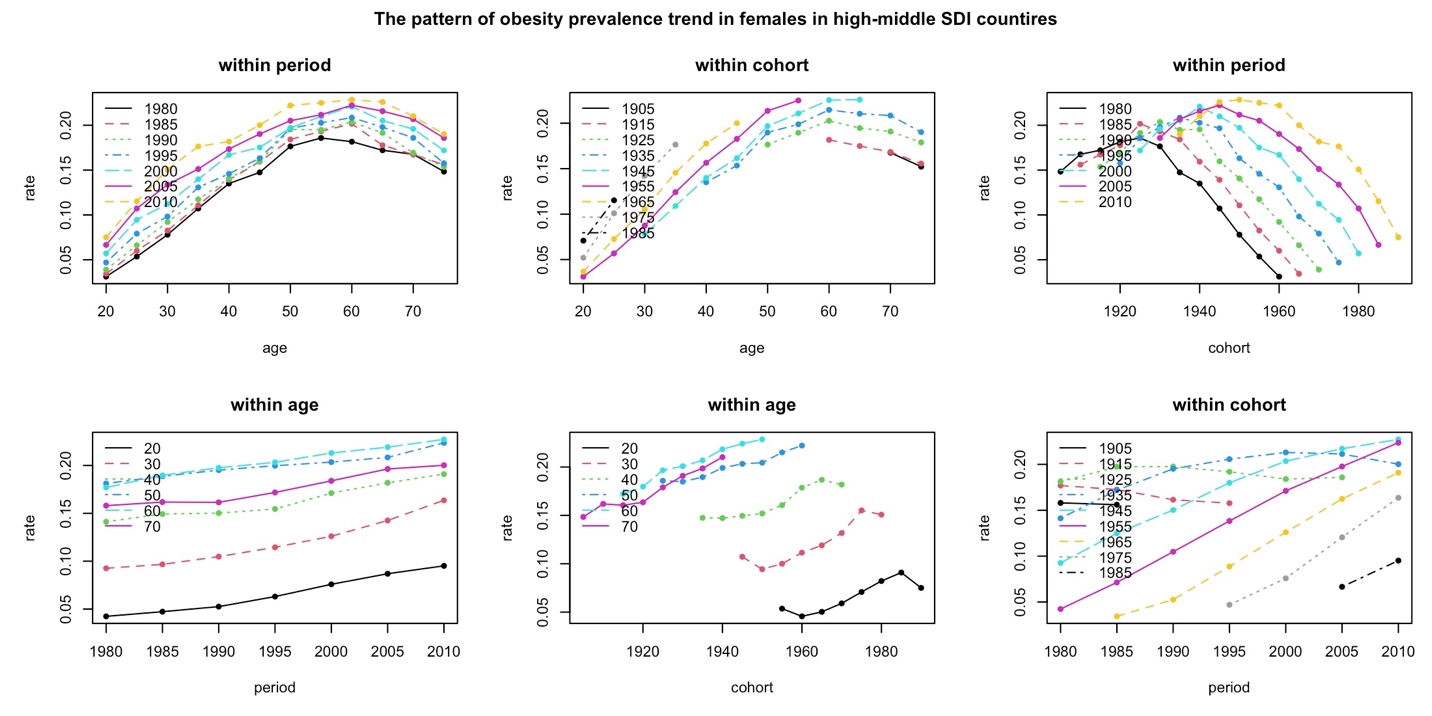


Supplementary Figure 10. The trend of obesity prevalence rate within period-age, cohort-age, and period-cohort for females in High-Middle SDI countries.


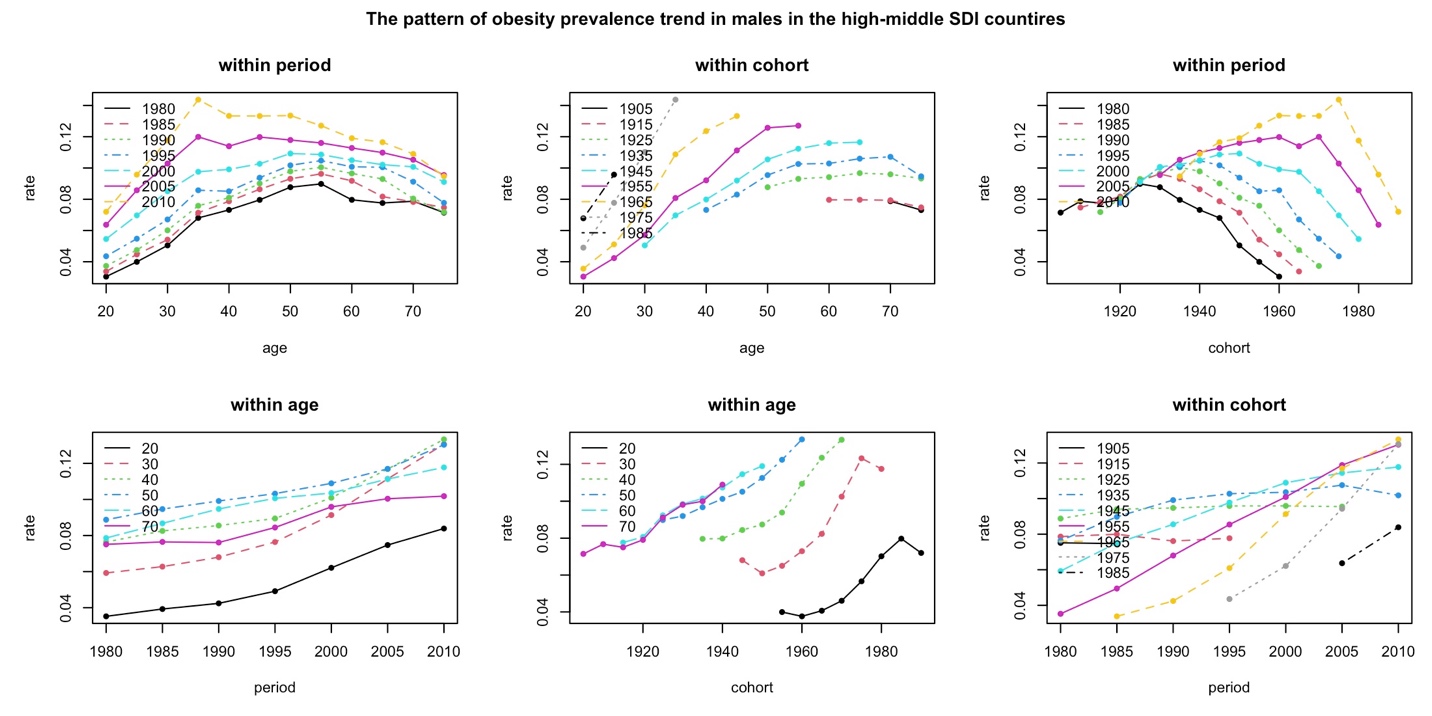


Supplementary Figure 11. The trend of obesity prevalence rate within period-age, cohort-age, and period-cohort for males in High-Middle SDI countries.


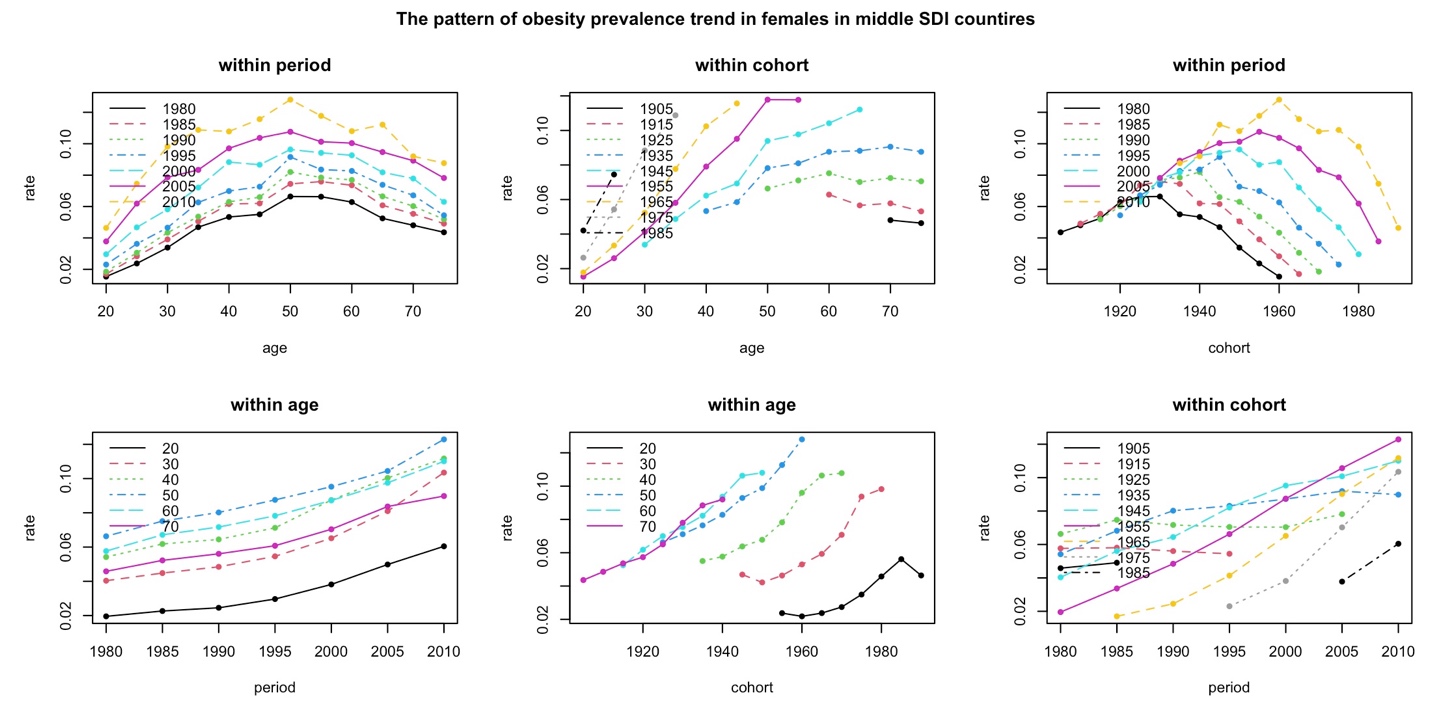


Supplementary Figure 12. The trend of obesity prevalence rate within period-age, cohort-age, and period-cohort for females in Middle SDI countries


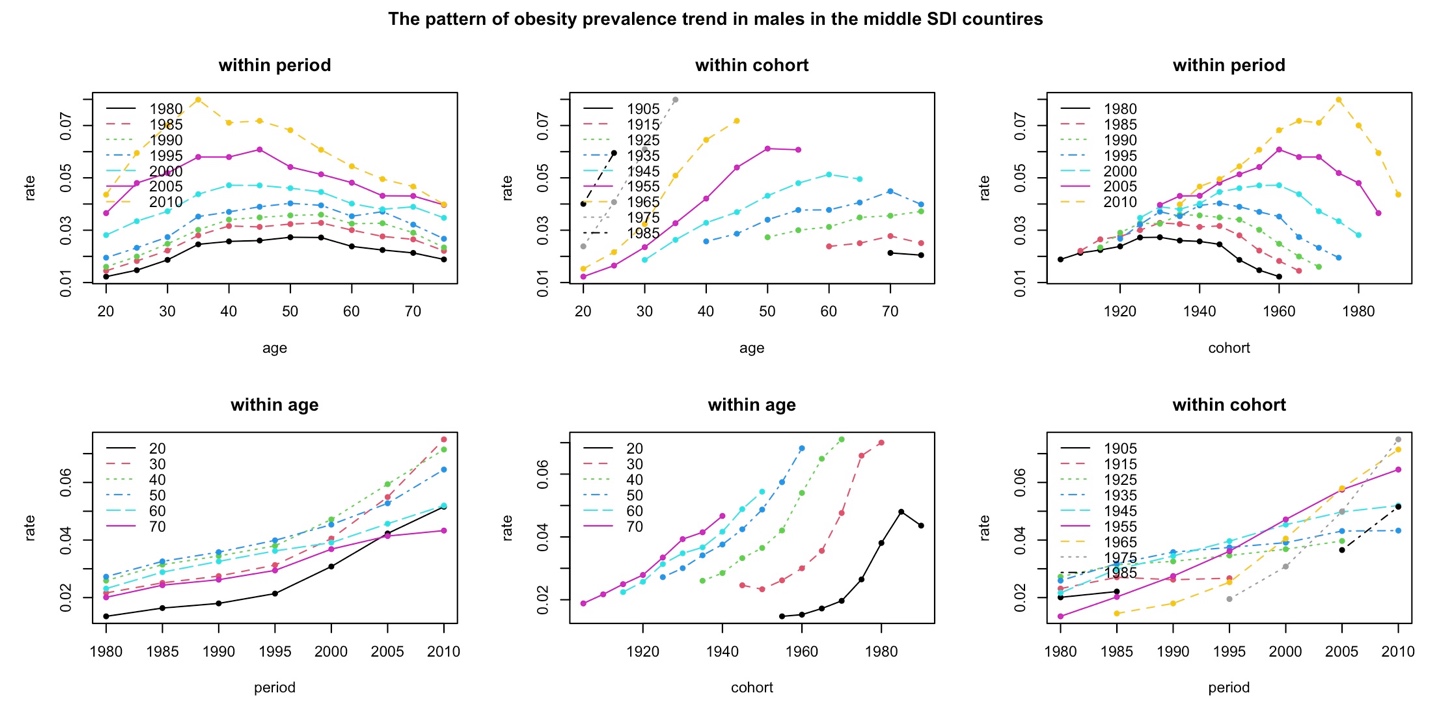


Supplementary Figure 13. The trend of obesity prevalence rate within period-age, cohort-age, and period-cohort for males in Middle SDI countries


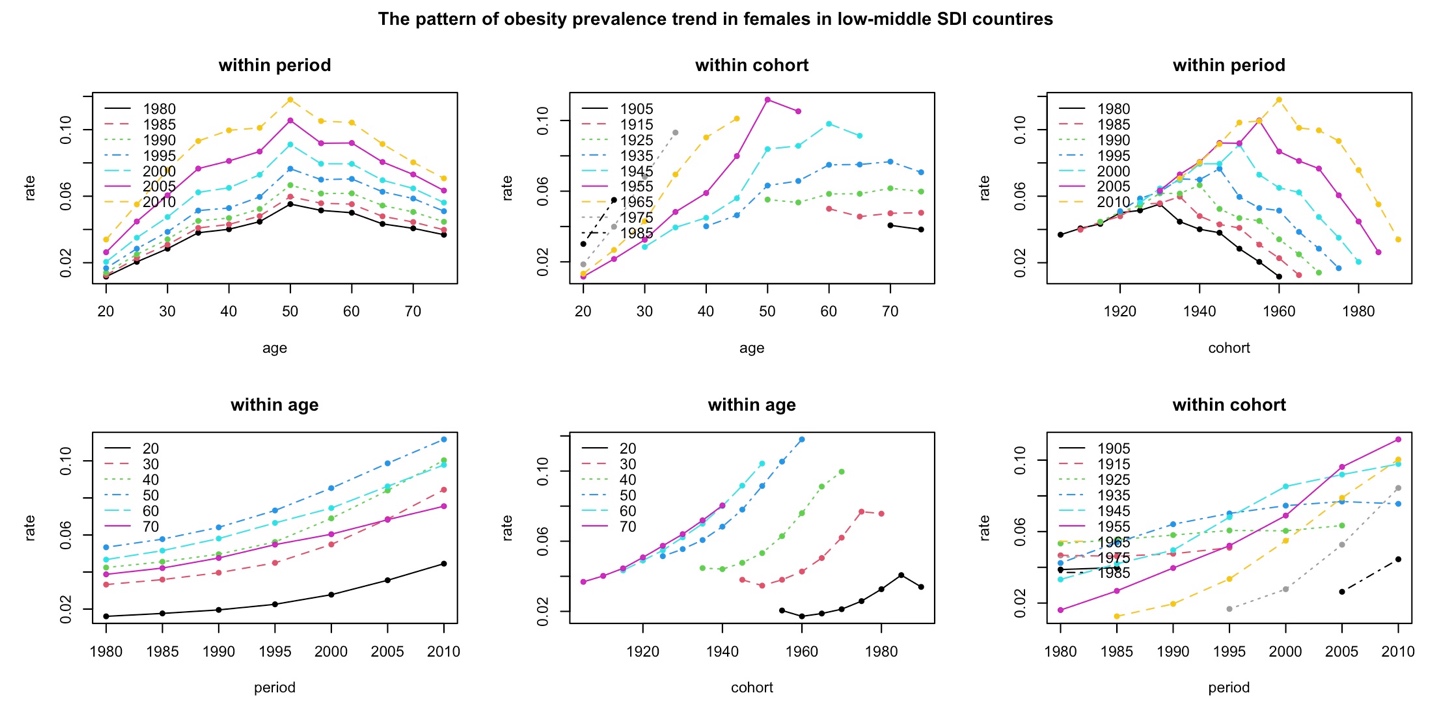


Supplementary Figure 14. The trend of obesity prevalence rate within period-age, cohort-age, and period-cohort for females in Low-Middle SDI countries.


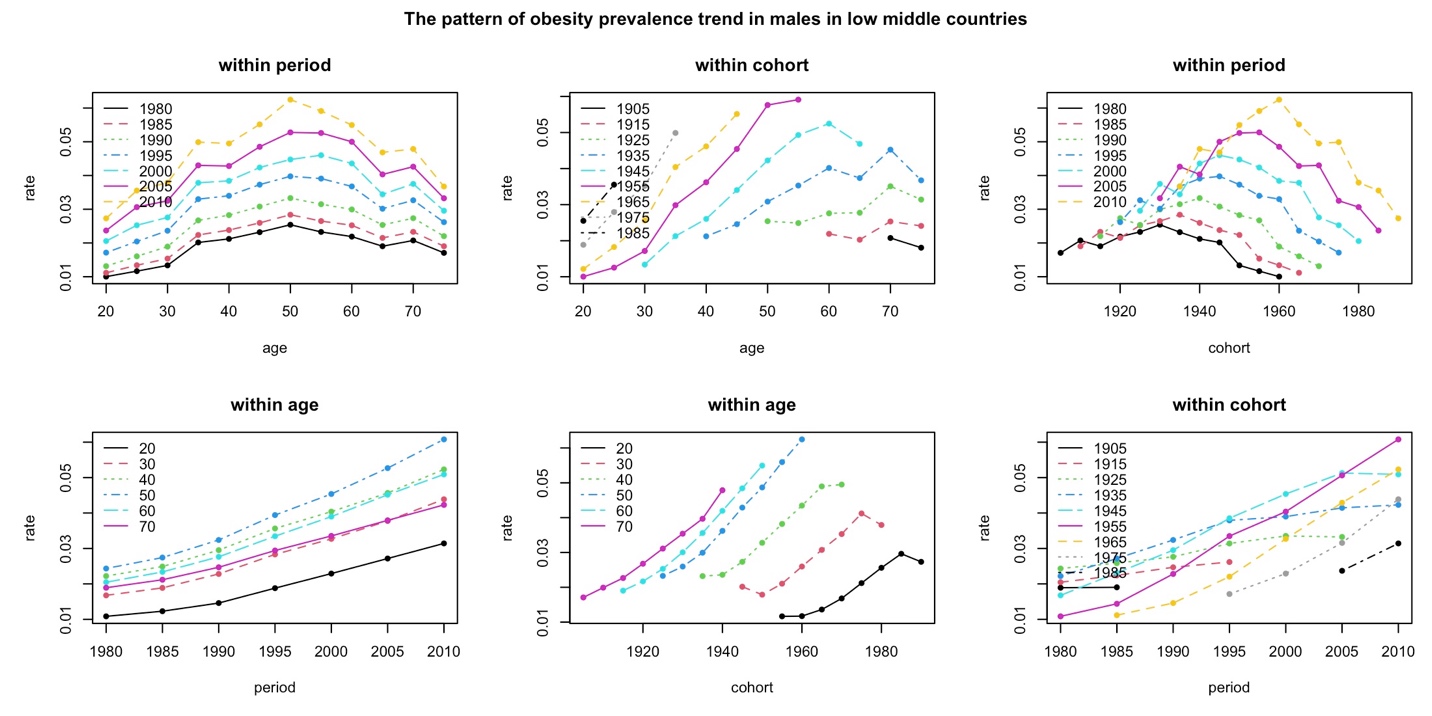


Supplementary Figure 15. The trend of obesity prevalence rate within period-age, cohort-age, and period-cohort for males in Low-Middle SDI countries.


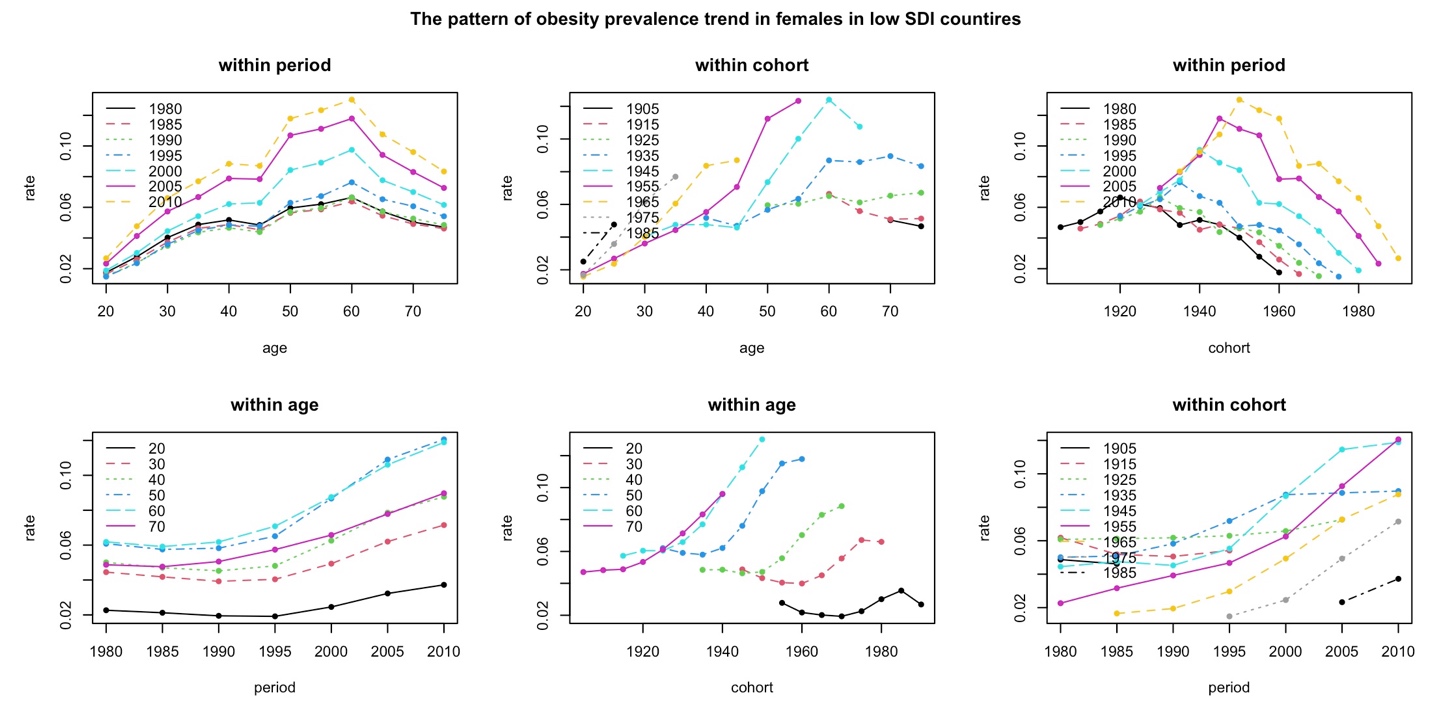


Supplementary Figure 16. The trend of obesity prevalence rate within period-age, cohort-age, and period-cohort for females in Low SDI countries


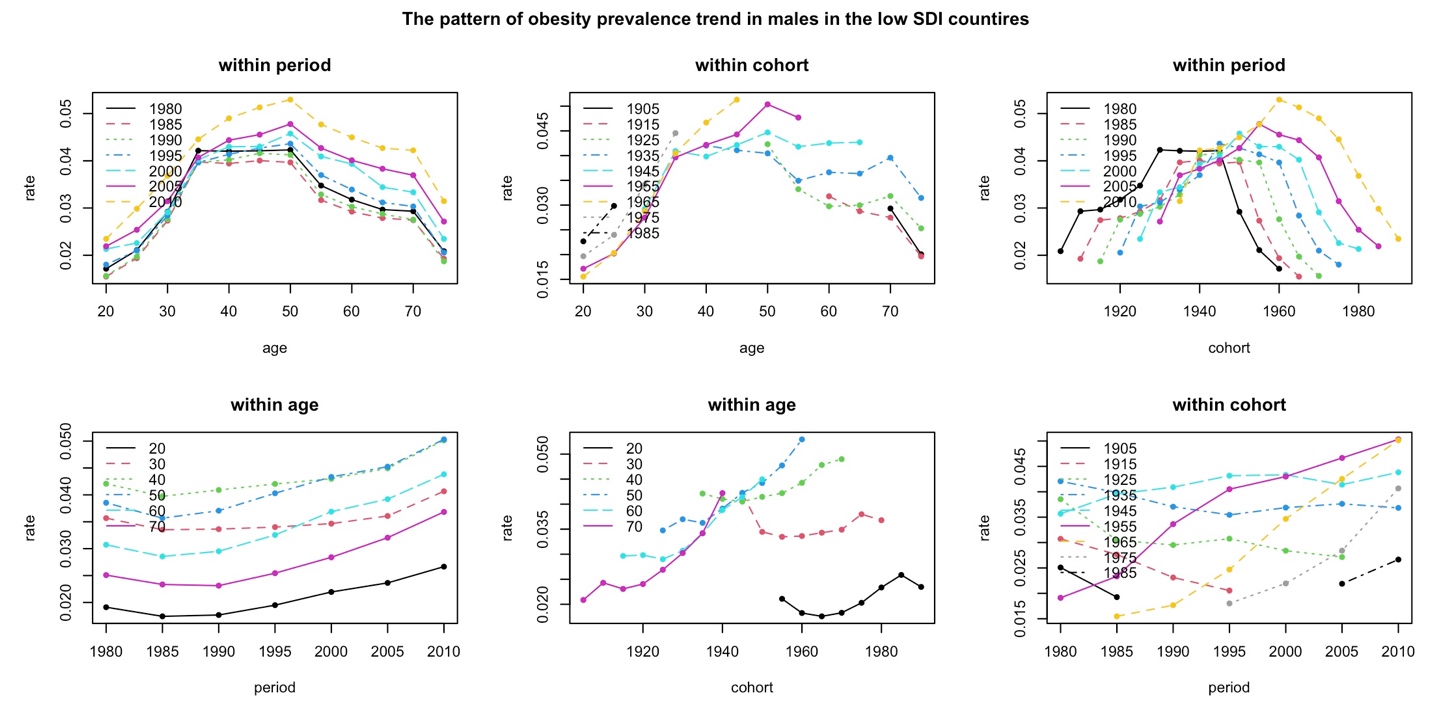


Supplementary Figure 17. The trend of obesity prevalence rate within period-age, cohort-age, and period-cohort for males in Low SDI countries


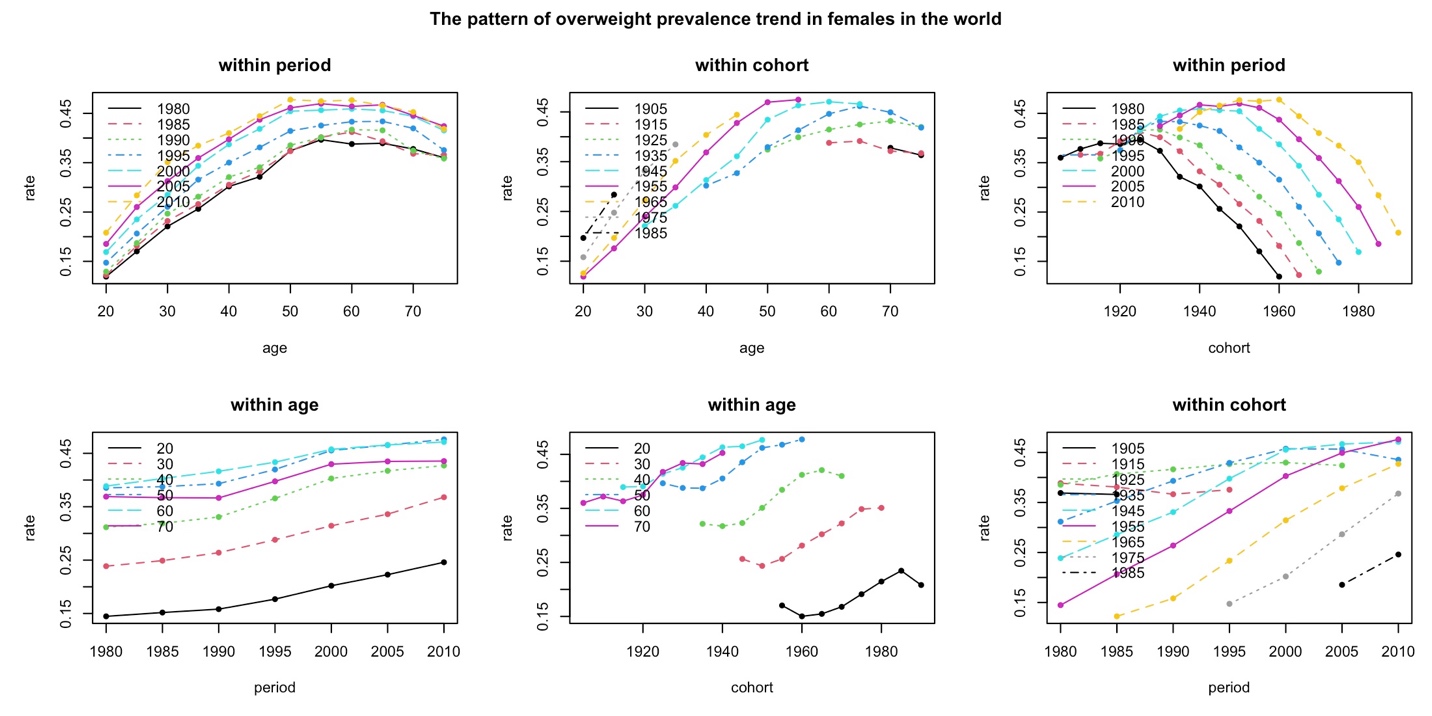


Supplementary Figure 18. The trend of overweight prevalence rate within period-age, cohort-age, and period-cohort for females in the world


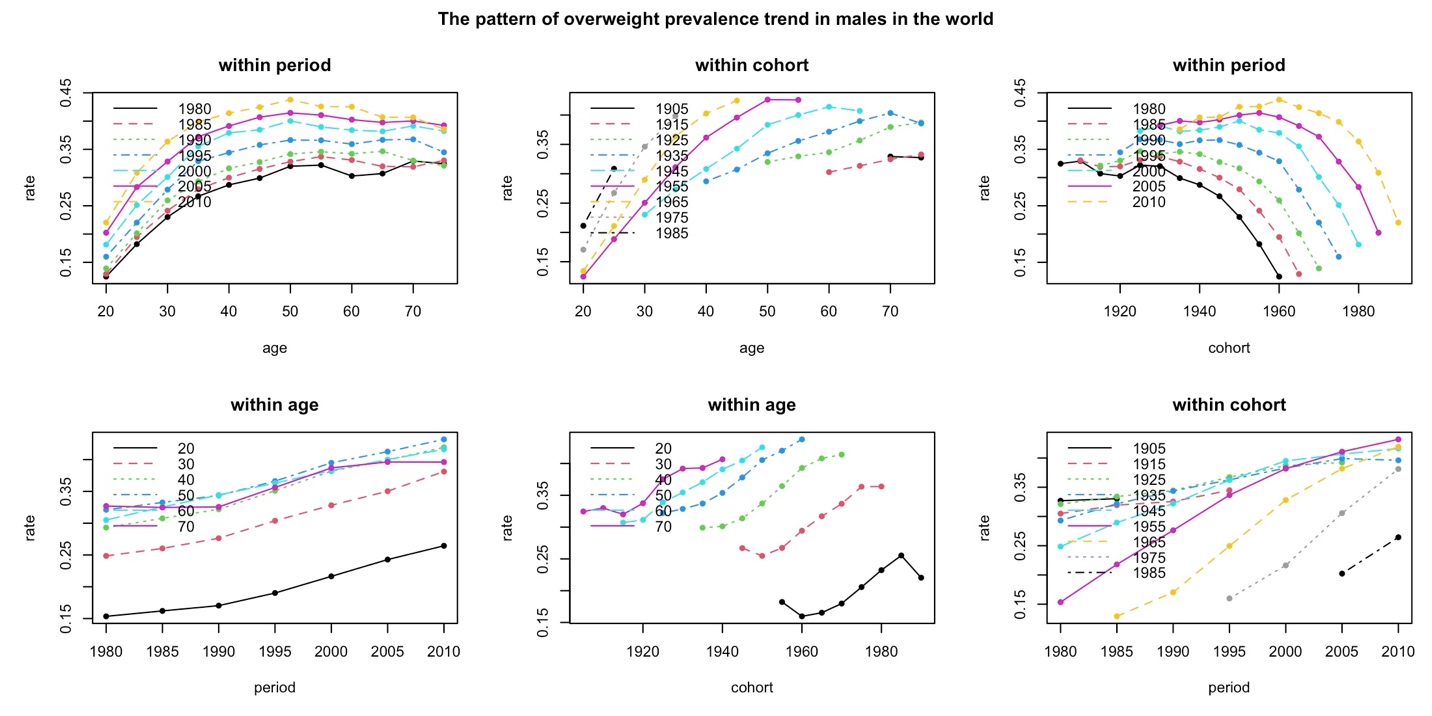


Supplementary Figure 19. The trend of overweight prevalence rate within period-age, cohort-age, and period-cohort for males in the world


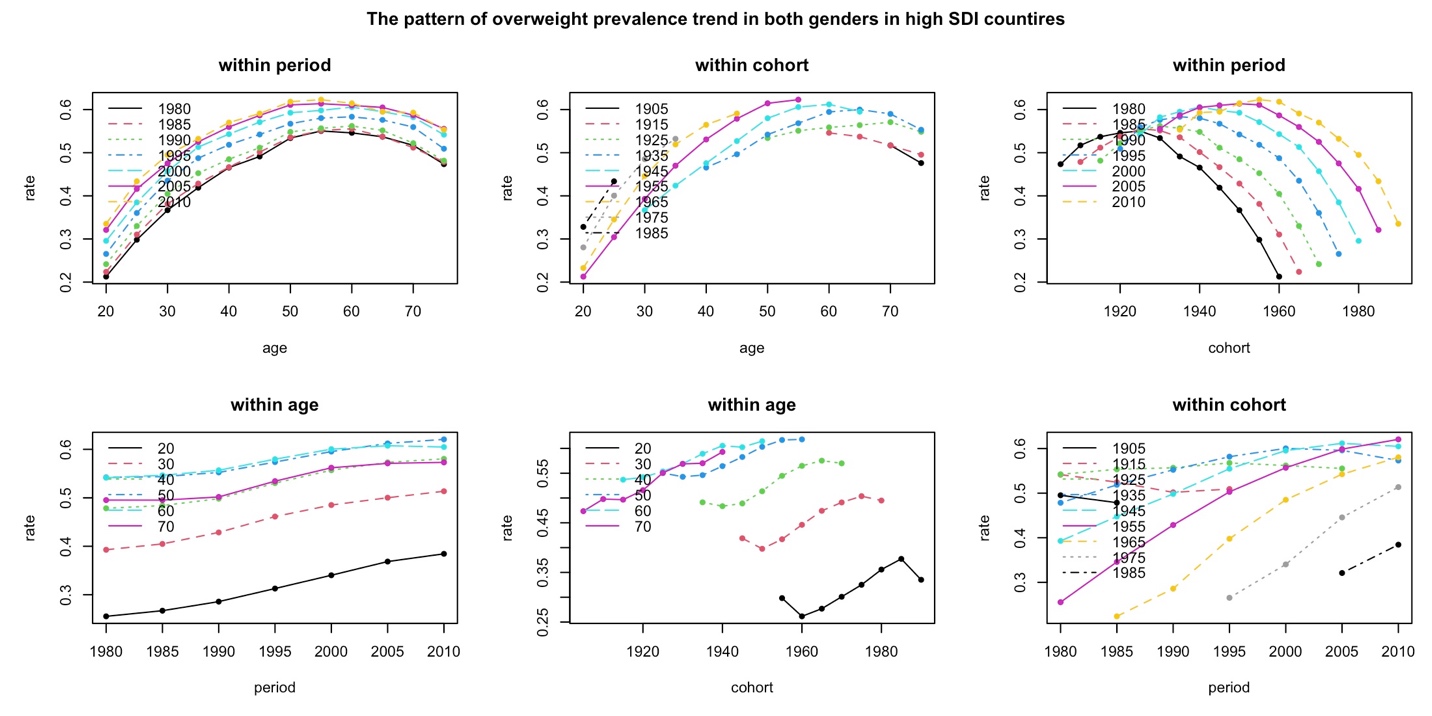


Supplementary Figure 20. The trend of overweight prevalence rate within period-age, cohort-age, and period-cohort for both sexes in High SDI countries.


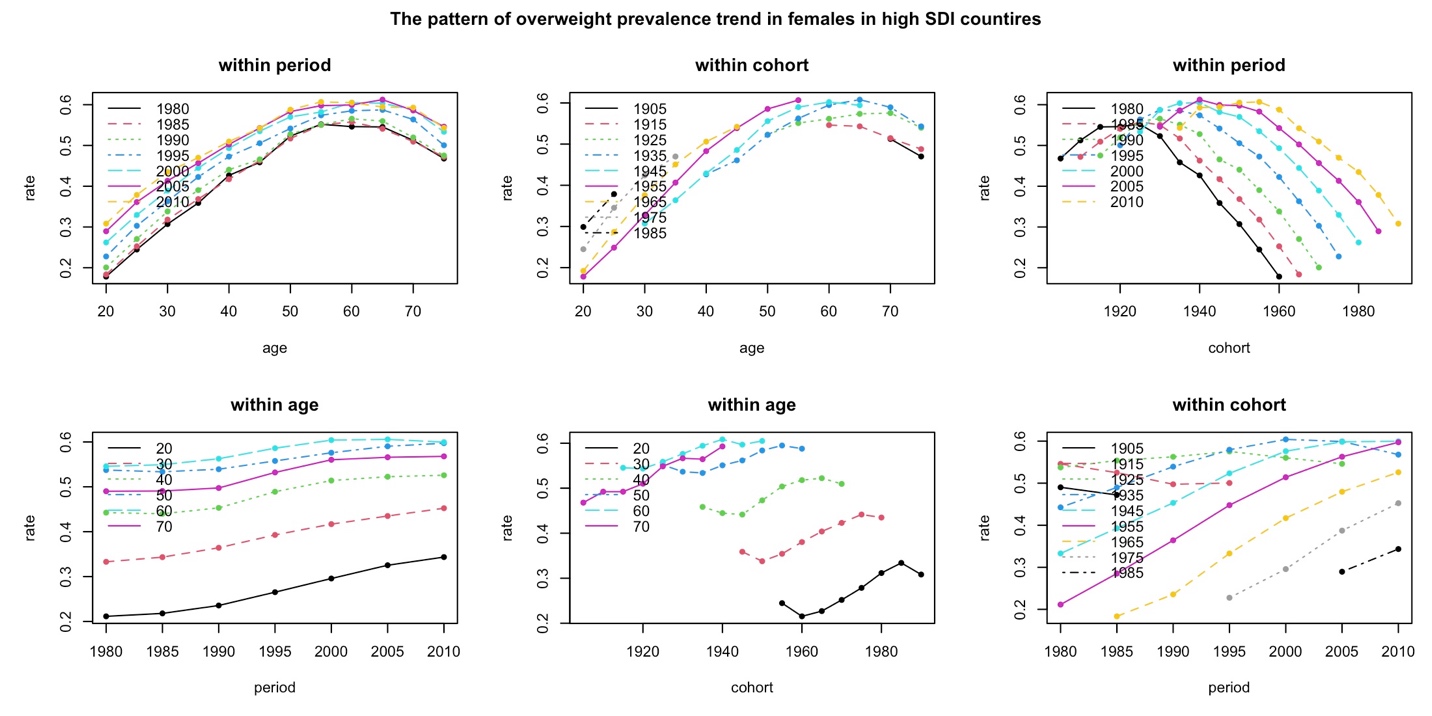


Supplementary Figure 21. The trend of overweight prevalence rate within period-age, cohort-age, and period-cohort for females in High SDI countries


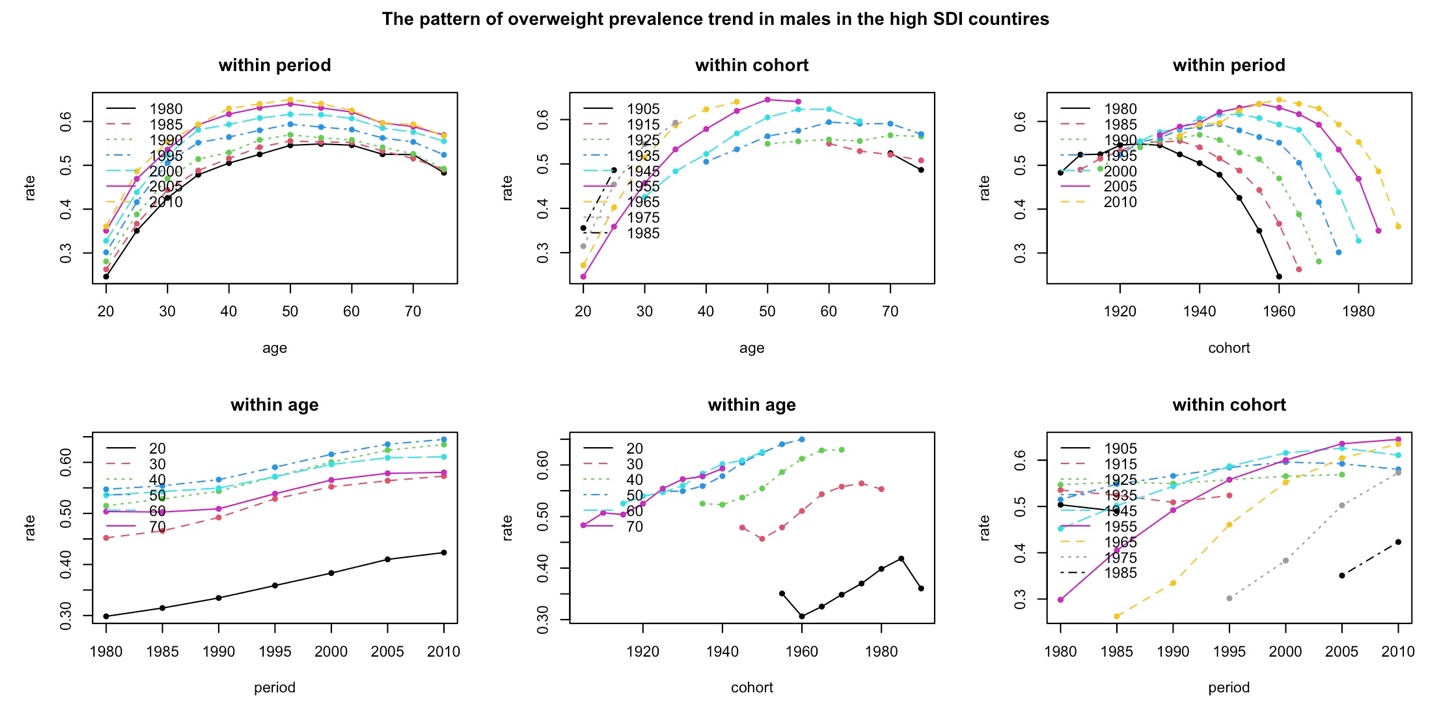


Supplementary Figure 22. The trend of overweight prevalence rate within period-age, cohort-age, and period-cohort for males in High SDI countries


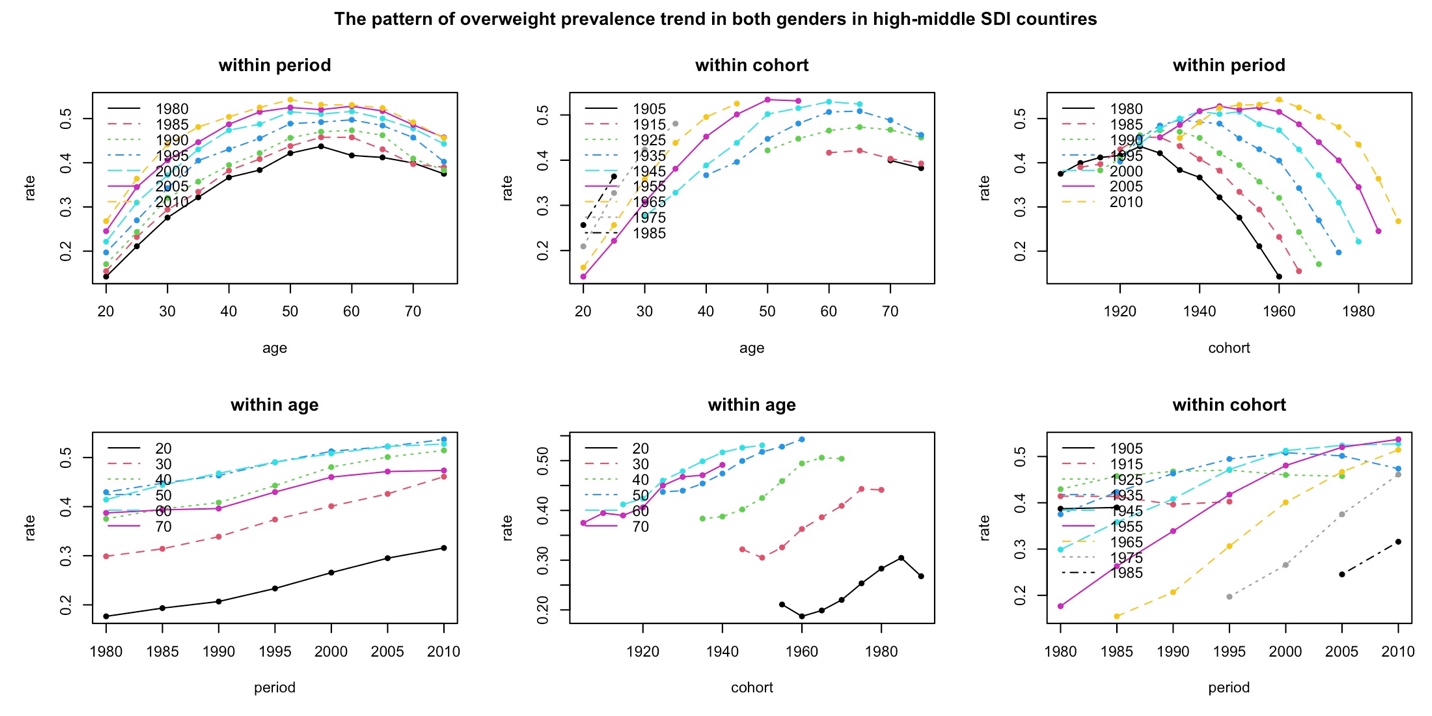


Supplementary Figure 23. The trend of overweight prevalence rate within period-age, cohort-age, and period-cohort for both genders in High-Middle SDI countries


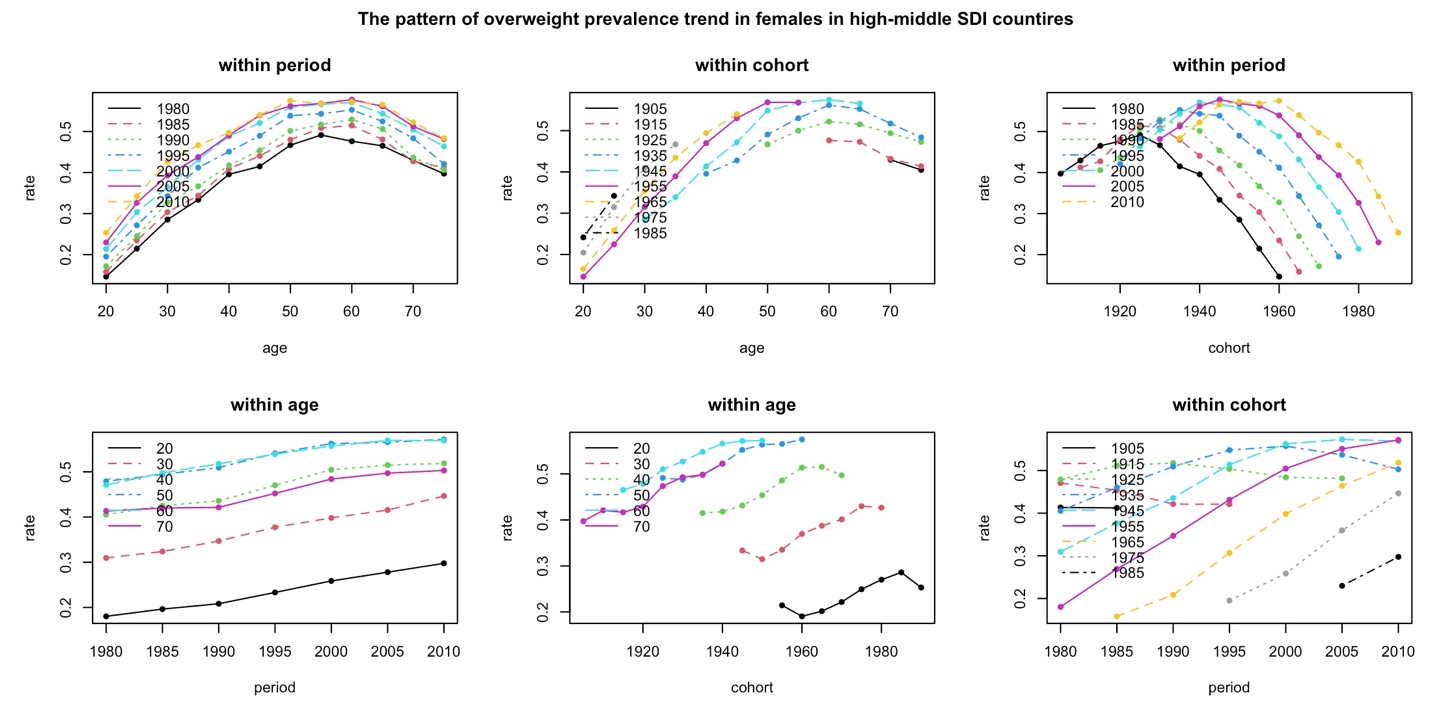


Supplementary Figure 24. The trend of overweight prevalence rate within period-age, cohort-age, and period-cohort for females in High-Middle SDI countries


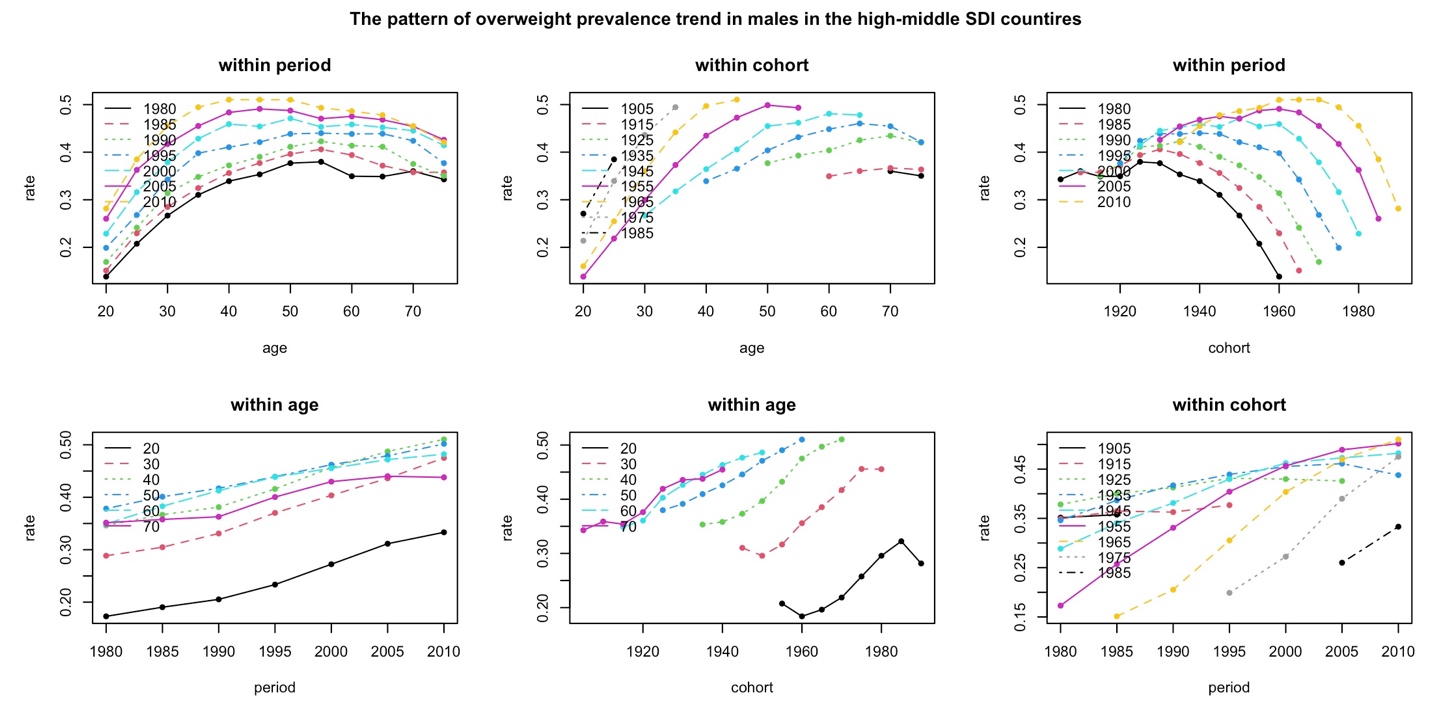


Supplementary Figure 25. The trend of overweight prevalence rate within period-age, cohort-age, and period-cohort for males in High-Middle SDI countries


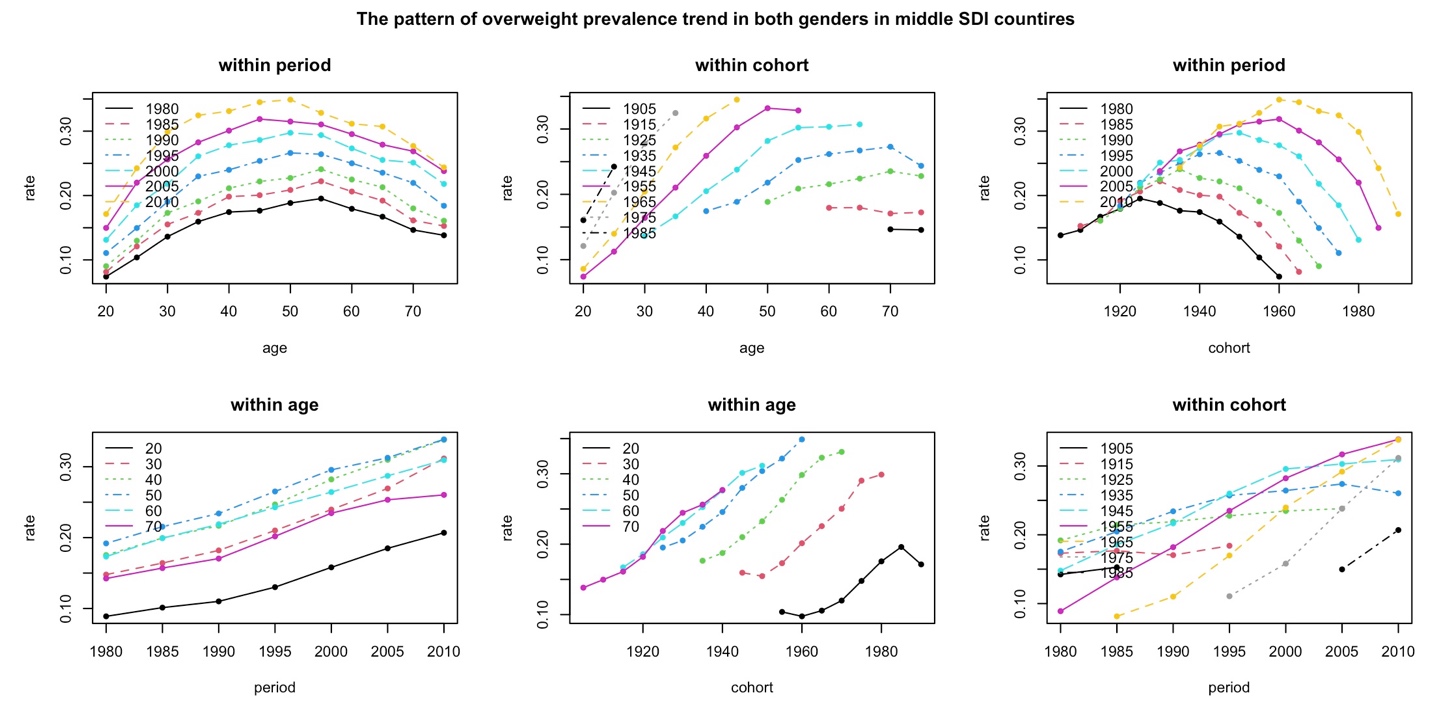


Supplementary Figure 26. The trend of overweight prevalence rate within period-age, cohort-age, and period-cohort for both genders in Middle SDI countries


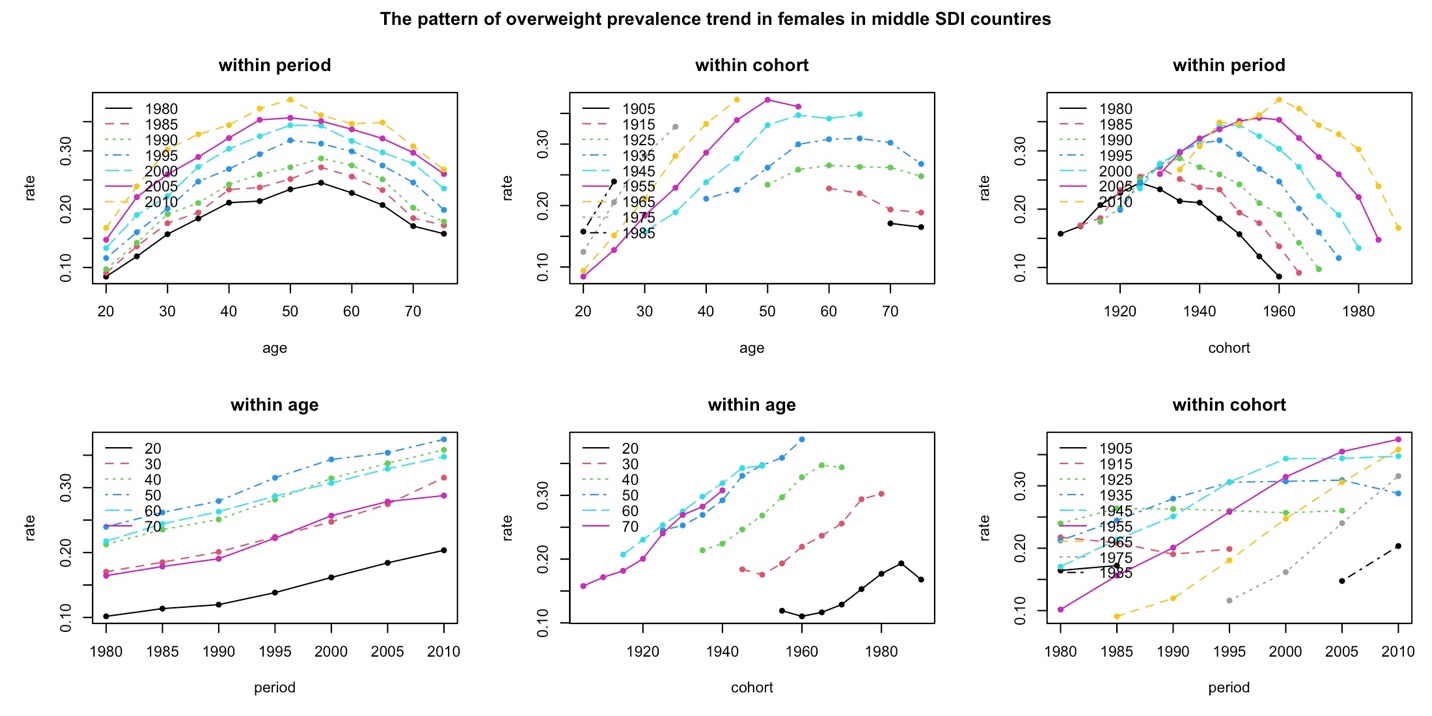


Supplementary Figure 27. The trend of overweight prevalence rate within period-age, cohort-age, and period-cohort for females in Middle SDI countries


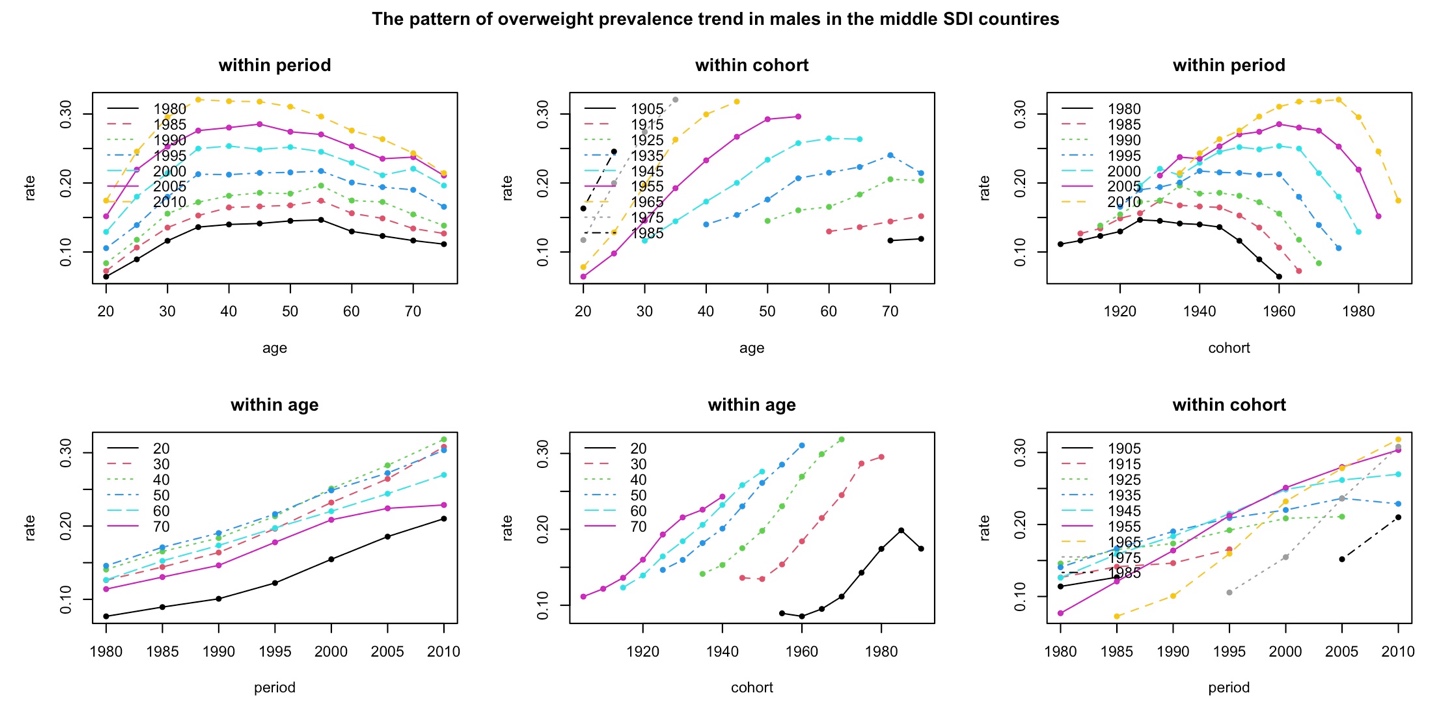


Supplementary Figure 28. The trend of overweight prevalence rate within period-age, cohort-age, and period-cohort for males in Middle SDI countries


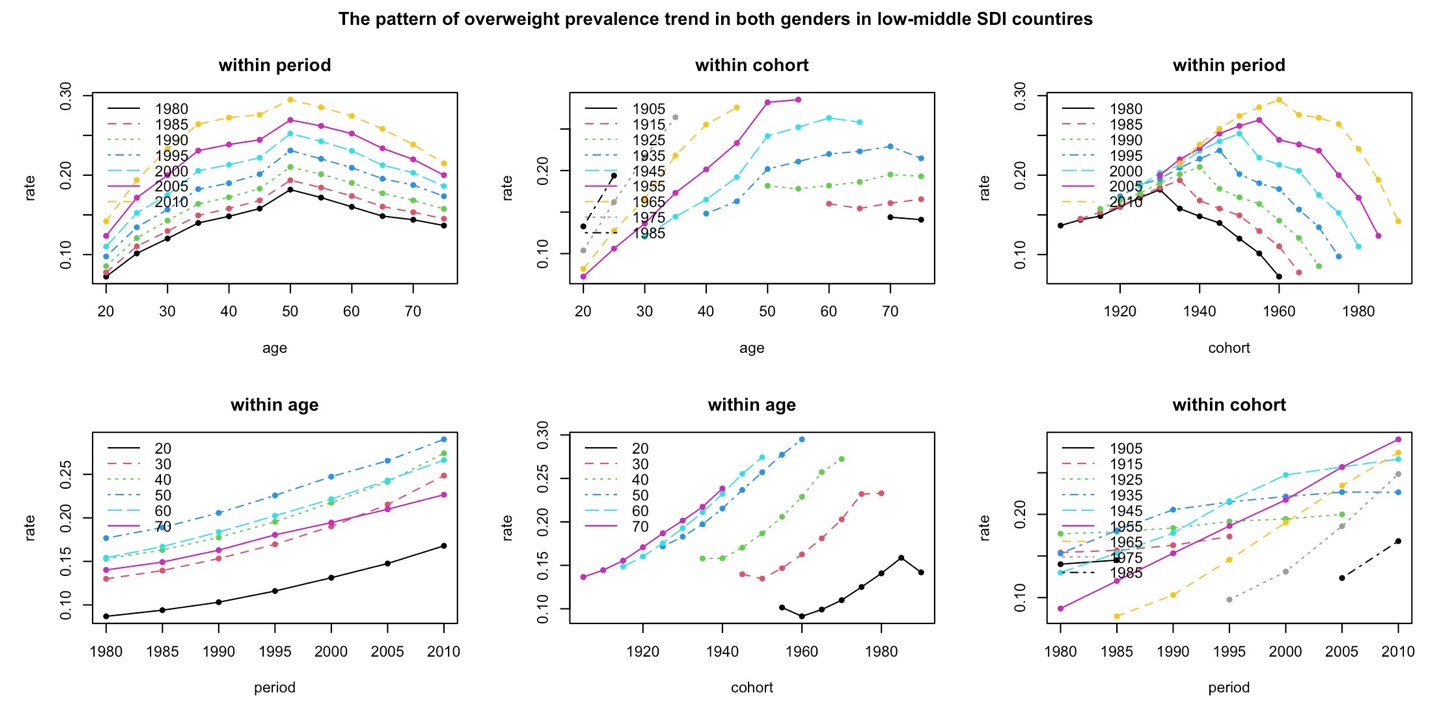


Supplementary Figure 29. The trend of overweight prevalence rate within period-age, cohort-age, and period-cohort for both genders in Low-Middle SDI countries


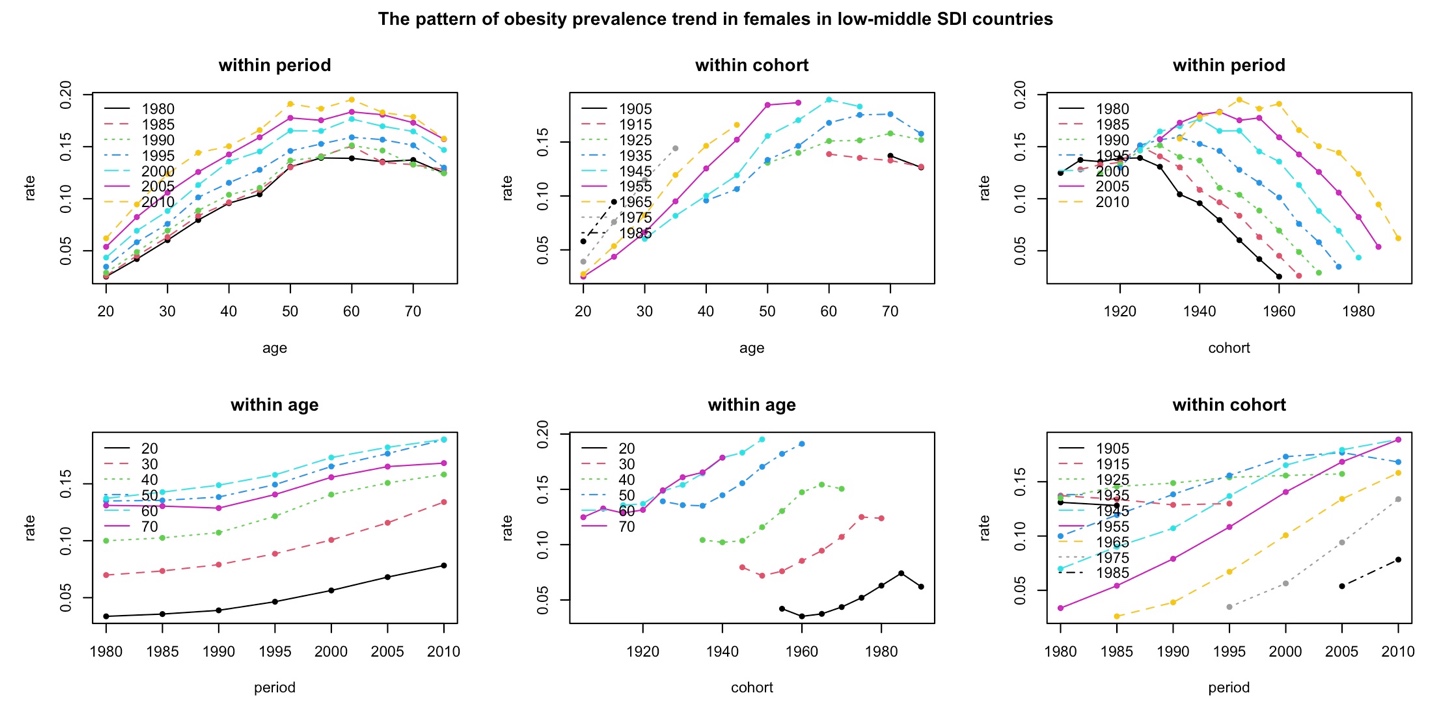


Supplementary Figure 30. The trend of overweight prevalence rate within period-age, cohort-age, and period-cohort for females in Low-Middle SDI countries.


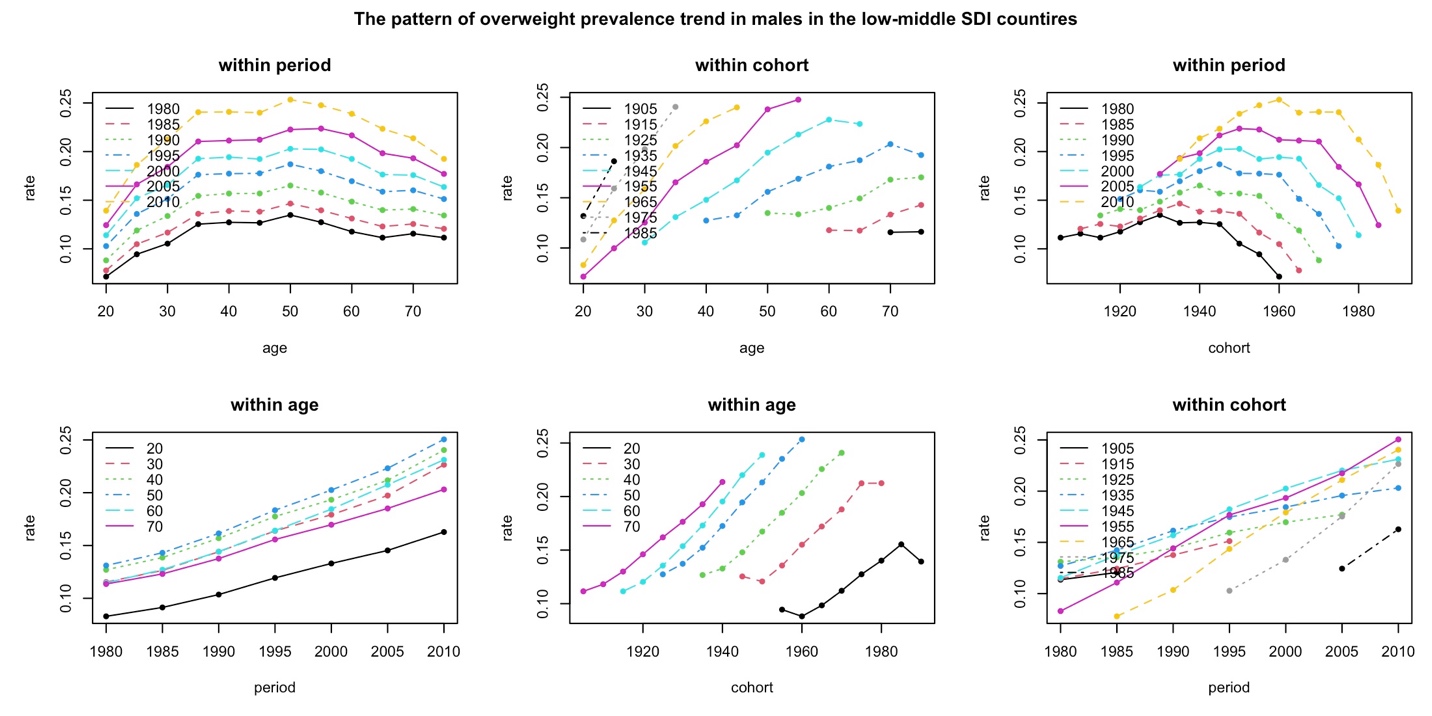


Supplementary Figure 31. The trend of overweight prevalence rate within period-age, cohort-age, and period-cohort for males in Low-Middle SDI countries


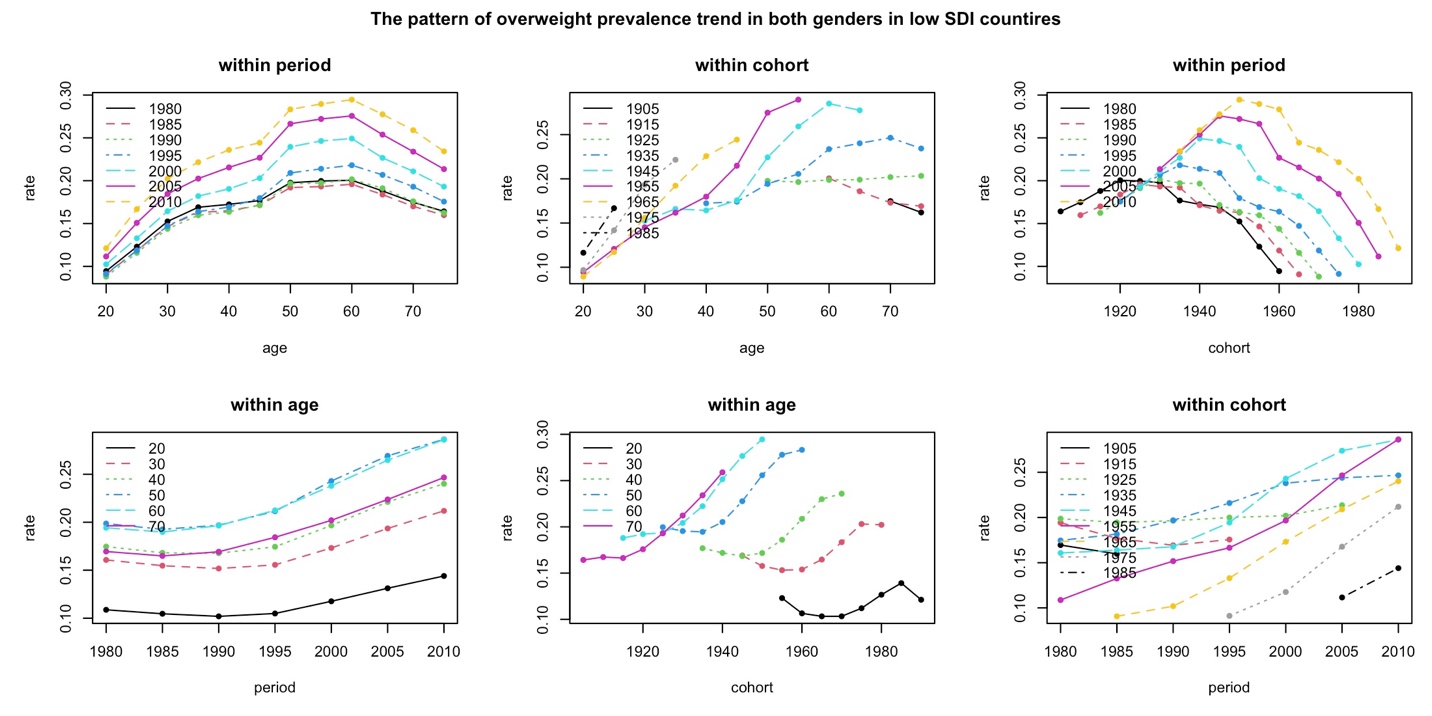


Supplementary Figure 32. The trend of overweight prevalence rate within period-age, cohort-age, and period-cohort for both genders in Low SDI countries.


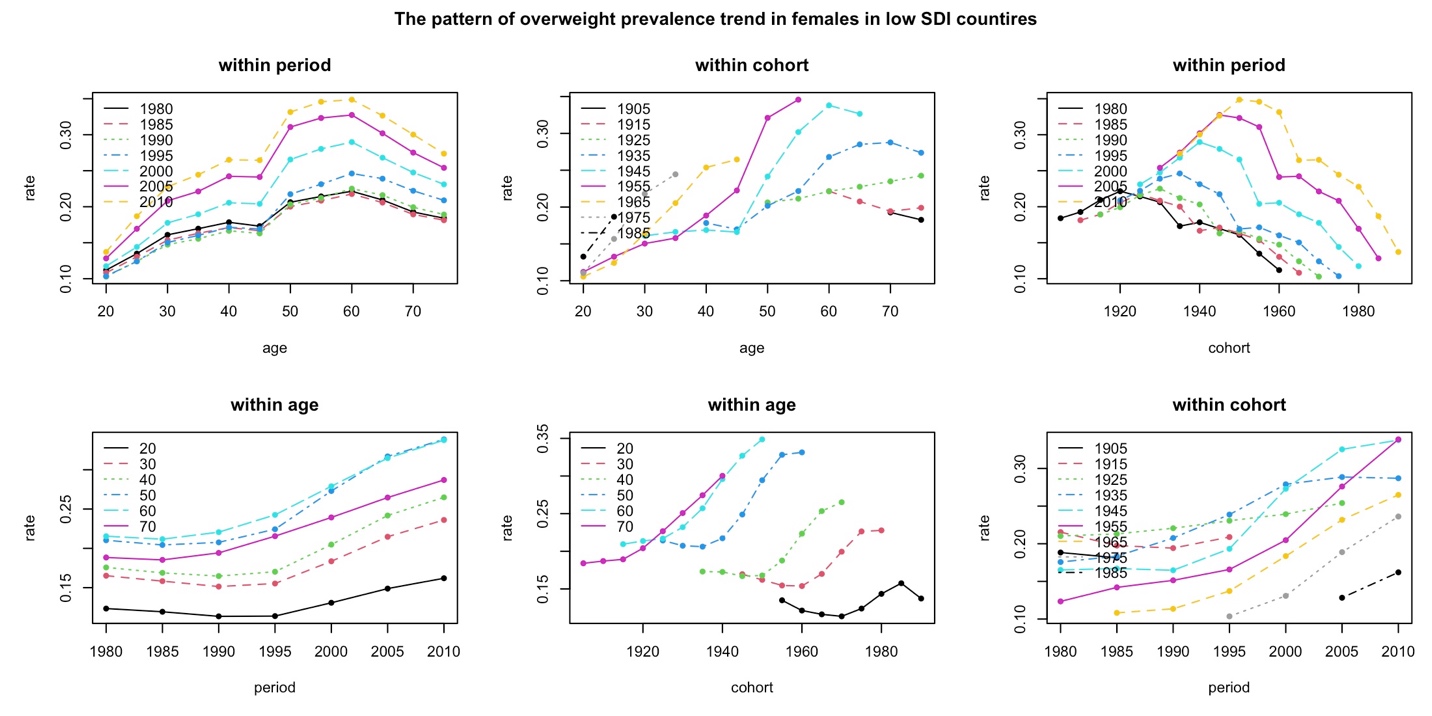


Supplementary Figure 33. The trend of overweight prevalence rate within period-age, cohort-age, and period-cohort for females in Low SDI countries


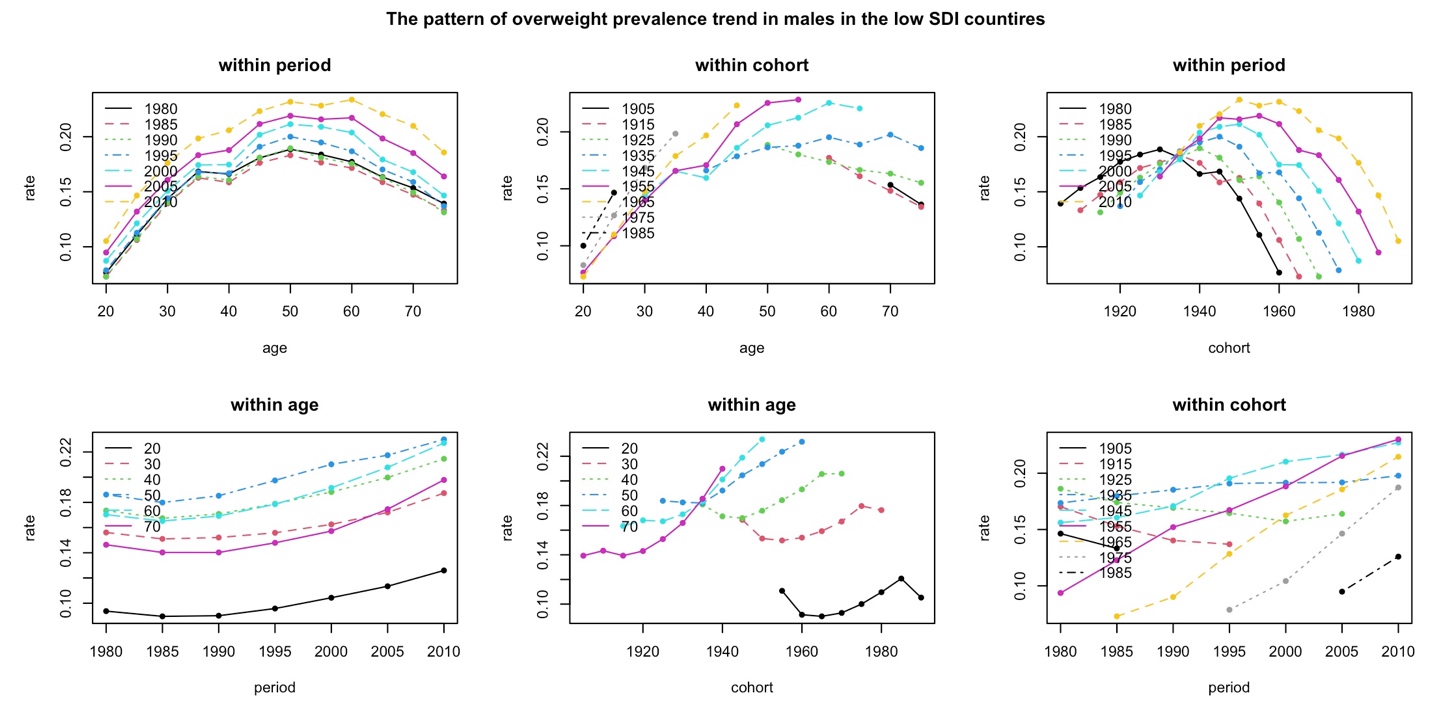


Supplementary Figure 34. The trend of overweight prevalence rate within period-age, cohort-age, and period-cohort for males in Low SDI countries.


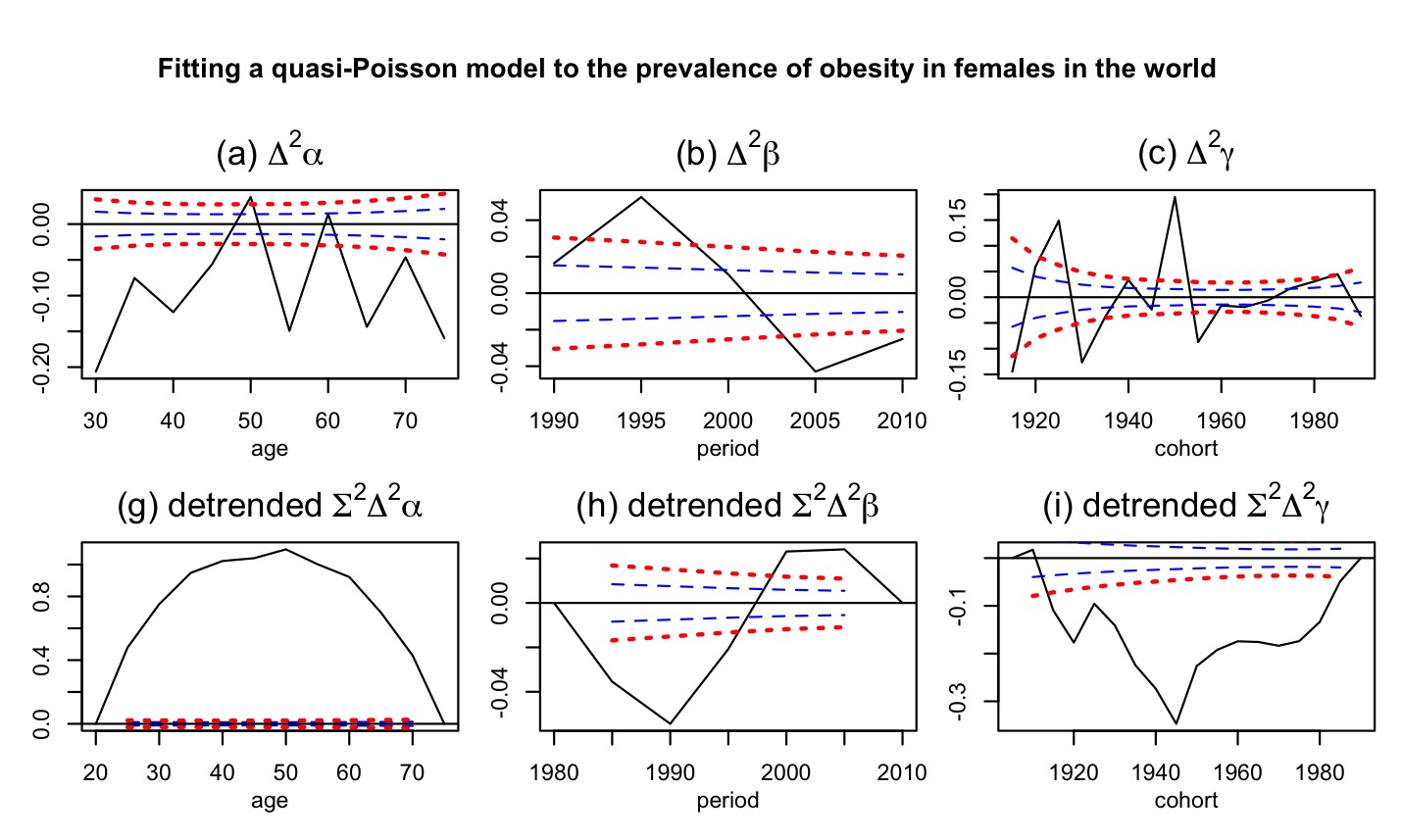


Supplementary Figure 35. Plots of the results of the APC model fit on obesity prevalence in females in the world. A quasi-Poisson model was fitted to the data.


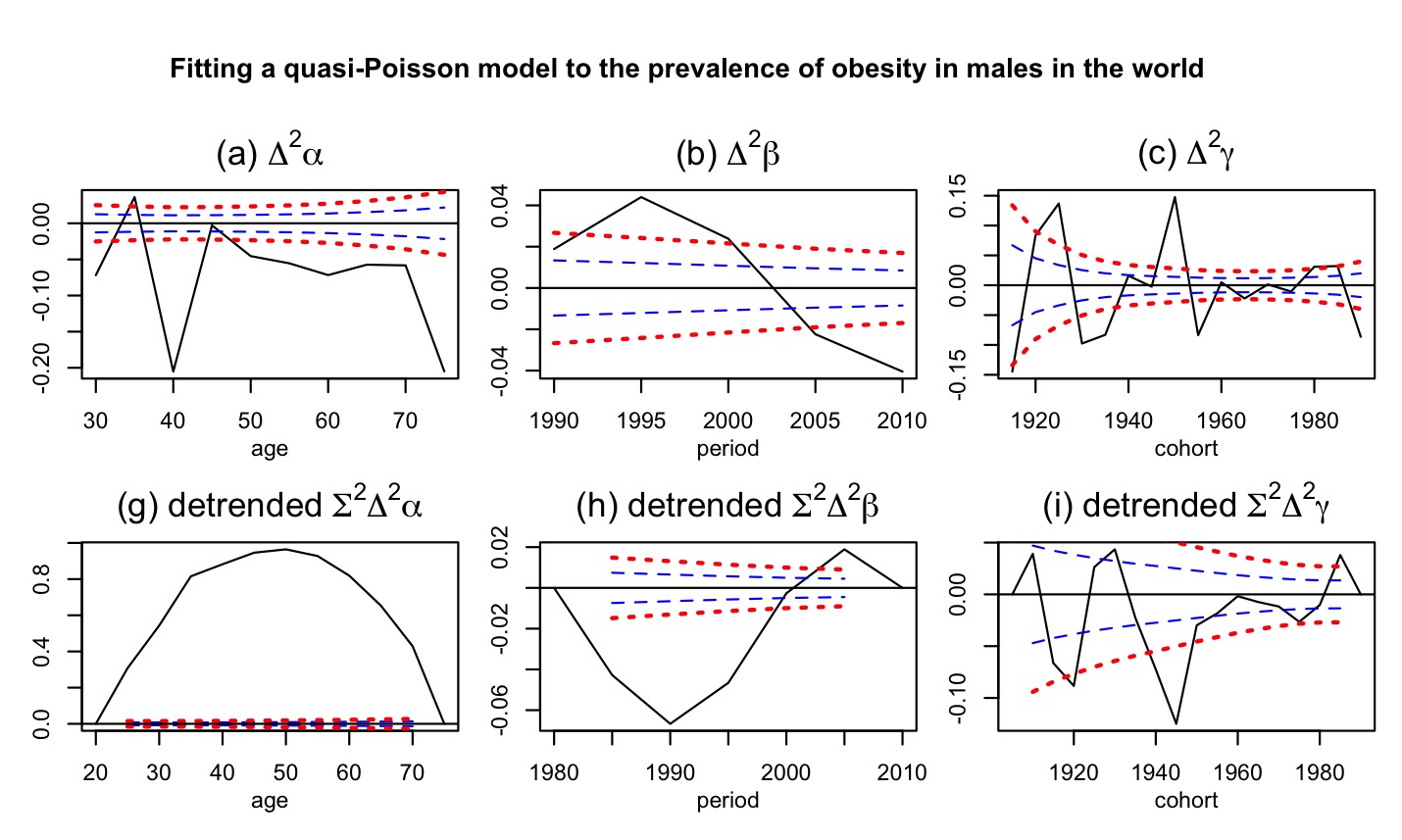


Supplementary Figure 36. Plots of the results of the APC model fit on obesity prevalence in males in the world. A quasi-Poisson model was fitted to the data.


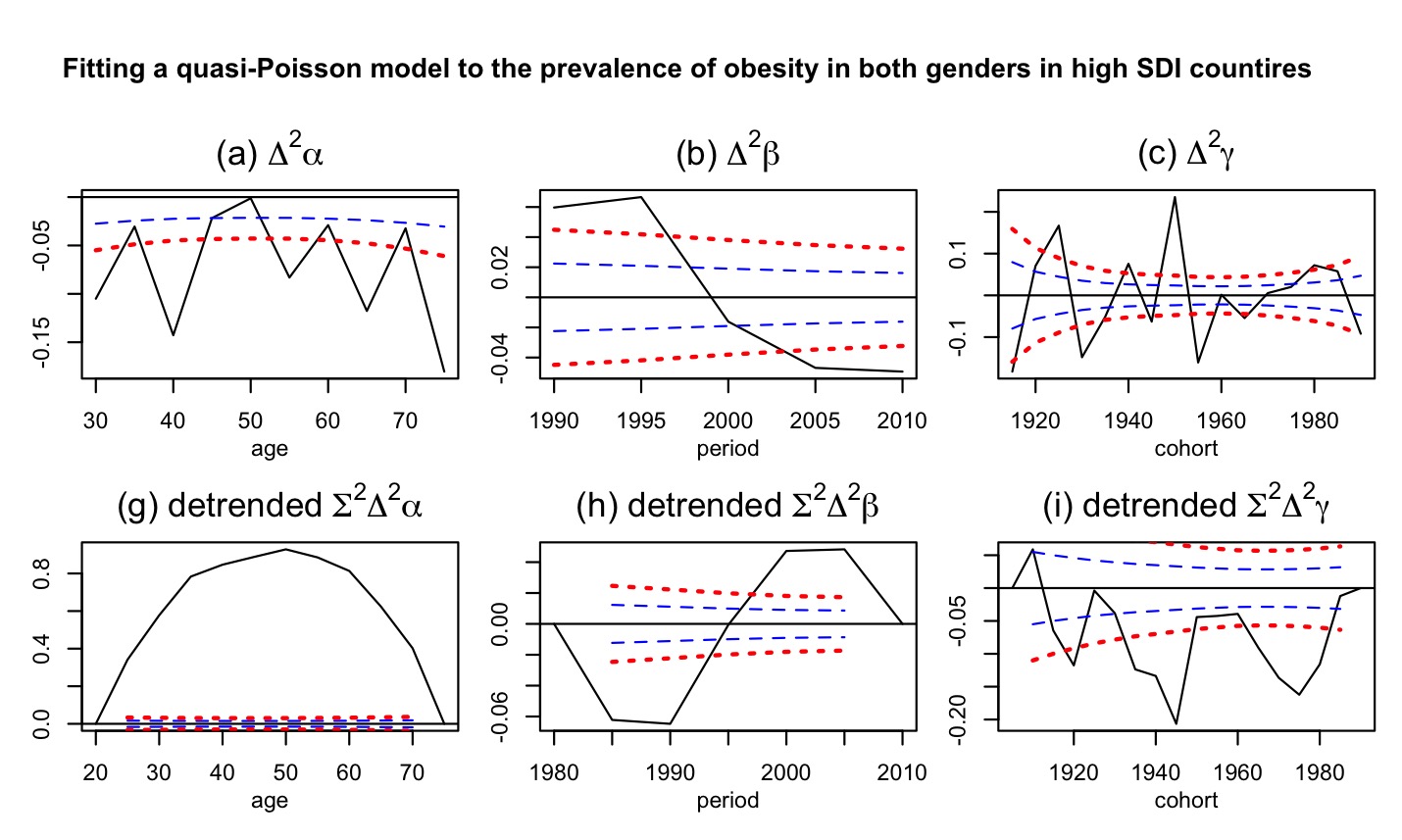


Supplementary Figure 37. Plots of the results of the APC model fit on obesity prevalence in both genders in High SDI countries. A quasi-Poisson model was fitted to the data.


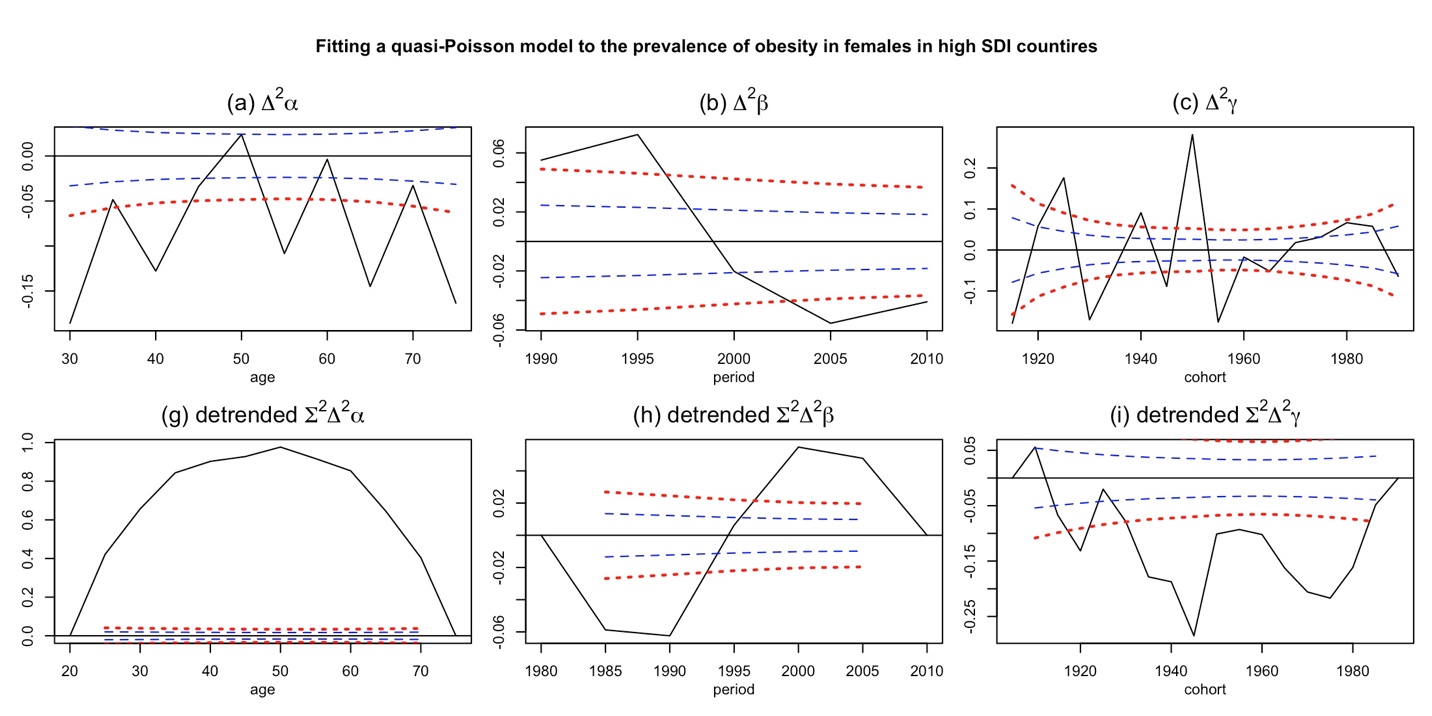


Supplementary Figure 38. Plots of the results of the APC model fit on obesity prevalence in female in the High SDI countries. A quasi-Poisson model was fitted to the data


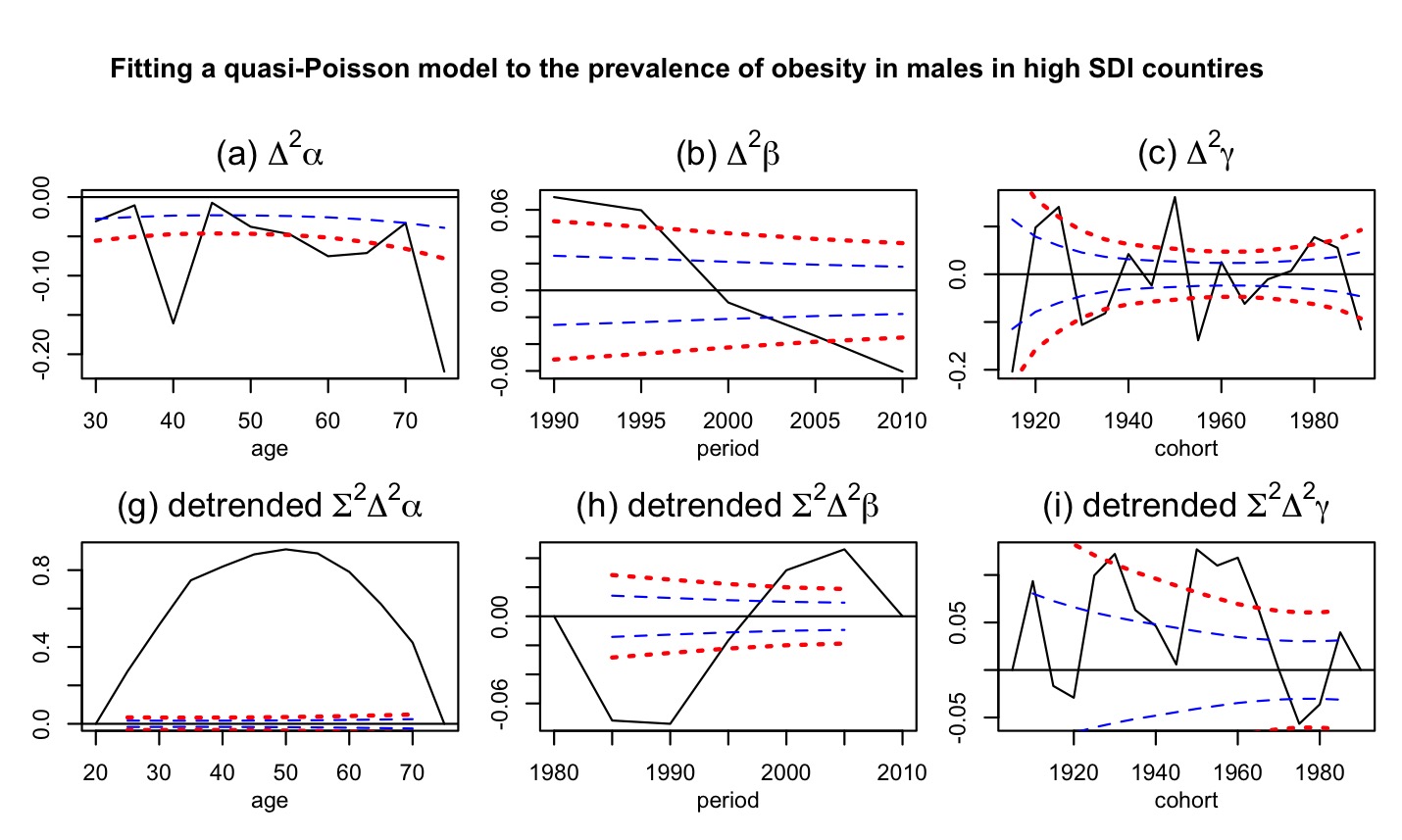


Supplementary Figure 39. Plots of the results of the APC model fit on obesity prevalence in male in the High SDI countries.


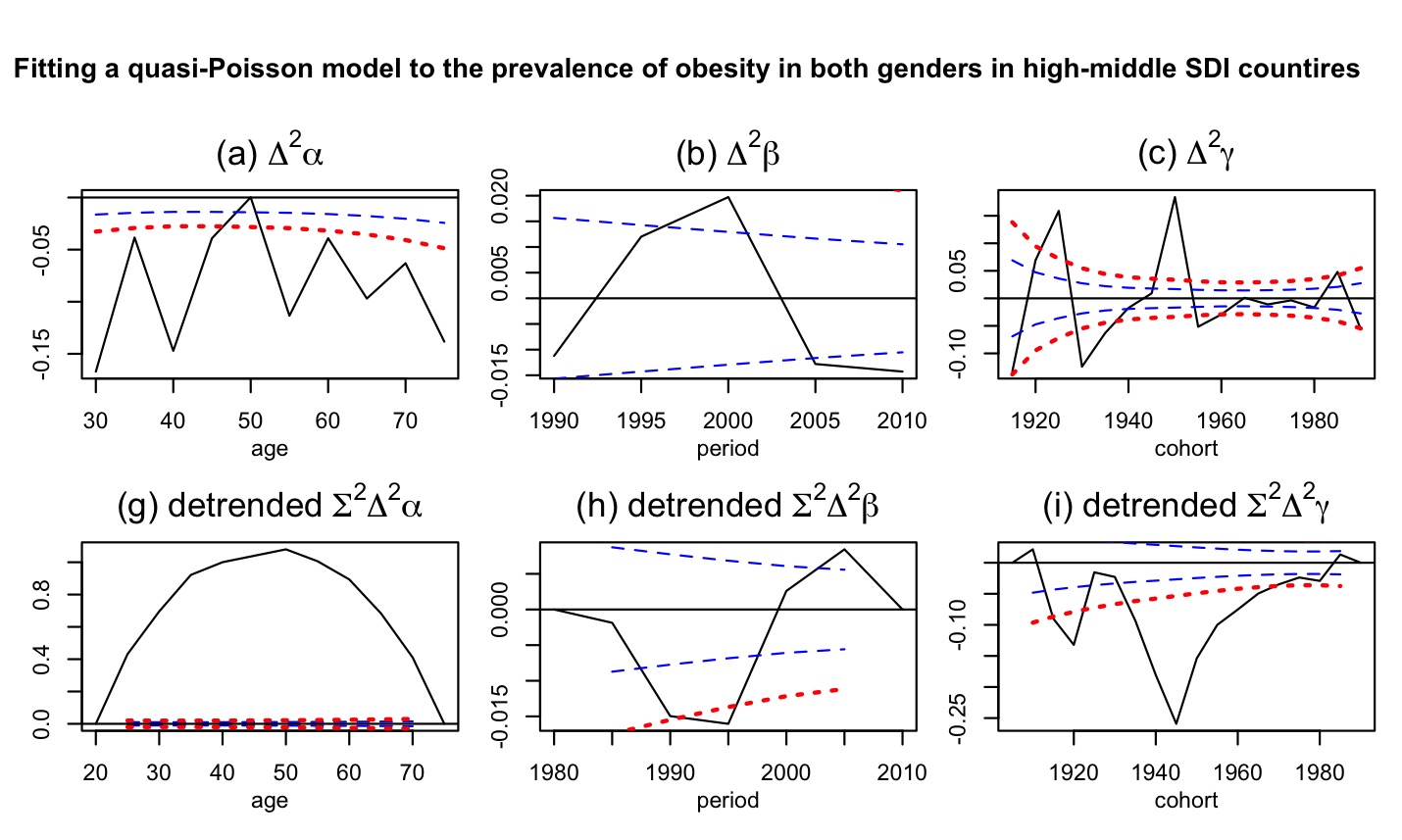


Supplementary Figure 40. Plots of the results of the APC model fit on obesity prevalence in all population in the High-Middle SDI countries.


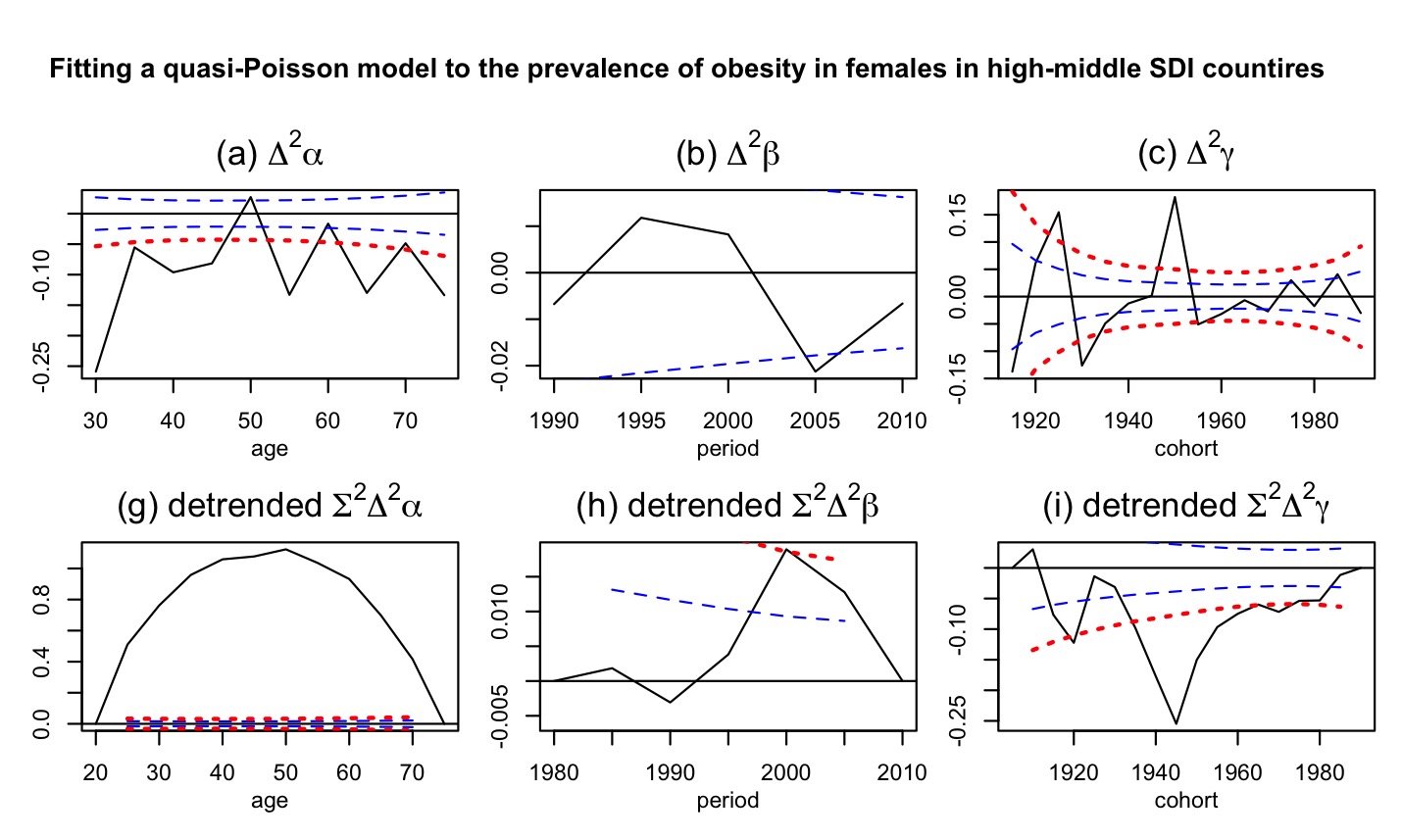


Supplementary Figure 41. Plots of the results of the APC model fit on obesity prevalence in females in the High-Middle SDI countries.


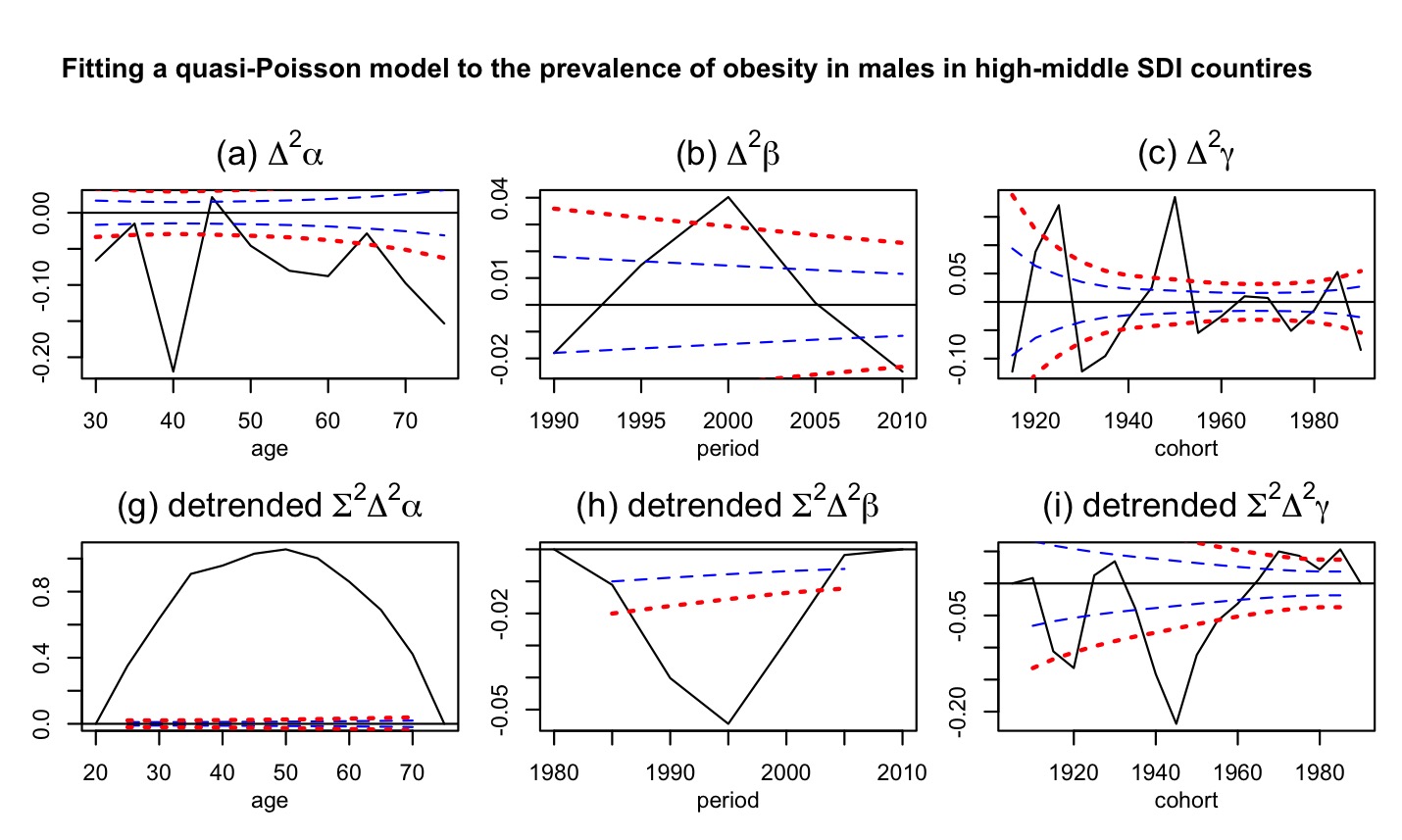


Supplementary Figure 42. Plots of the results of the APC model fit on obesity prevalence in males in the High-Middle SDI countries


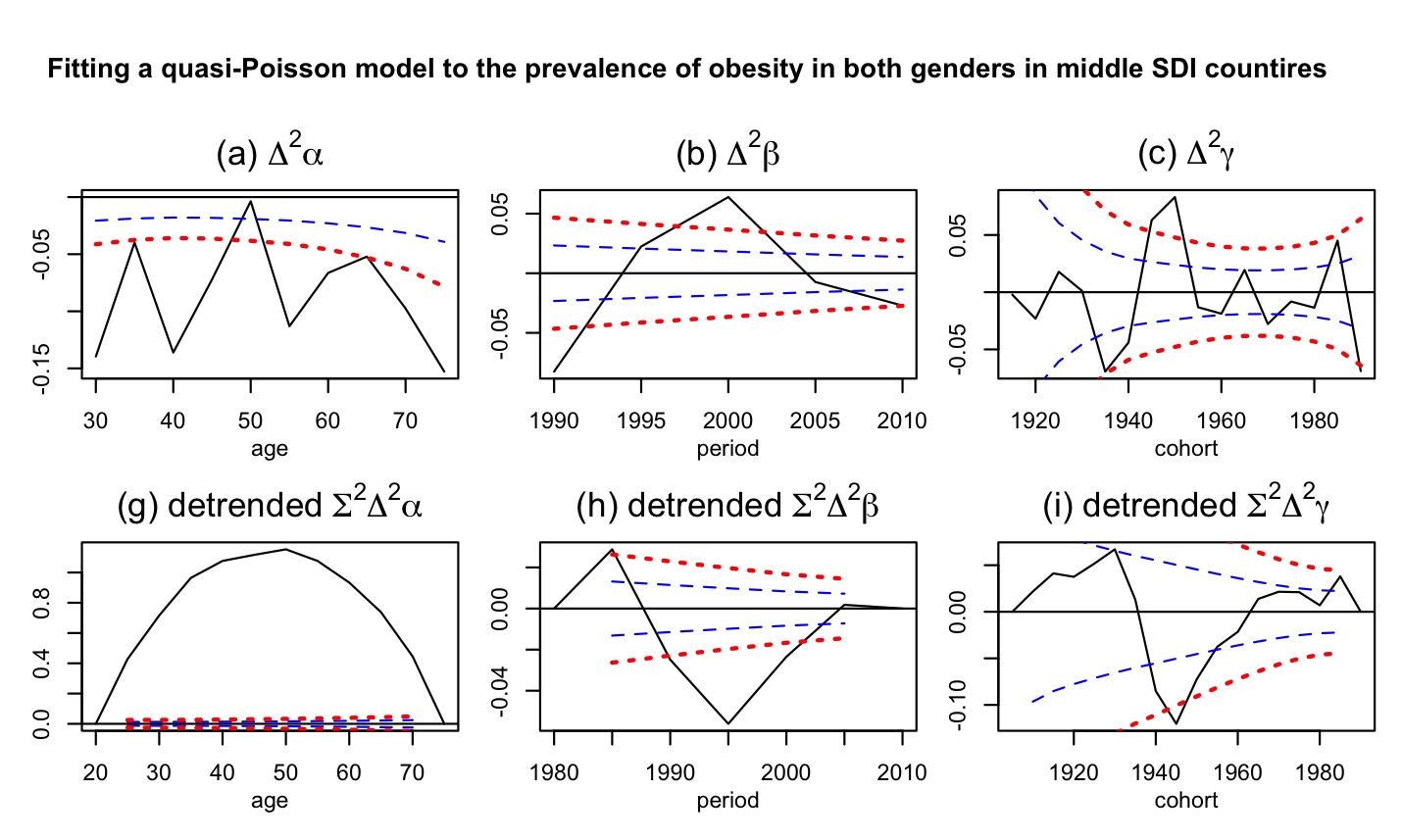


Supplementary Figure 43. Plots of the results of the APC model fit on obesity prevalence in all population in the Middle SDI countries.


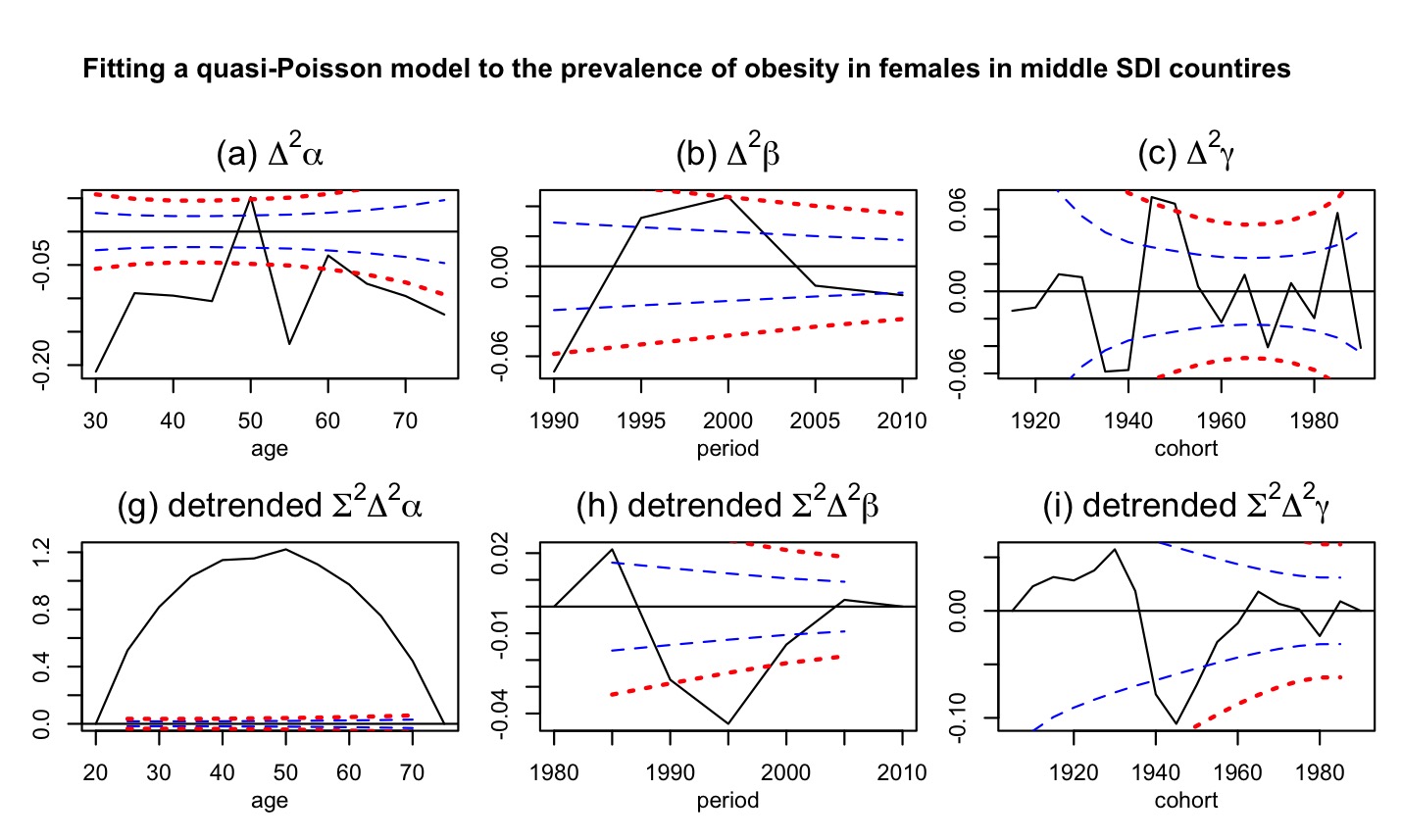


Supplementary Figure 44. Plots of the results of the APC model fit on obesity prevalence in females in the Middle SDI countries.


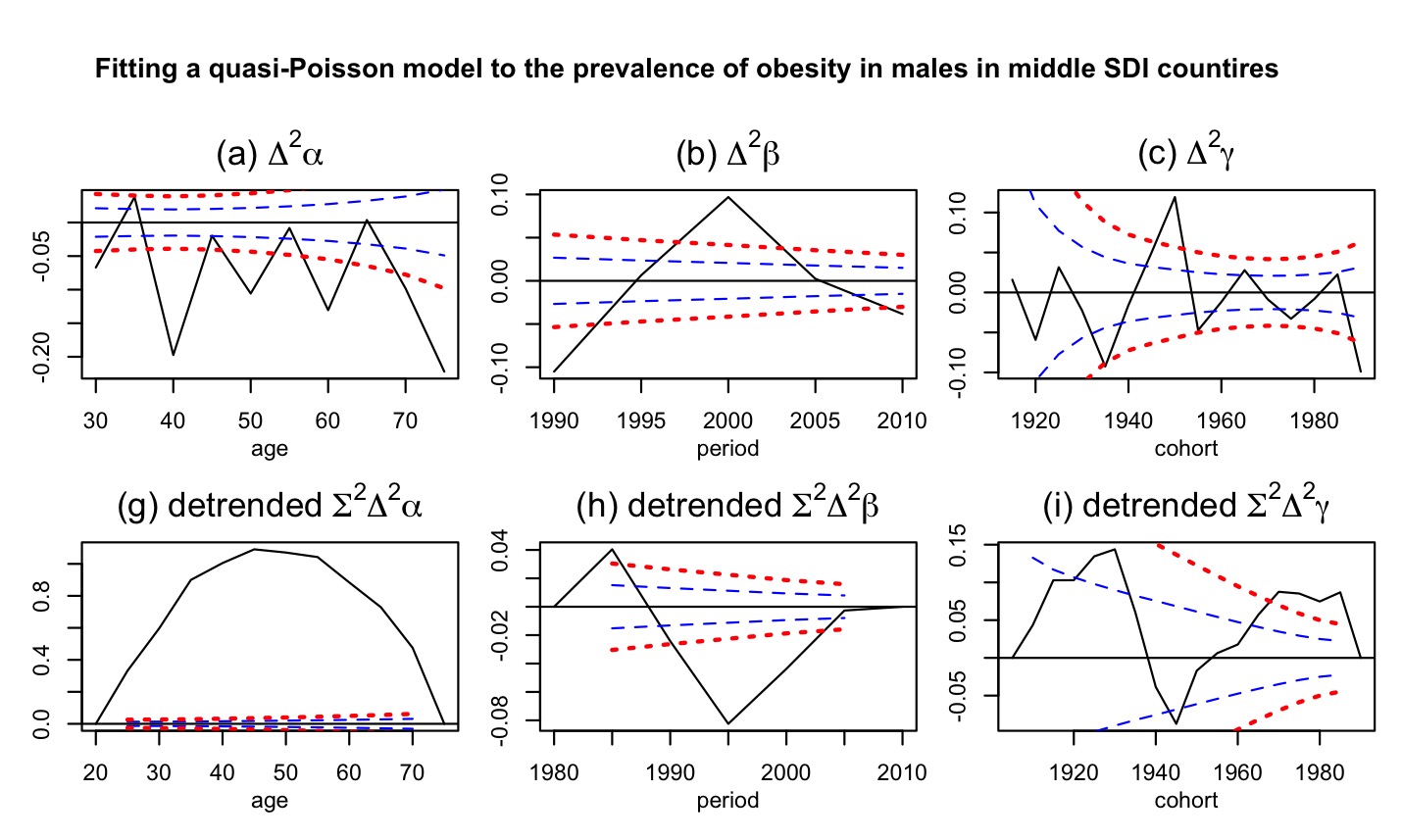


Supplementary Figure 45. Plots of the results of the APC model fit on obesity prevalence in males in the Middle SDI countries.


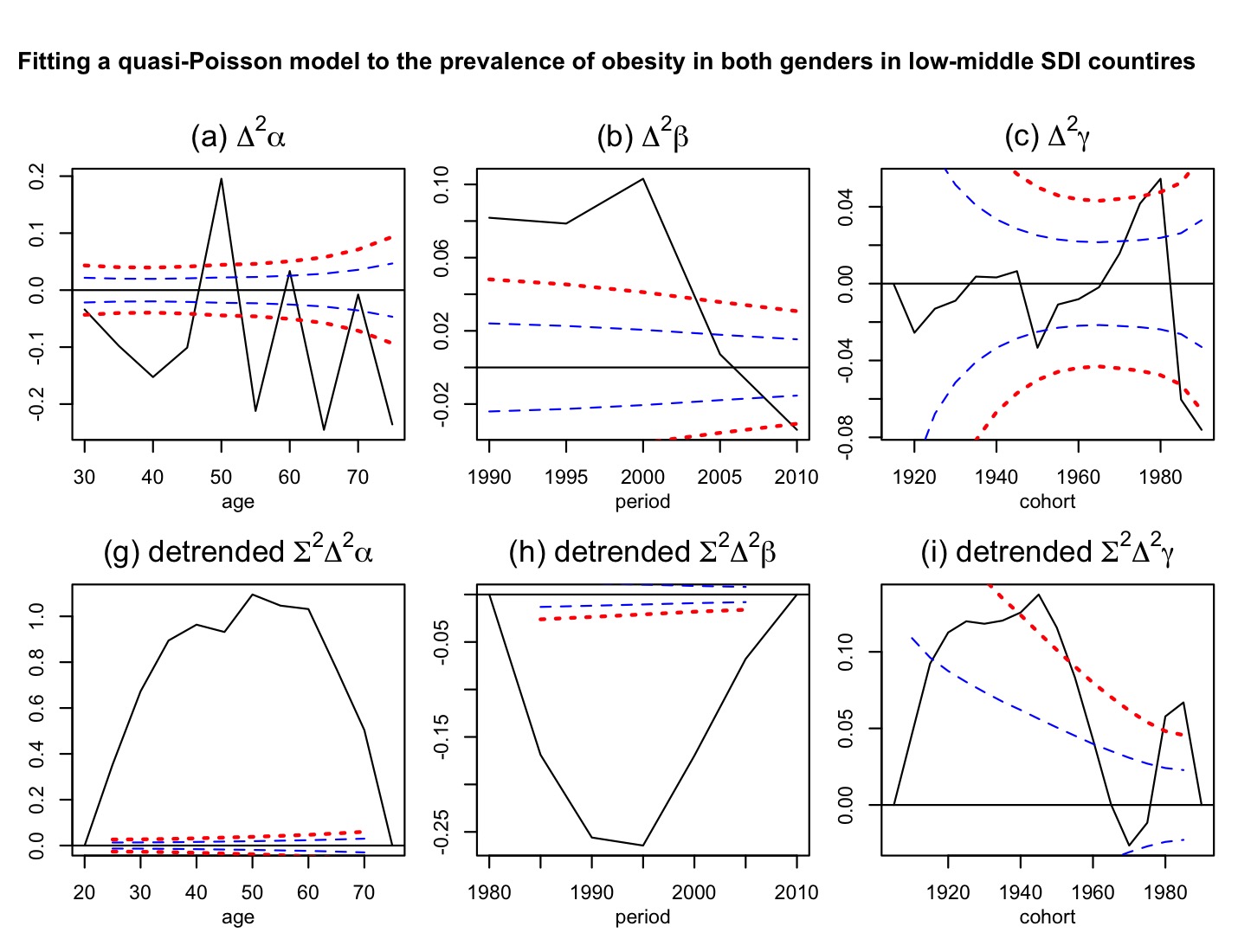


Supplementary Figure 46. Plots of the results of the APC model fit on obesity prevalence in all population in the Low-Middle SDI countries.


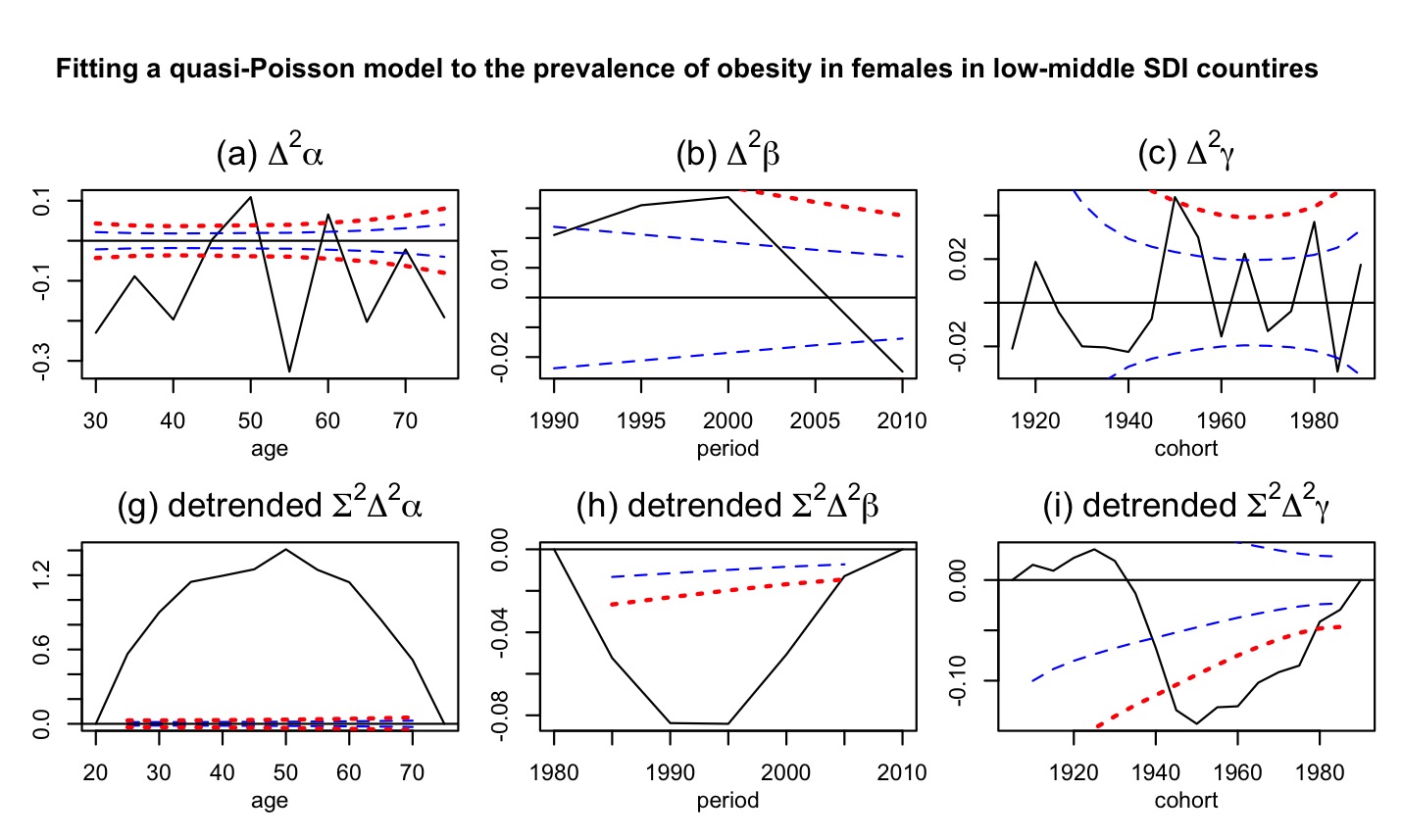


Supplementary Figure 47. Plots of the results of the APC model fit on obesity prevalence in females in the Low-Middle SDI countries.


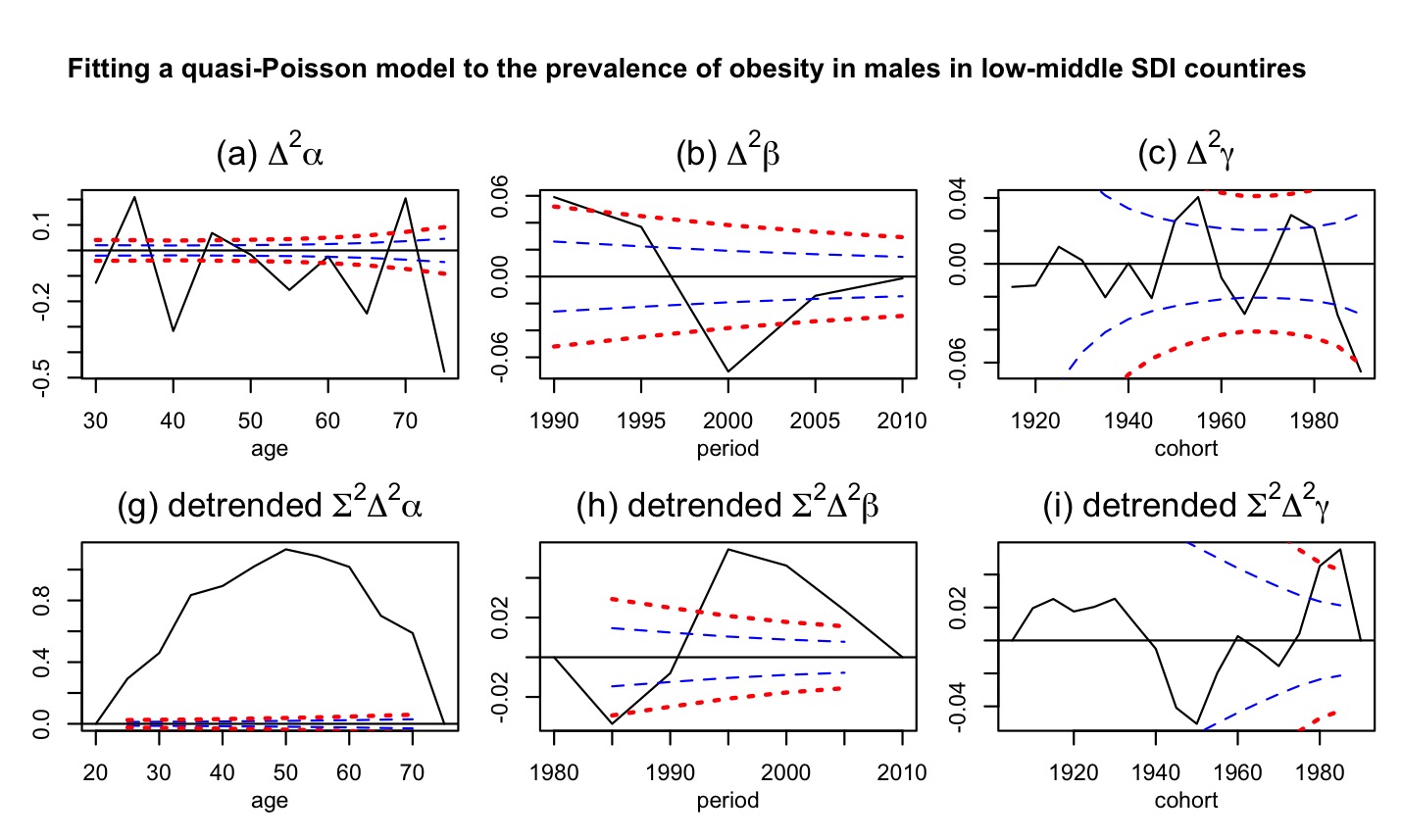


Supplementary Figure 48. Plots of the results of the APC model fit on obesity prevalence in males in the Low-Middle SDI countries.


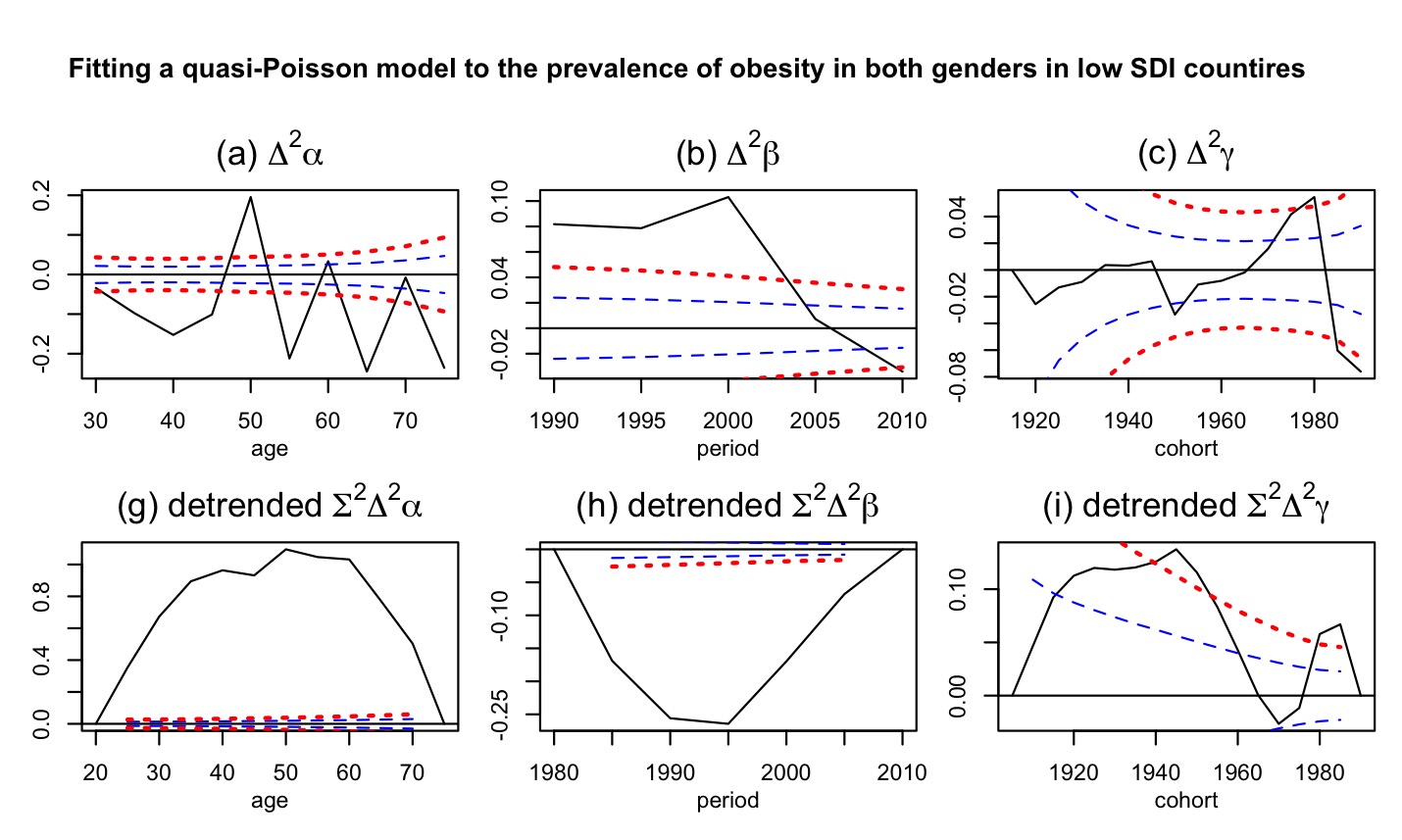


Supplementary Figure 49. Plots of the results of the APC model fit on obesity prevalence in all population in the Low SDI countries.


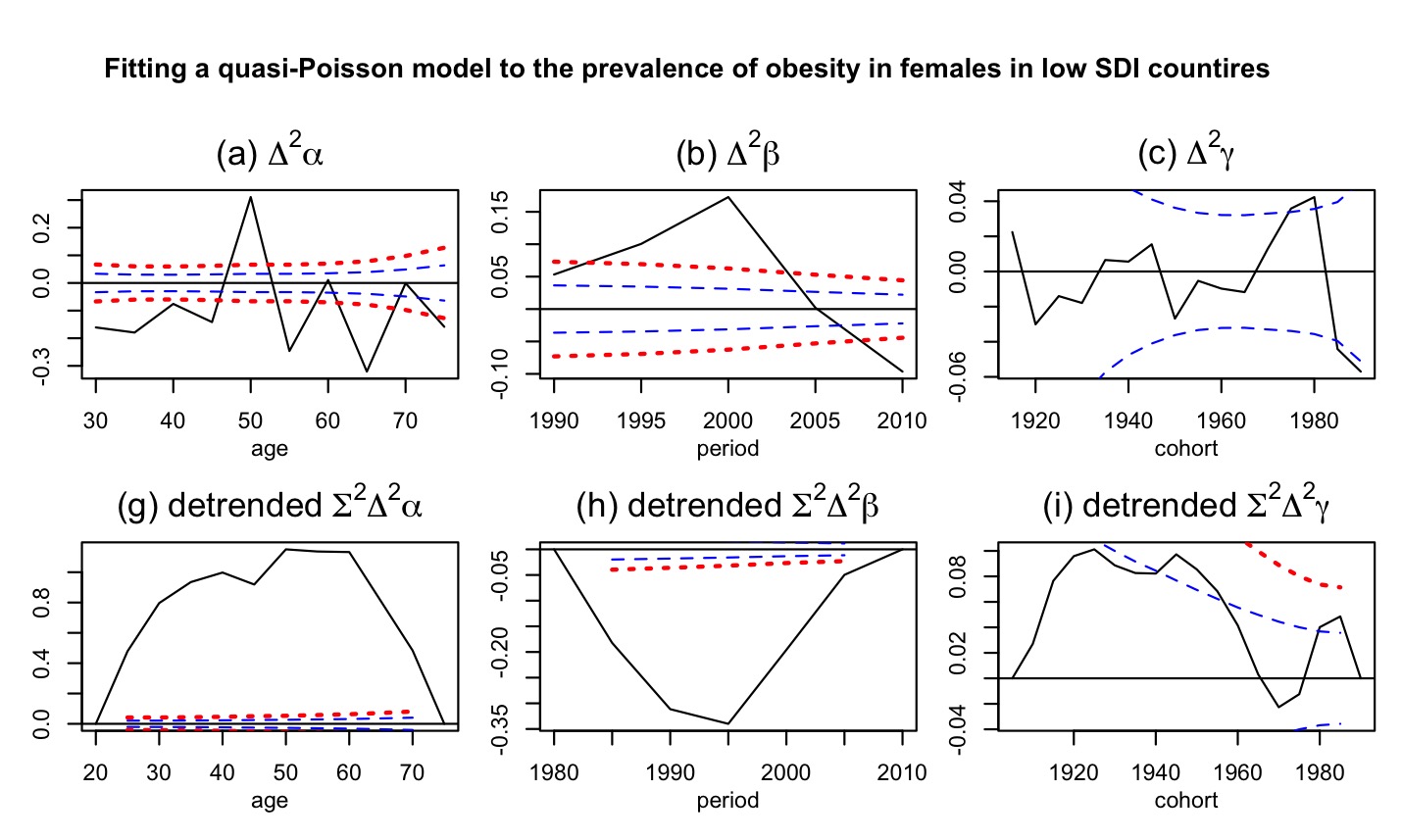


Supplementary Figure 50. Plots of the results of the APC model fit on obesity prevalence in females in the Low SDI countries.


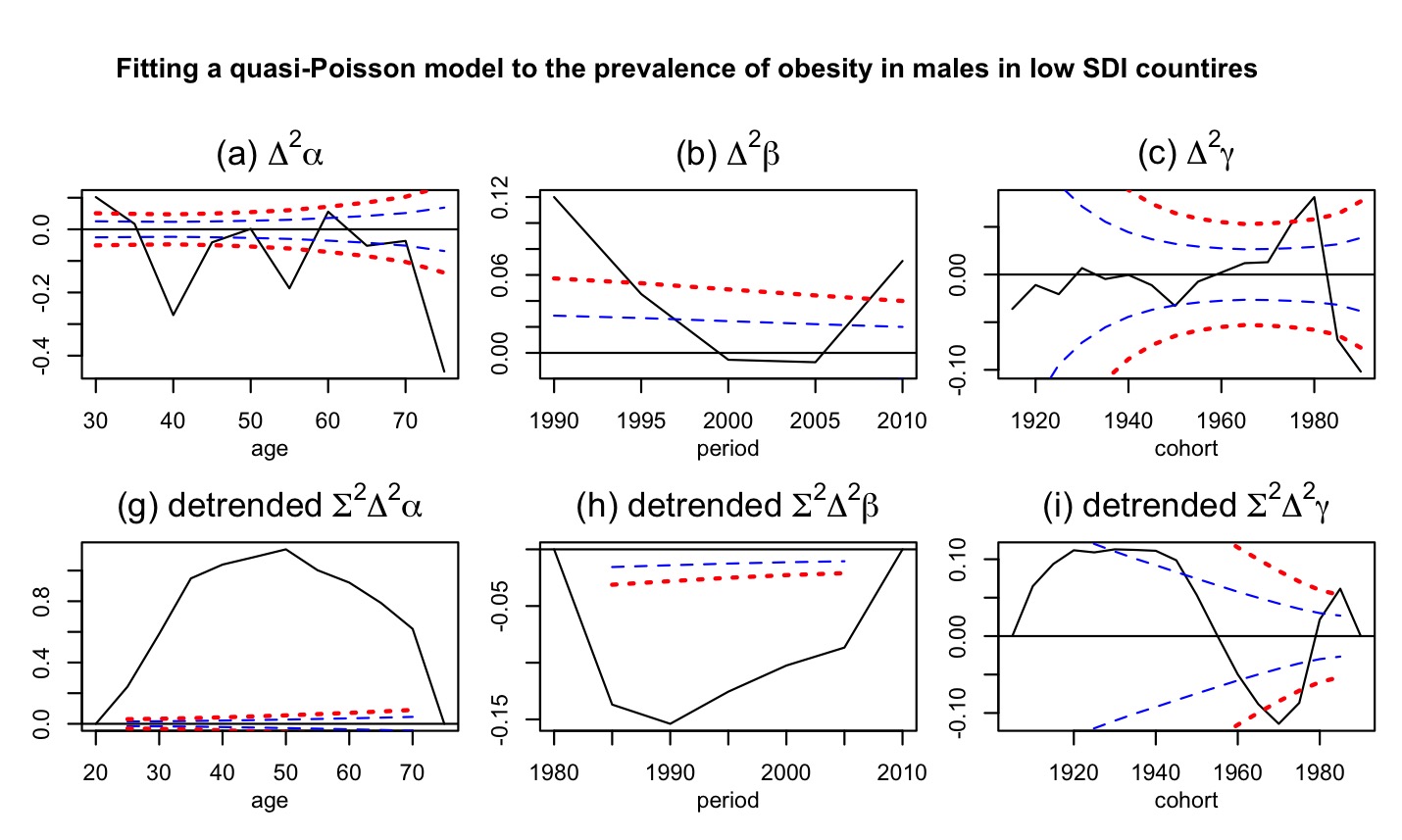


Supplementary Figure 51. Plots of the results of the APC model fit on obesity prevalence in males in the Low SDI countries.


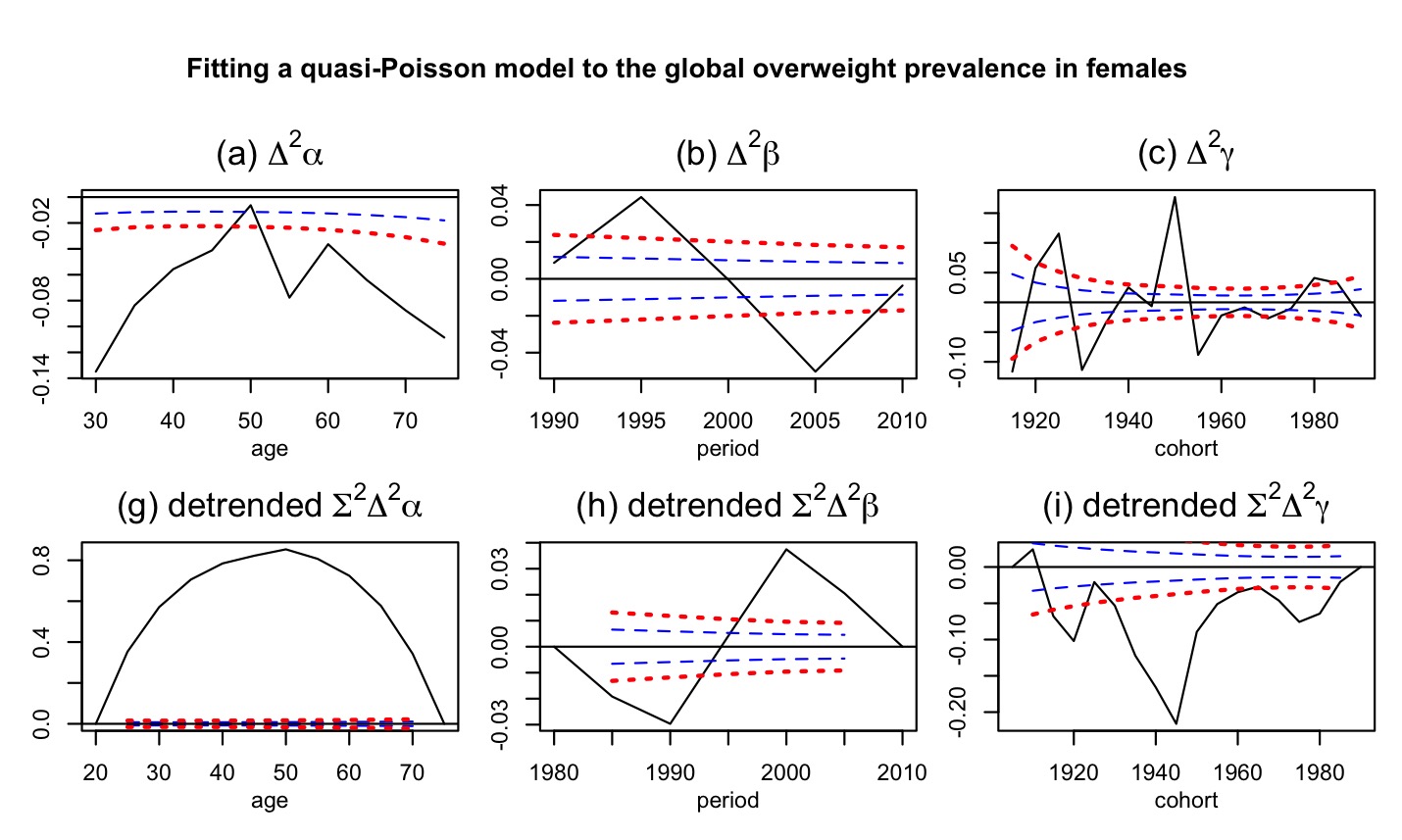


Supplementary Figure 52. Plots of the results of the APC model fit on overweight prevalence in females in the World.


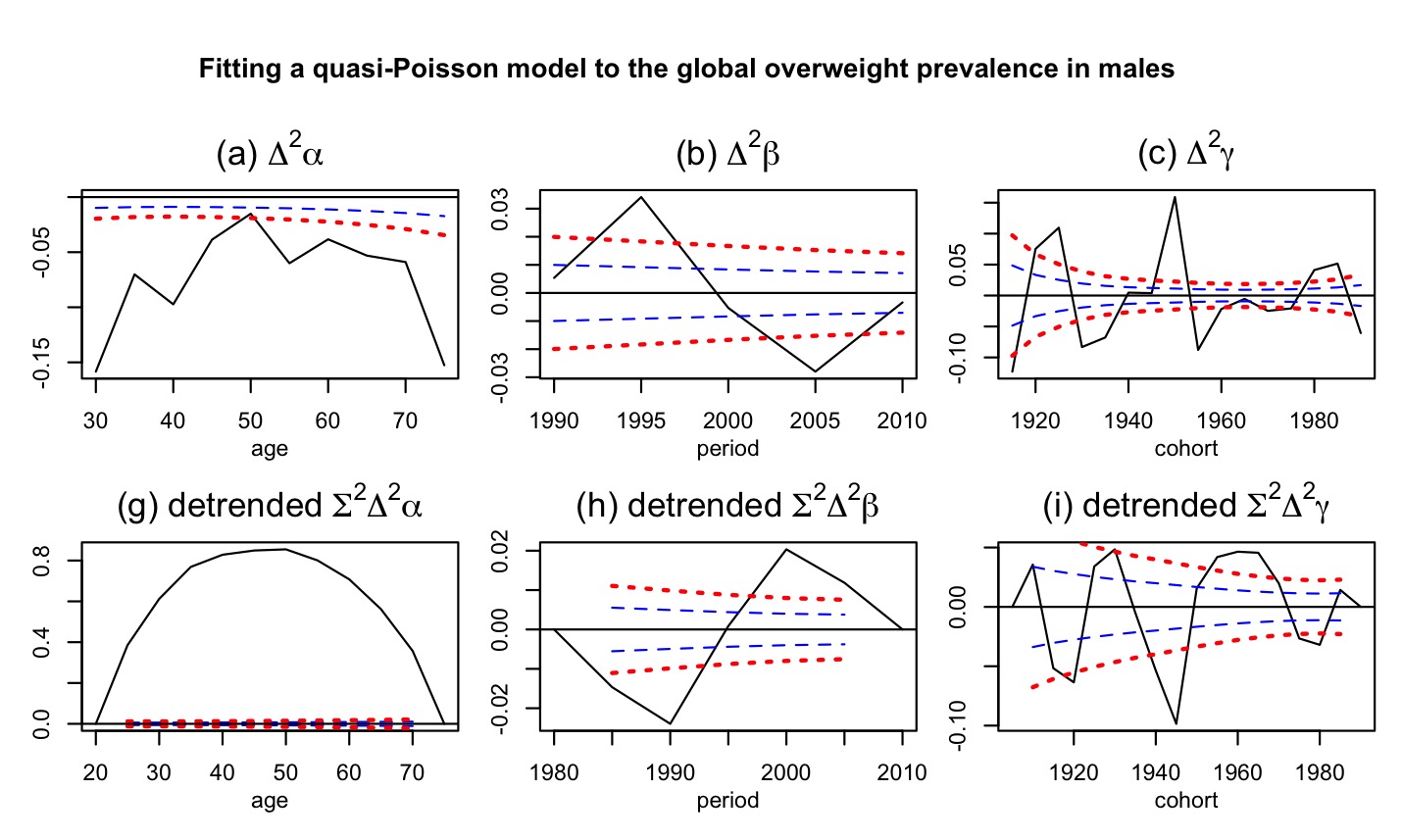


Supplementary Figure 53. Plots of the results of the APC model fit on overweight prevalence in males in the World.


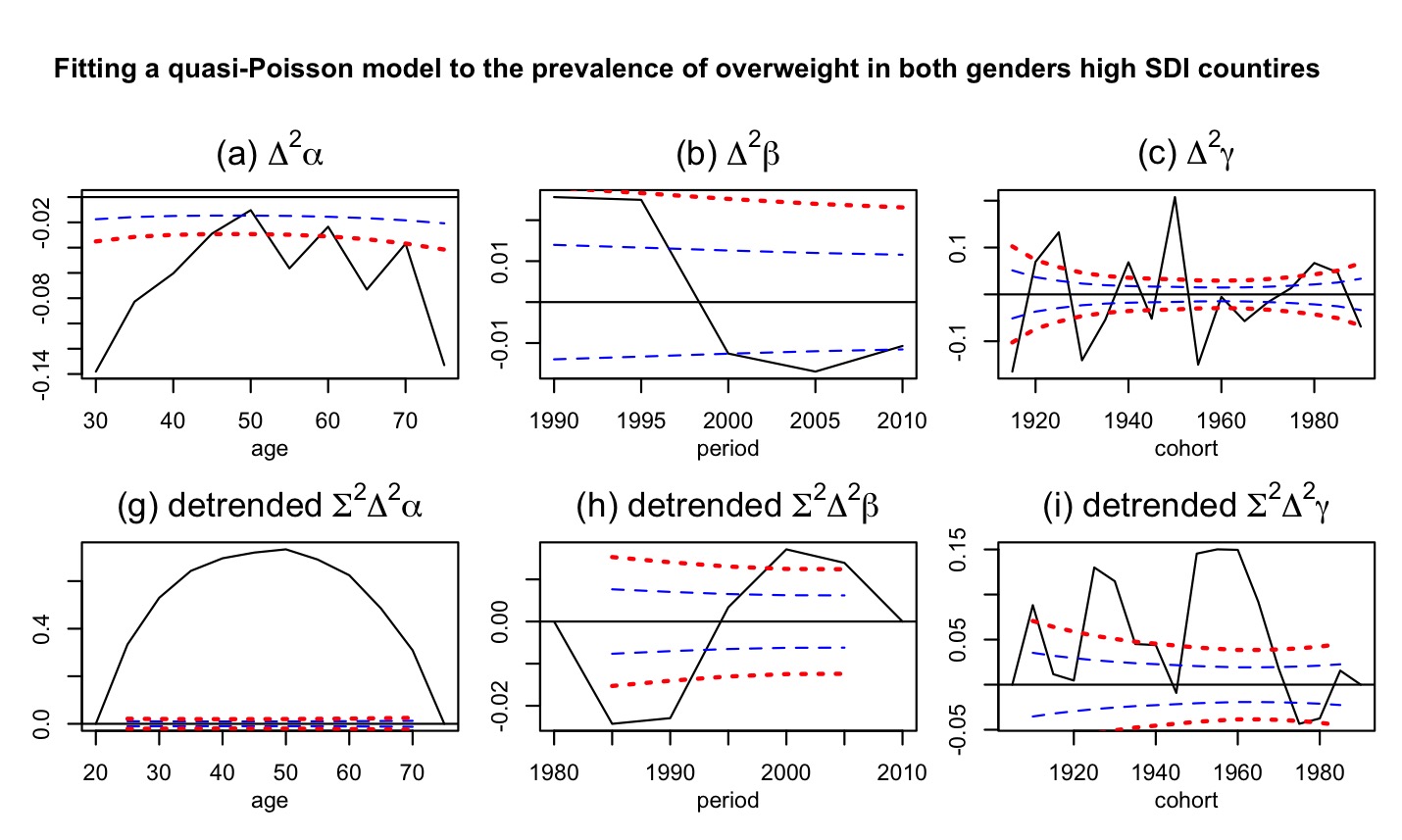


Supplementary Figure 54. Plots of the results of the APC model fit on overweight prevalence in all population in the High SDI countries.


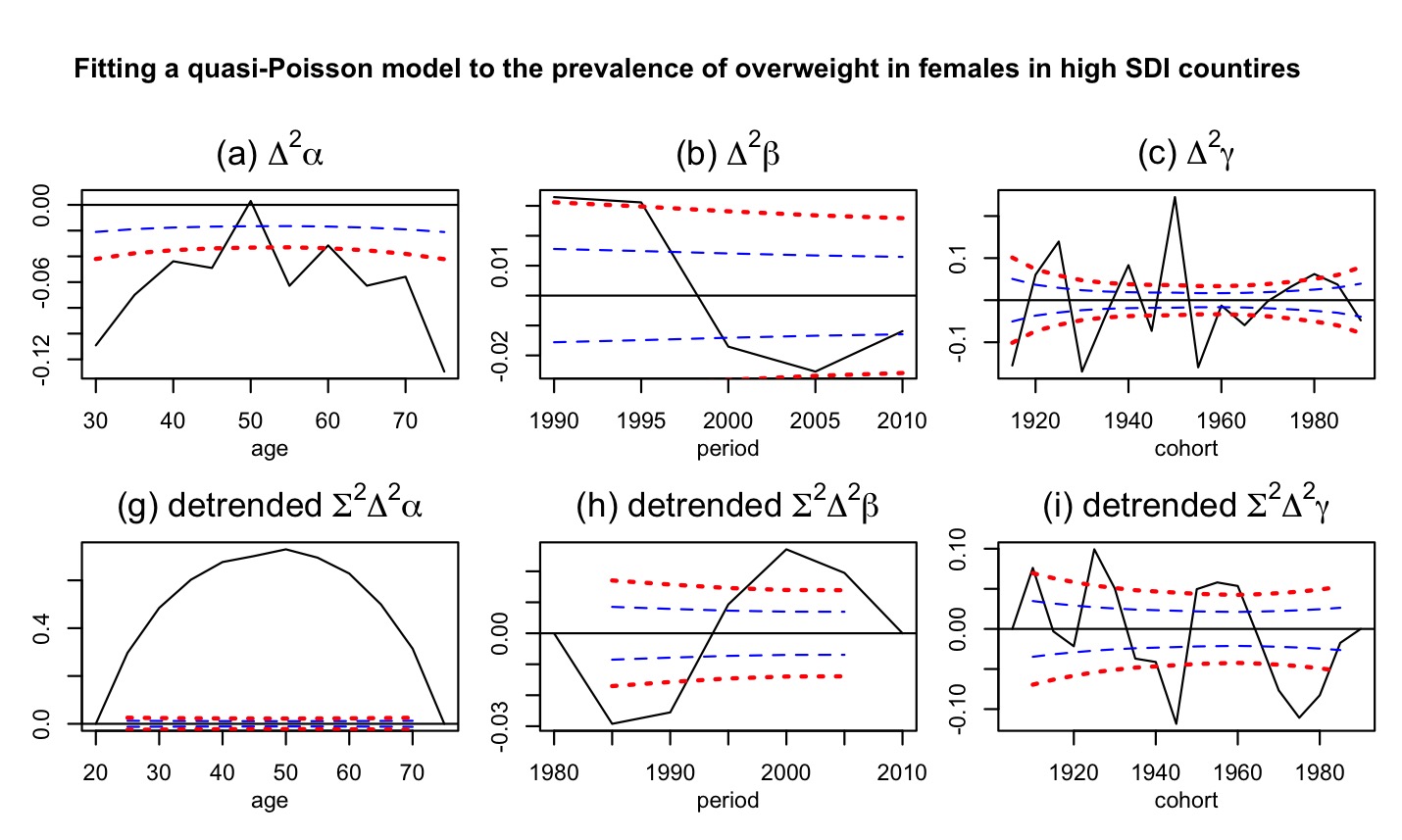


Supplementary Figure 55. Plots of the results of the APC model fit on overweight prevalence in females in the High SDI countries.


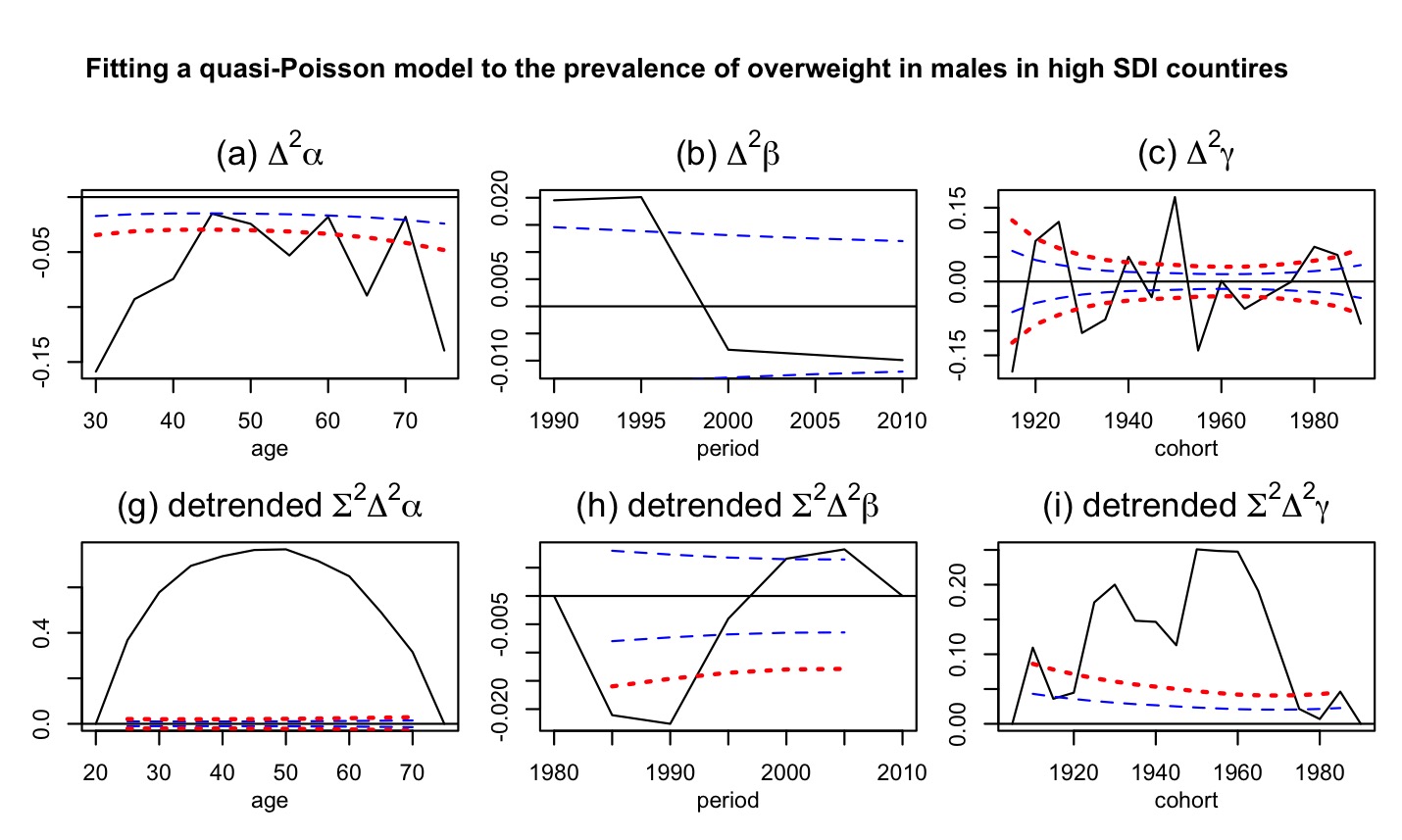


Supplementary Figure 56. Plots of the results of the APC model fit on overweight prevalence in males in the High SDI countries.


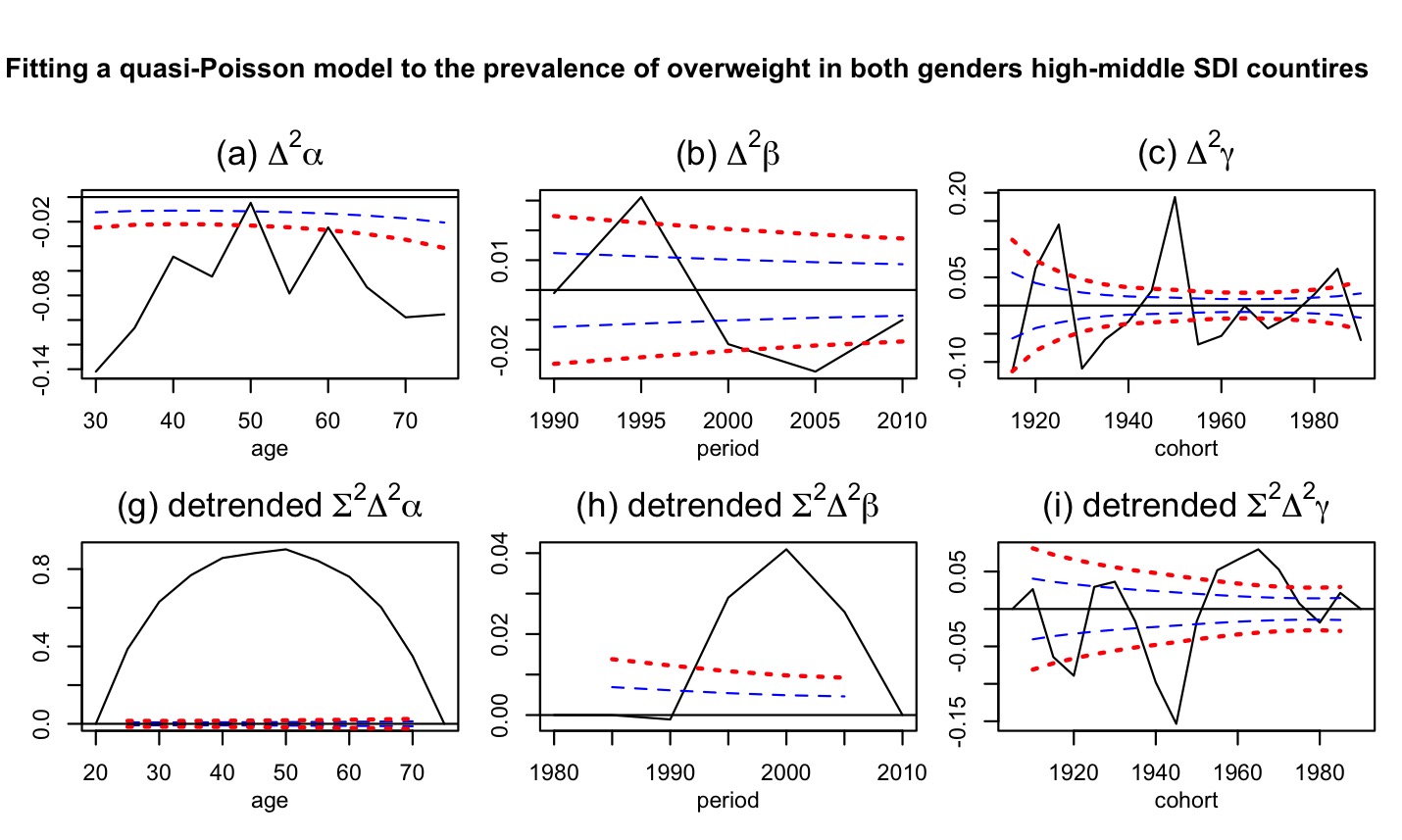


Supplementary Figure 57. Plots of the results of the APC model fit on overweight prevalence in all population in the High-Middle SDI countries.


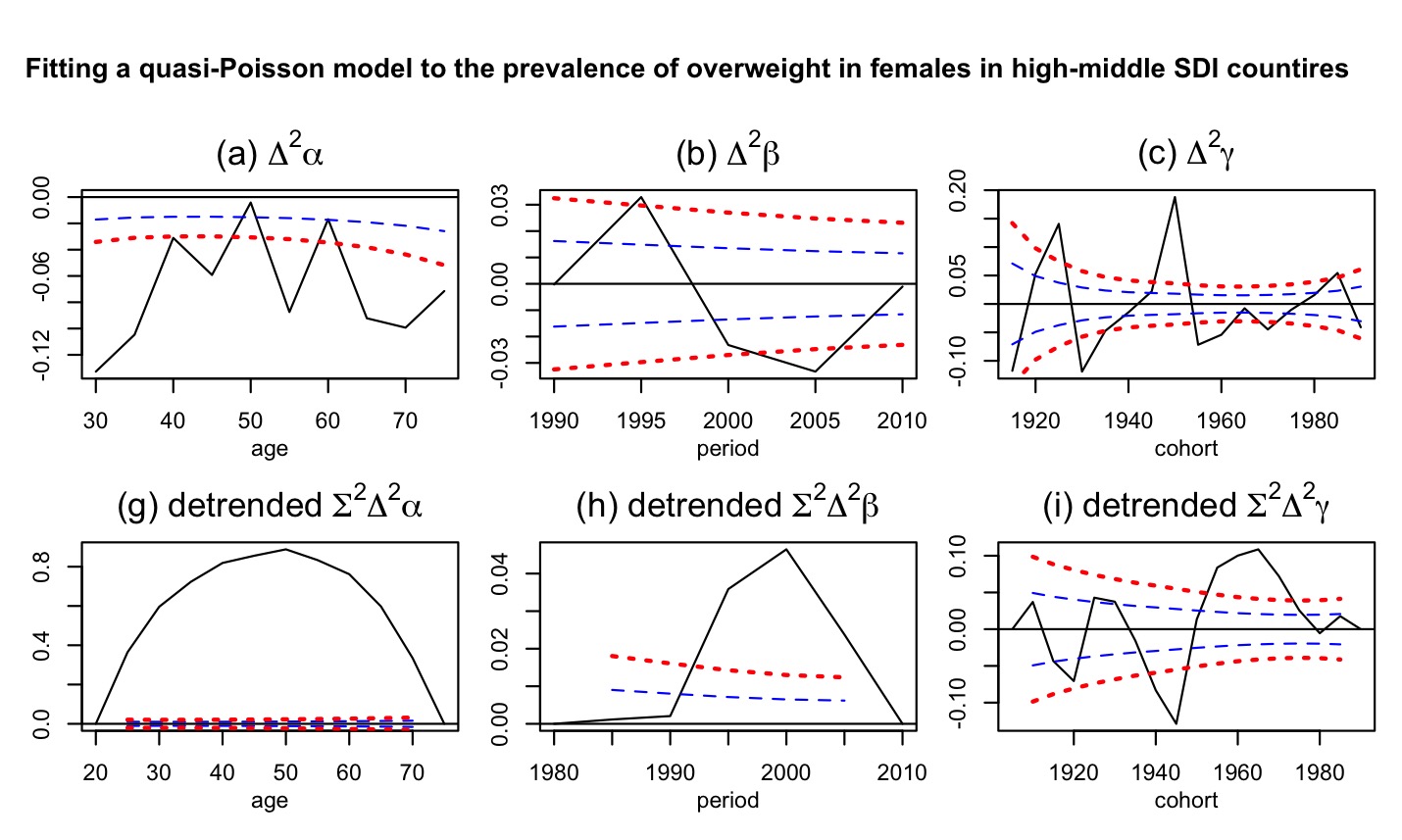


Supplementary Figure 58. Plots of the results of the APC model fit on overweight prevalence in females in the High-Middle SDI countries


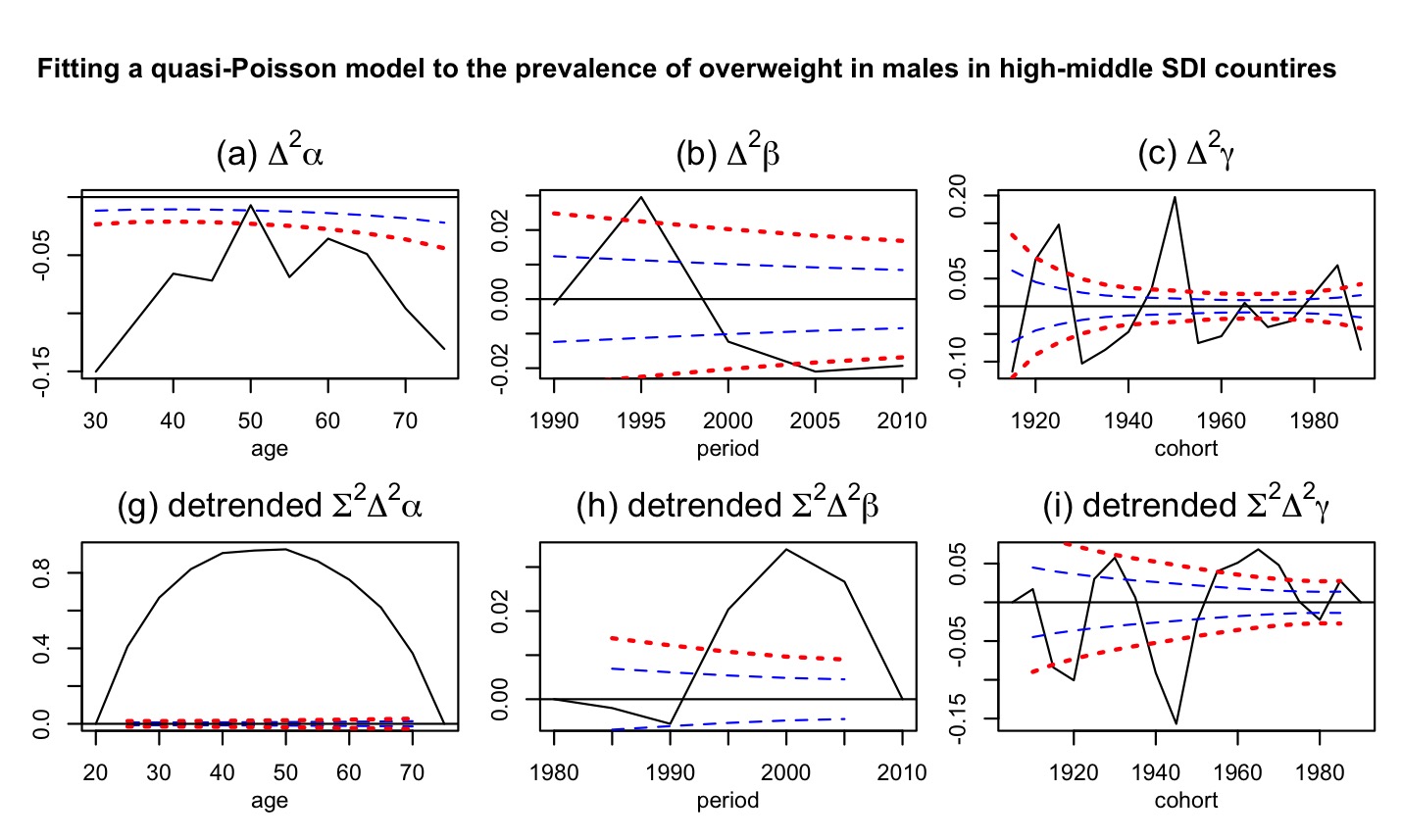


Supplementary Figure 59. Plots of the results of the APC model fit on overweight prevalence in males in the High-Middle SDI countries


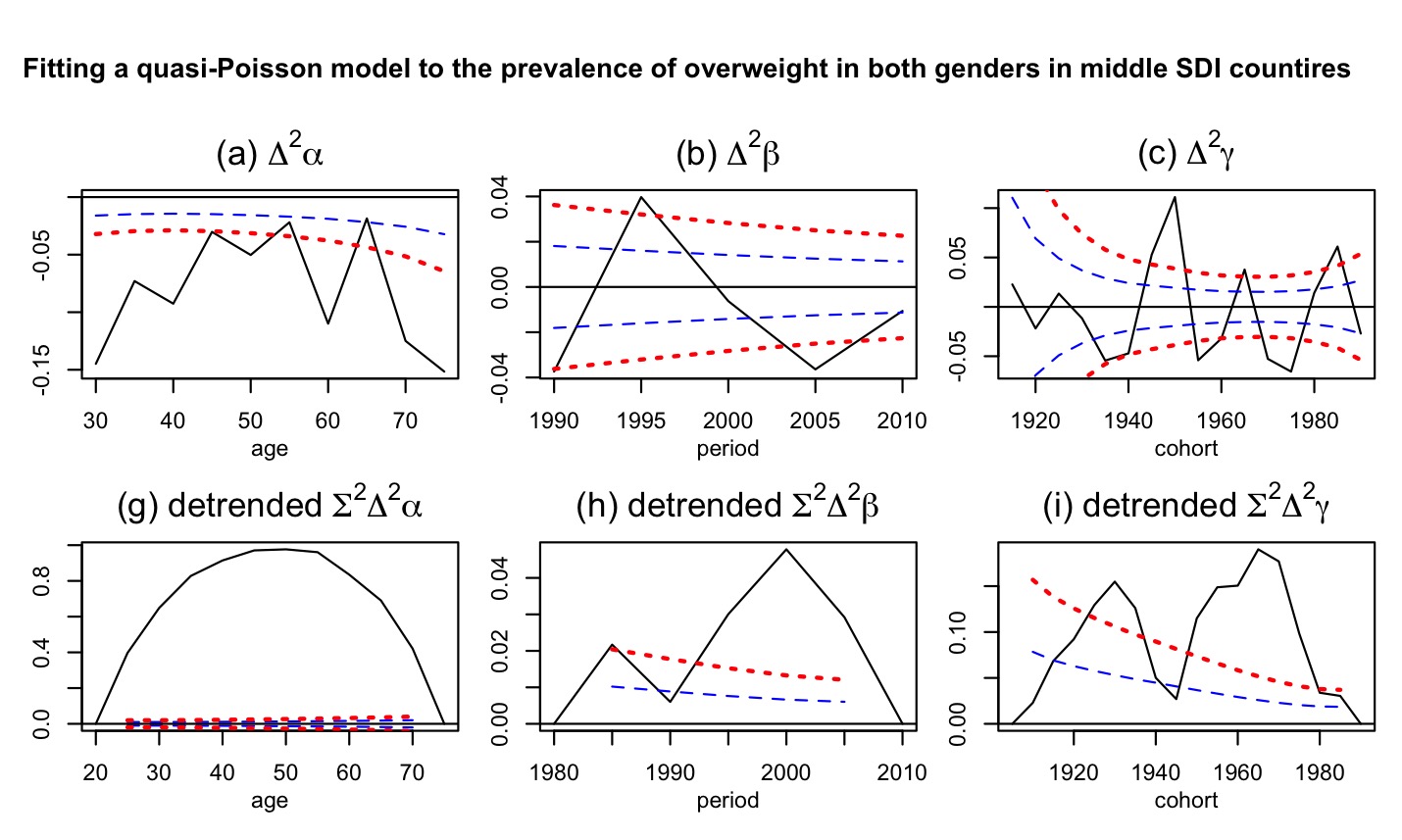


Supplementary Figure 60. Plots of the results of the APC model fit on overweight prevalence in all population in the Middle SDI countries


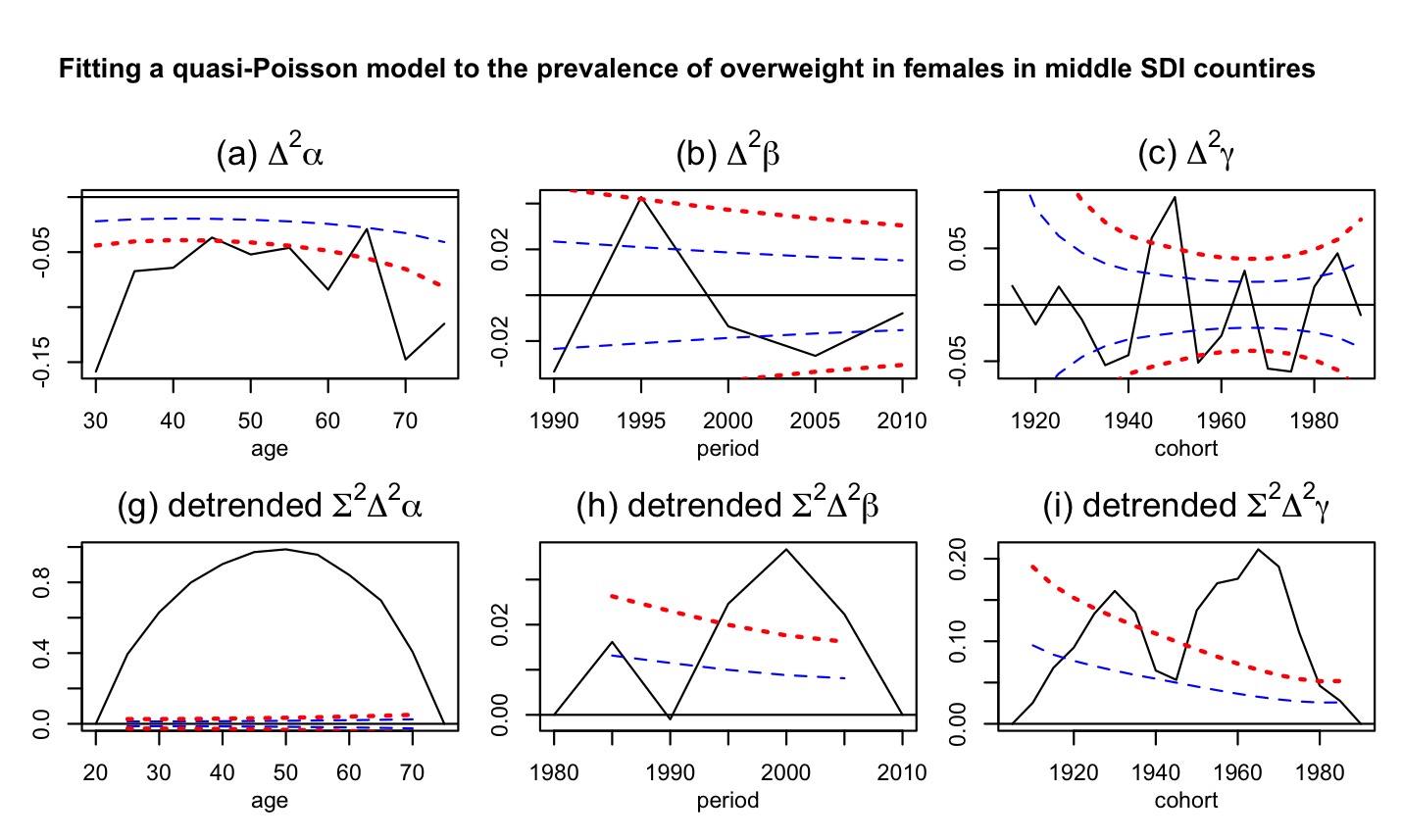


Supplementary Figure 61. Plots of the results of the APC model fit on overweight prevalence in females in the Middle SDI countries.


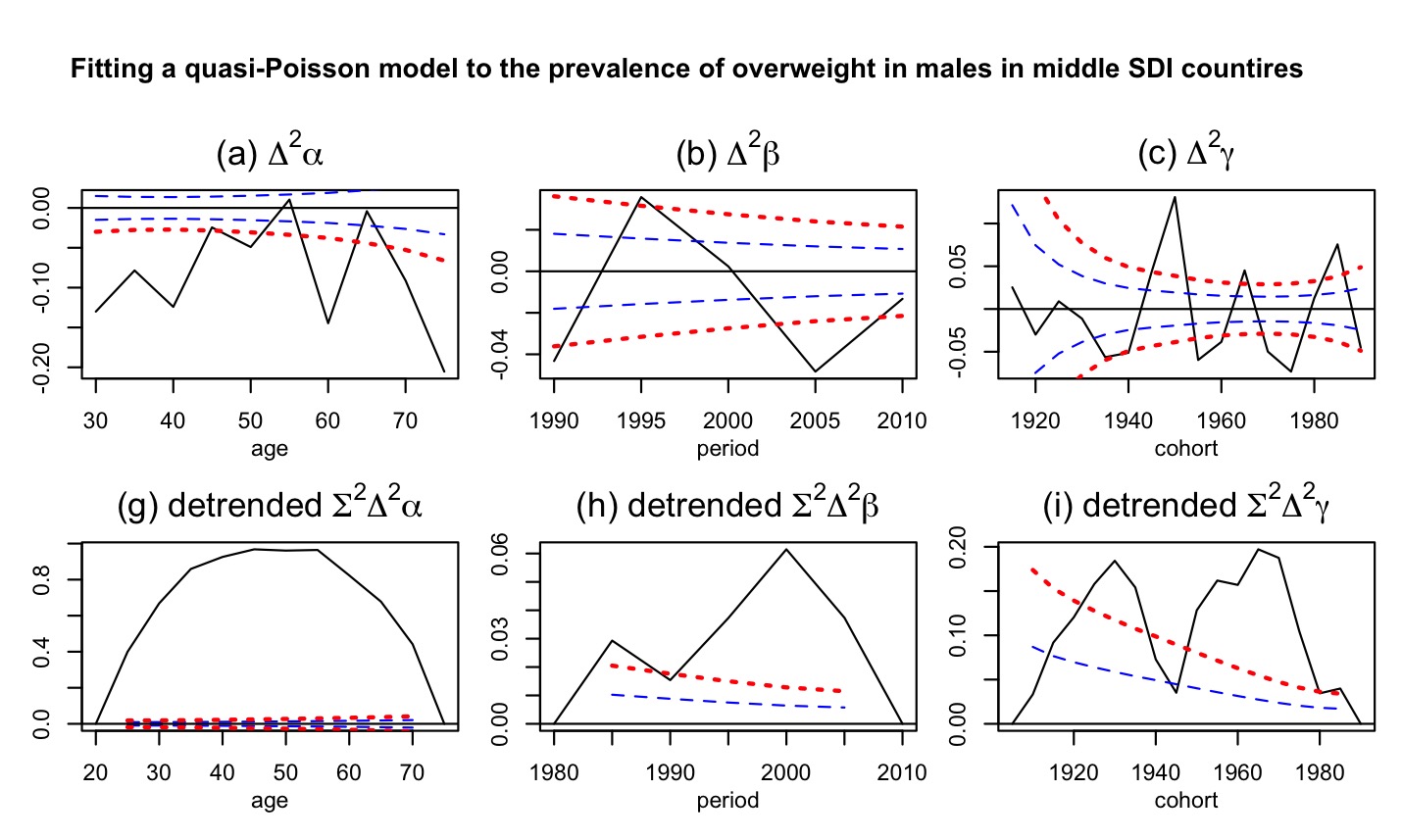


Supplementary Figure 62. Plots of the results of the APC model fit on overweight prevalence in males in the Middle SDI countries.


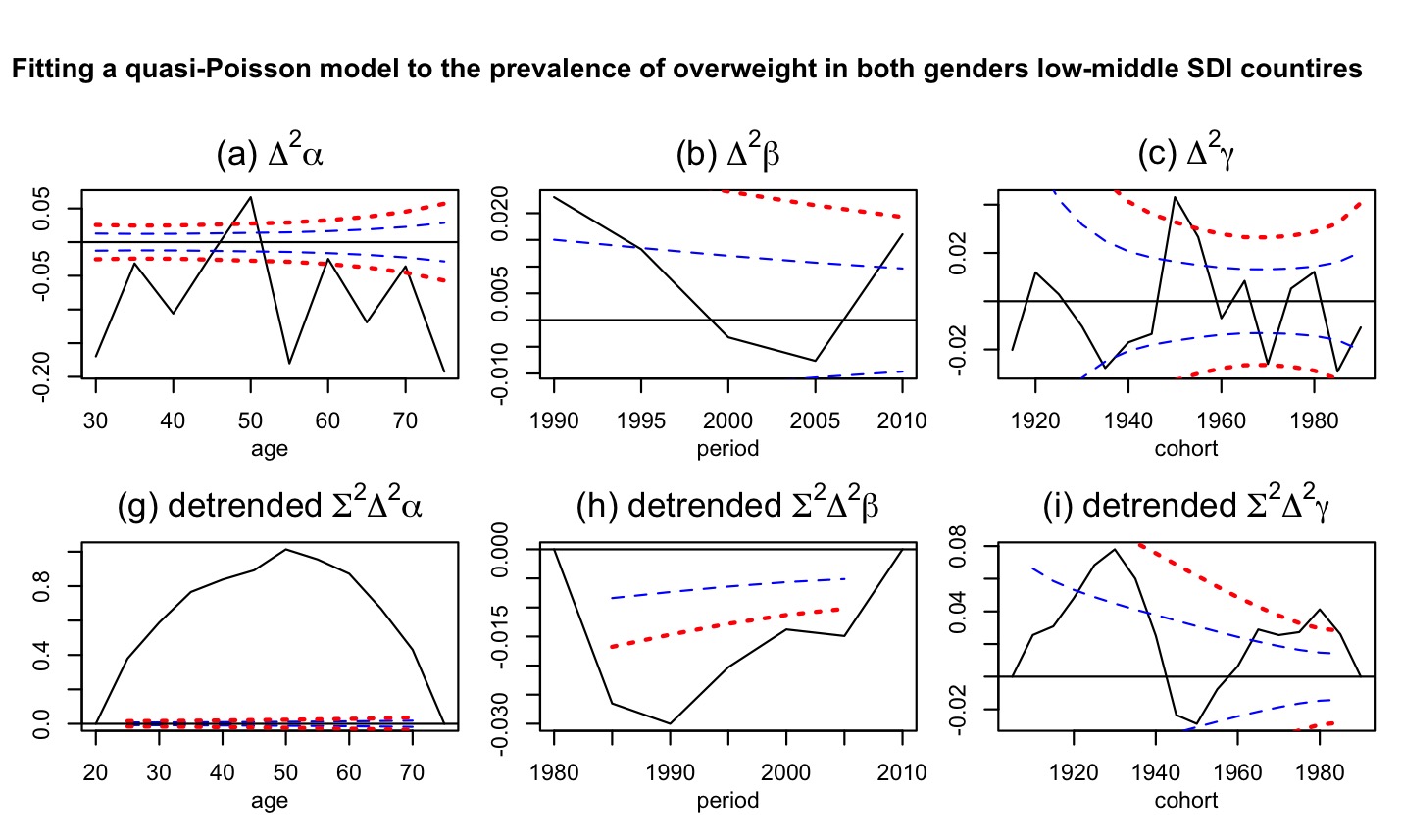


Supplementary Figure 63. Plots of the results of the APC model fit on overweight prevalence in all population in the Low-Middle SDI countries.


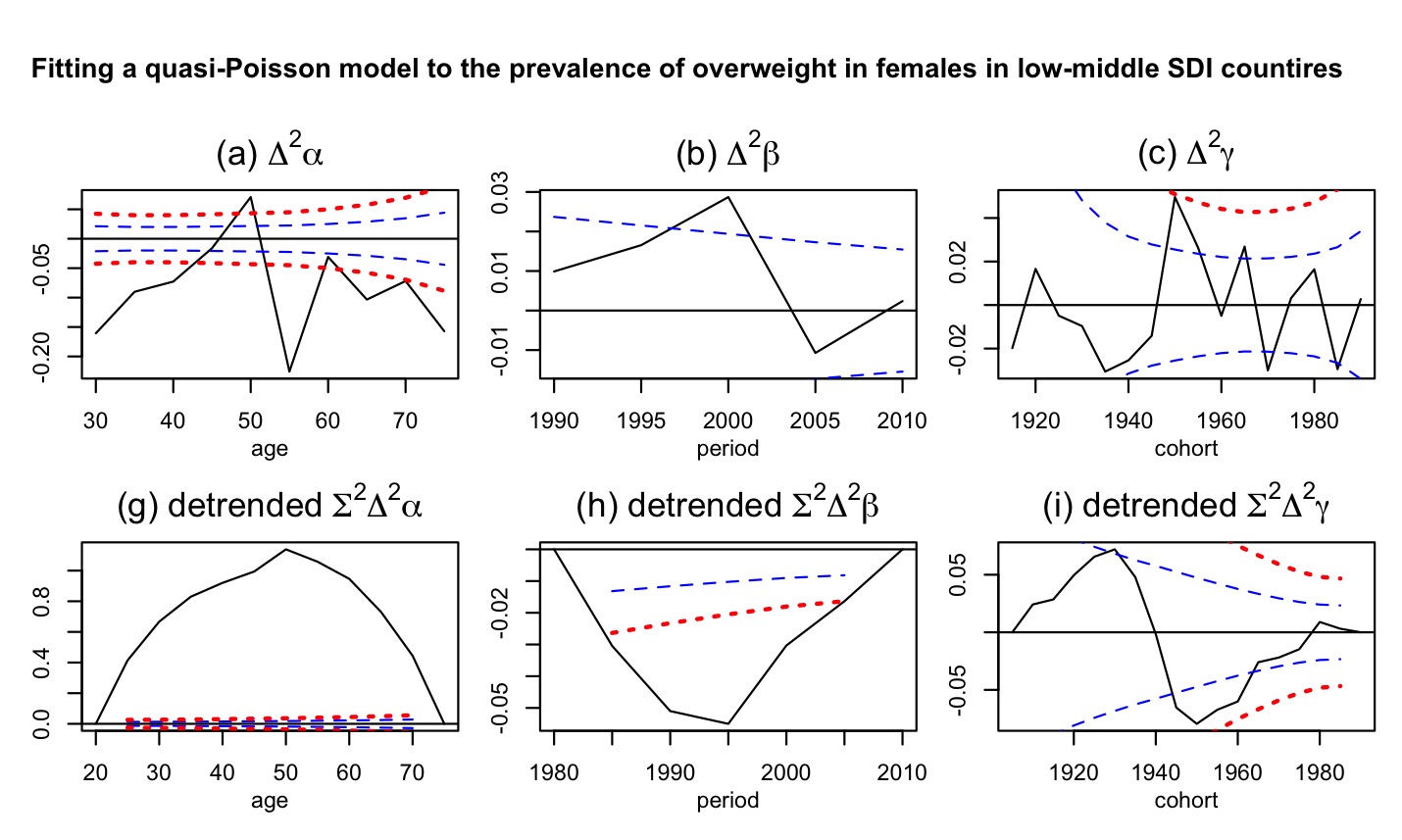


Supplementary Figure 64. Plots of the results of the APC model fit on overweight prevalence in females in the Low-Middle SDI countries.


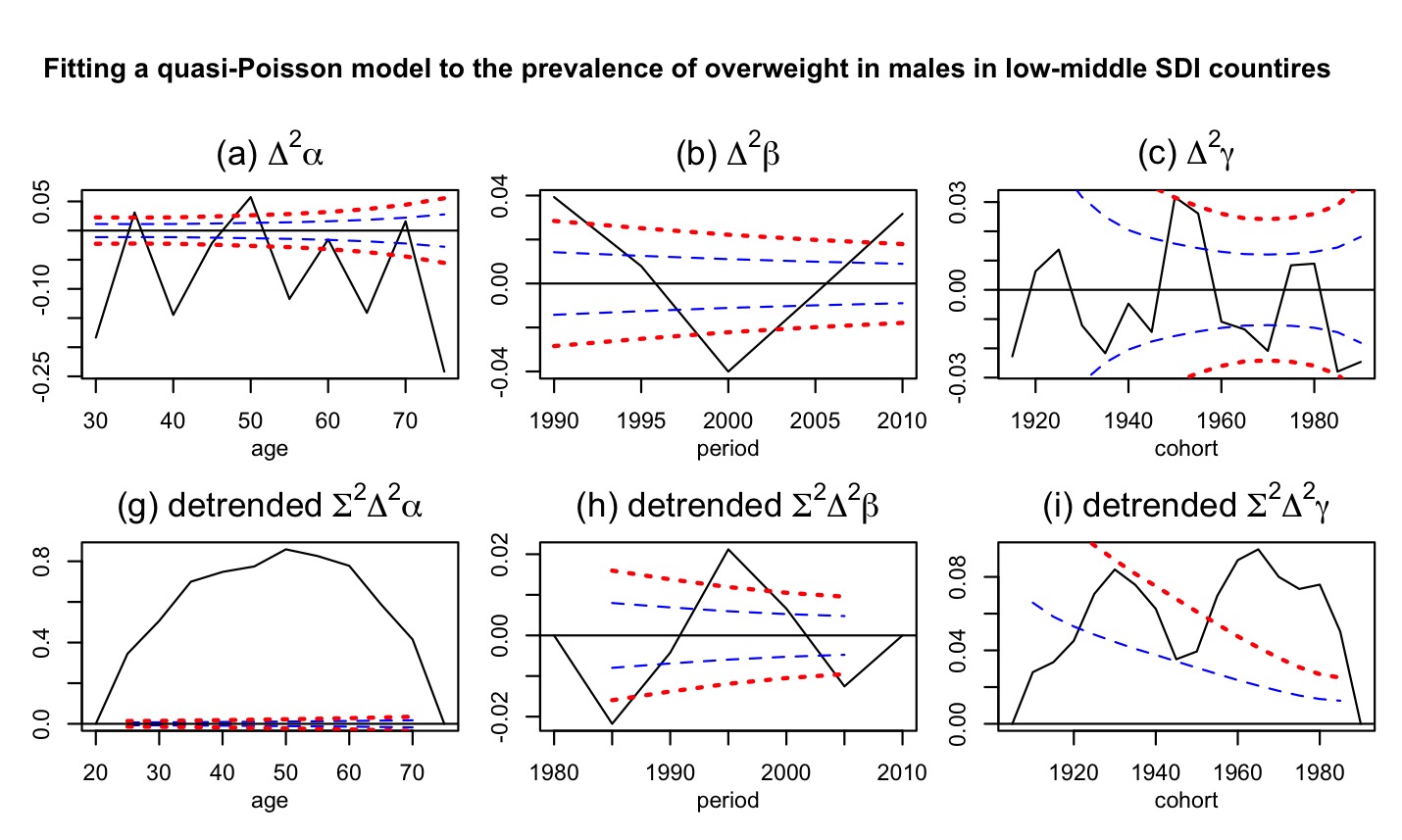


Supplementary Figure 65. Plots of the results of the APC model fit on overweight prevalence in males in the Low-Middle SDI countries.


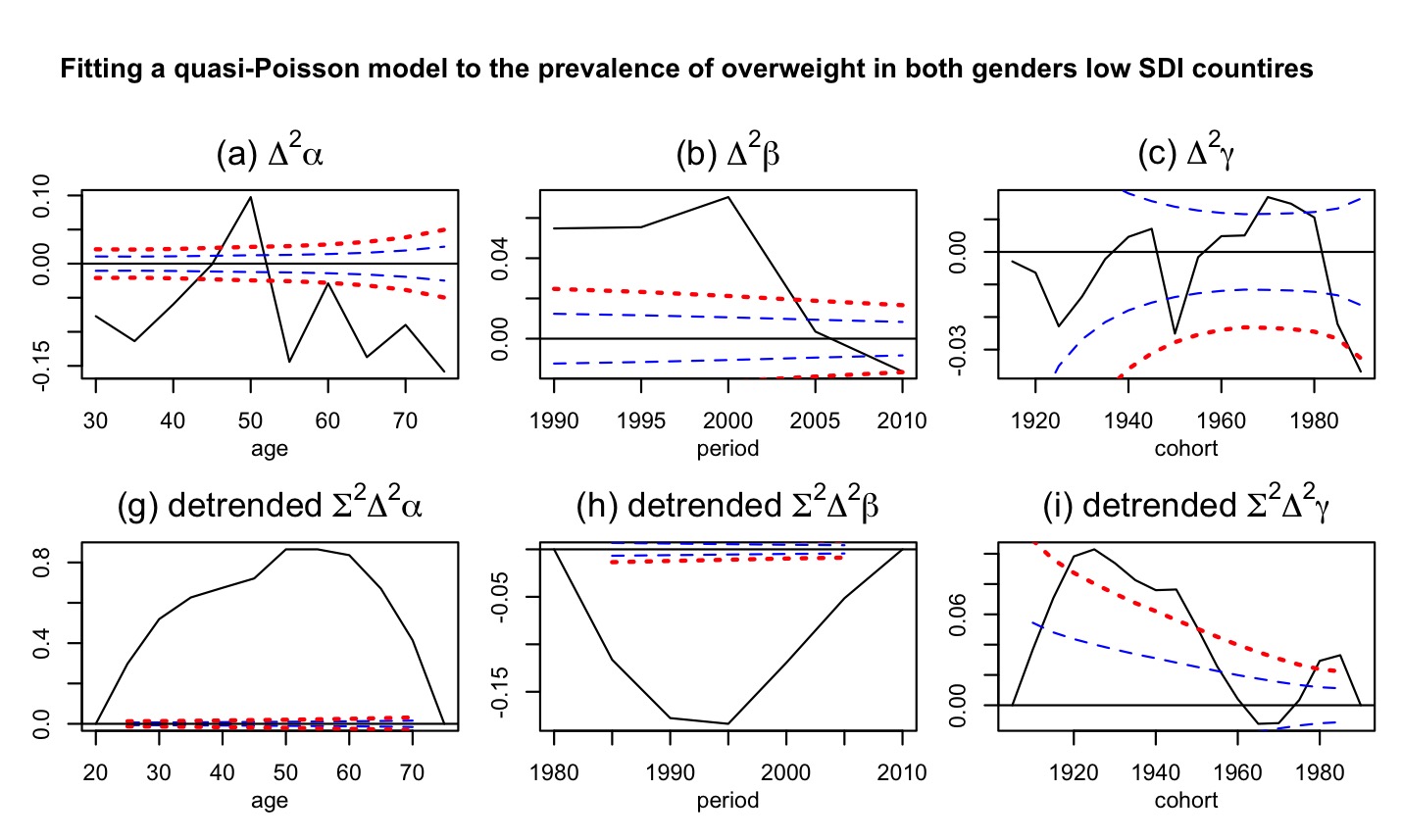


Supplementary Figure 66. Plots of the results of the APC model fit on overweight prevalence in all population in the Low SDI countries.


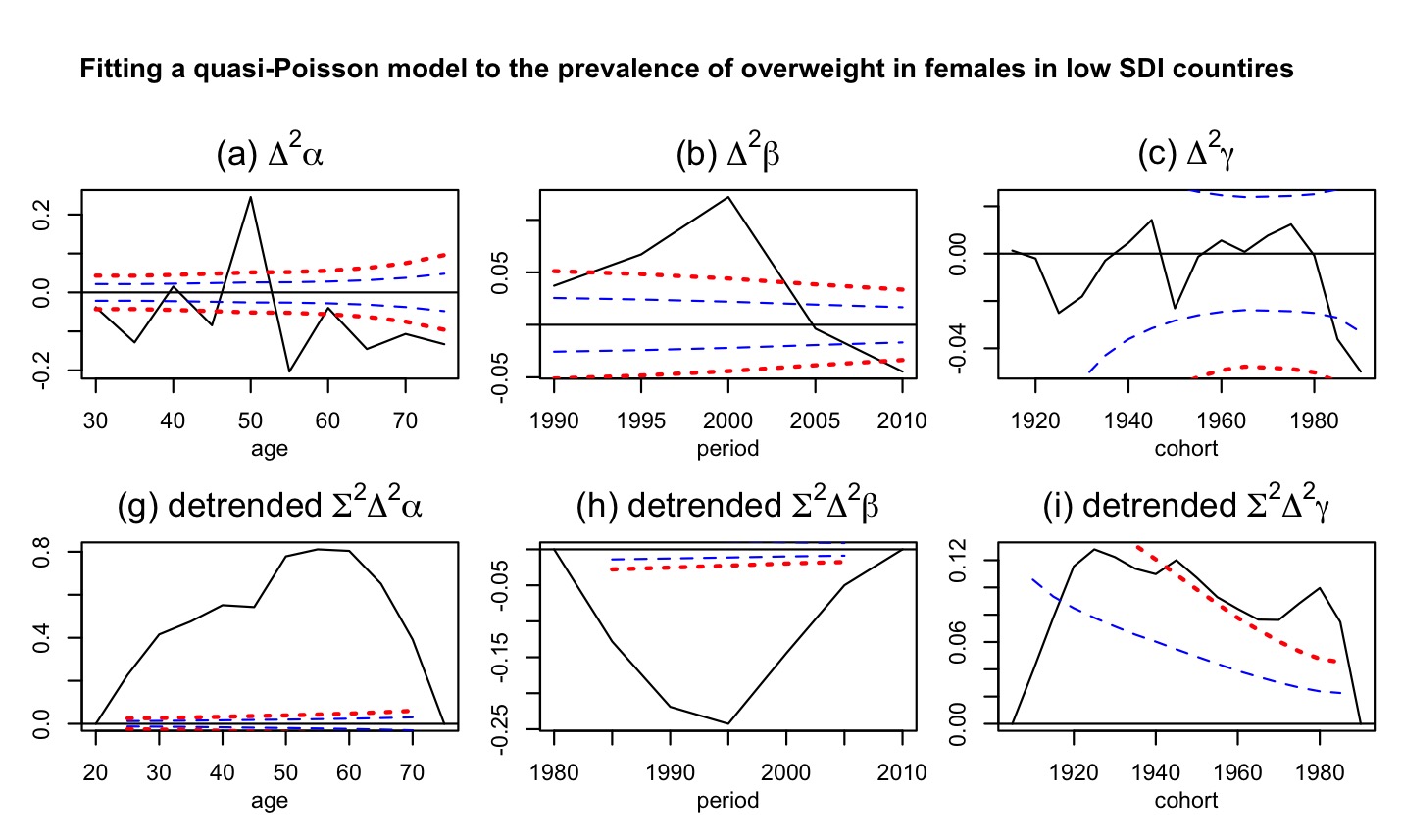


Supplementary Figure 67. Plots of the results of the APC model fit on overweight prevalence in females in the Low SDI countries.


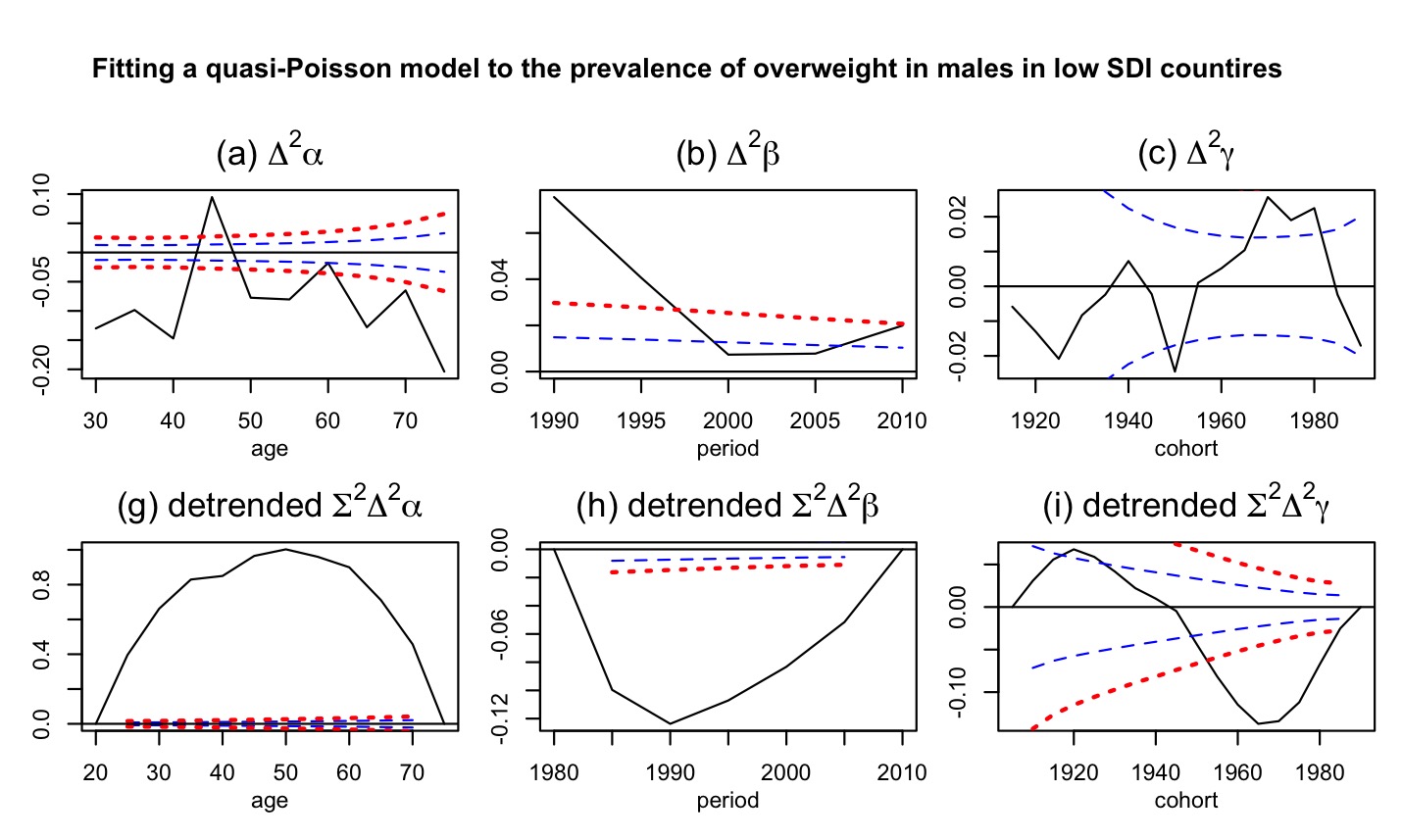


Supplementary Figure 68. Plots of the results of the APC model fit on overweight prevalence in males in the Low SDI countries

Supplementary Table 1. In this table, we showed the SDI of each country that their data was used based on the SDI subgroups.

| SDI categories | Countries |
| --- | --- |
| High SDI countries | Andorra, Antigua and Barbuda, Australia, Austria, Belarus, Belgium, Bermuda, Brunei, Canada, Cyprus, Czech Republic, Denmark, Estonia, Finland, France, Germany, Guam, Hungary, Iceland, Ireland, Israel, Italy, Japan, Kuwait, Latvia, Lithuania, Luxembourg, Netherlands, New Zealand, Northern Mariana Islands, Norway, Poland, Puerto Rico, Russia, Singapore, Slovakia, Slovenia, South Korea, Sweden, Switzerland, Taiwan, The Bahamas Trinidad and Tobago, United Arab Emirates, United Kingdom, United States, Virgin Islands, U.S. |
| High‐middle SDI countries | Albania, American Samoa, Argentina, Armenia Azerbaijan, Bahrain, Barbados, Bosnia and Herzegovina, Bulgaria, Chile, Colombia, Costa Rica, Croatia, Cuba, Dominica, Dominican Republic, Ecuador, Fiji, Georgia, Greece, Greenland, Grenada, Iran, Jamaica, Jordan, Kazakhstan, Lebanon, Macedonia, Malaysia, Malta, Mauritius, Moldova, Mongolia, Montenegro, Oman, Panama, Peru, Portugal, Qatar, Romania, Saint Lucia, Saint Vincent and the Grenadines, Saudi Arabia, Serbia, Seychelles, Spain, Sri Lanka, Suriname, Thailand, Turkey, Turkmenistan, Ukraine, Uruguay, Uzbekistan, Venezuela |
| Middle SDI countries | Algeria , Belize, Bolivia, Botswana, Brazil, China, Egypt, El Salvador, Equatorial Guinea, Federated States of Micronesia, Gabon, Guyana, Honduras, Indonesia, Iraq, Kyrgyzstan, Libya, Maldives, Marshall Islands, Mexico, Namibia, Nicaragua, North Korea, Palestine, Paraguay, Philippines, Samoa, South Africa, Swaziland, Syria, Tajikistan, Tonga, Tunisia, Vietnam |
| Low-middle SDI countries | Angola, Bangladesh, Bhutan, Cambodia, Cameroon, Cape Verde, Congo, Djibouti, Ghana, Guatemala, Haiti, India, Kiribati, Laos, Lesotho, Morocco, Myanmar, Nepal, Nigeria, Pakistan, Papua New Guinea, Sao Tome and Principe, Solomon Islands, Sudan, Tanzania, Timor‐Leste, Vanuatu, Yemen, Zambia, Zimbabwe |
| Low SDI countries | Afghanistan, Benin, Burkina Faso, Burundi, Central African Republic, Chad, Comoros, Cote d'Ivoire, Democratic Republic of the Congo, Eritrea, Ethiopia, Guinea, Guinea‐Bissau, Kenya, Liberia, Madagascar, Malawi, Mali, Mauritania, Mozambique, Niger, Rwanda, Senegal, Sierra Leone, Somalia, South Sudan, The Gambia, Togo, Uganda |
