# Supplementary material for: Prevalence patterns of overweight and obesity in the world: An age-period-cohort analysis
Source: PLoS One. 2025 Jun 18;20(6):e0324733. doi: 10.1371/journal.pone.0324733 (PMC12176128; doi:10.1371/journal.pone.0324733)
Supplement: Attachment — Submitted filename: PONE-D-24-42238_revised.pdf [file pone.0324733.s002.pdf]

PLOS ONE

Prevalence patterns of overweight and obesity in the world: An Age-Period-Cohort analysis

--Manuscript Draft--

**Manuscript Number:**	PONE-D-24-42238
**Article Type:**	Research Article
**Full Title:**	Prevalence patterns of overweight and obesity in the world: An Age-Period-Cohort analysis
**Short Title:**	Global prevalence of obesity and overweight: APC analysis
**Corresponding Author:**	Ali Kabir<br>Iran University of Medical Sciences<br>Tehran, IRAN, ISLAMIC REPUBLIC OF
**Keywords:**	Obesity; overweight; prevalence; Age-Period-Cohort analysis
**Abstract:**	Purpose: This study aimed to evaluate the trends of obesity and overweight among adolescents using the age-period-cohort (APC) analysis.<br>Methods: Data for this study was provided by Institute for Health Metrics and Evaluation. Our data represents the cumulative prevalence trend of obesity and overweight in 195 countries between 1990 and 2015 in 5-year intervals. The age intervals were also considered to be 5 years. To perform APC analysis, R program software was used.<br>Results: Our analysis, showed that the trend of obesity and overweight inflated during the study period; however, this inflation lost his pace in recent cohorts. After fitting models to data, we understood that APC model was the best fitted model for our data. The trend for both outcomes (obesity and overweight) were similar. We observed that of the all three parameters, age had the most effect on the trend and the highest canonical coefficients. Also, the parameters had almost similar effect on both gender subgroups.<br>Conclusion: Overall, this study shows that the trend of obesity and overweight is increasing; however, the rates started to decline in recent generations (cohort effect) indicating that the modern lifestyle started to change in favor of become healthier.
**Order of Authors:**	Ali Kabir
	Arman Karimi Behnagh
**Additional Information:**	
**Question**	**Response**
**Financial Disclosure**<br><br>Enter a financial disclosure statement that describes the sources of funding for the work included in this submission. Review the submission guidelines for detailed requirements. View published research articles from *PLOS ONE* for specific examples.<br><br>This statement is required for submission and **will appear in the published article** if the submission is accepted. Please make sure it is accurate.	Yes

Powered by Editorial Manager® and ProduXion Manager® from Aries Systems Corporation

**Funded studies**<br>Enter a statement with the following details:<br>• Initials of the authors who received each award<br>• Grant numbers awarded to each author<br>• The full name of each funder<br>• URL of each funder website<br>• Did the sponsors or funders play any role in the study design, data collection and analysis, decision to publish, or preparation of the manuscript?<br><br>Did you receive funding for this work?	
Please add funding details.<br>as follow-up to "**Financial Disclosure**<br><br>Enter a financial disclosure statement that describes the sources of funding for the work included in this submission. Review the submission guidelines for detailed requirements. View published research articles from *PLOS ONE* for specific examples.<br><br>This statement is required for submission and **will appear in the published article** if the submission is accepted. Please make sure it is accurate.<br><br>**Funded studies**<br>Enter a statement with the following details:<br>• Initials of the authors who received each award<br>• Grant numbers awarded to each author<br>• The full name of each funder<br>• URL of each funder website<br>• Did the sponsors or funders play any role in the study design, data collection and analysis, decision to publish, or preparation of the manuscript?<br><br>Did you receive funding for this work?"	This work was supported by National Institute for Medical Research Development (NIMAD) (Grant numbers [971515]). Dr. Ali Kabir has received this research grant.
Please select the country of your main research funder (please select carefully as in some cases this is used in fee calculation).	IRAN, ISLAMIC REPUBLIC OF - IR

Powered by Editorial Manager® and ProduXion Manager® from Aries Systems Corporation

as follow-up to "**Financial Disclosure**<br><br>Enter a financial disclosure statement that describes the sources of funding for the work included in this submission. Review the submission guidelines for detailed requirements. View published research articles from *PLOS ONE* for specific examples.<br><br>This statement is required for submission and **will appear in the published article** if the submission is accepted. Please make sure it is accurate.<br><br>**Funded studies**<br>Enter a statement with the following details:<br>• Initials of the authors who received each award<br>• Grant numbers awarded to each author<br>• The full name of each funder<br>• URL of each funder website<br>• Did the sponsors or funders play any role in the study design, data collection and analysis, decision to publish, or preparation of the manuscript?<br><br>Did you receive funding for this work?"	
**Competing Interests**<br><br>Use the instructions below to enter a competing interest statement for this submission. On behalf of all authors, disclose any competing interests that could be perceived to bias this work—acknowledging all financial support and any other relevant financial or non-financial competing interests.<br><br>This statement is required for submission and **will appear in the published article** if the submission is accepted. Please make sure it is accurate and that any funding sources listed in your Funding Information later in the submission form are also declared in your Financial Disclosure statement.	The authors have declared that no competing interests exist.

View published research articles from *PLOS ONE* for specific examples.<br><br>**NO authors have competing interests**<br><br>Enter: *The authors have declared that no competing interests exist.*<br><br>**Authors with competing interests**<br><br>Enter competing interest details beginning with this statement:<br><br>*I have read the journal's policy and the authors of this manuscript have the following competing interests: [insert competing interests here]*<br><br>* typeset	
**Ethics Statement**<br><br>Enter an ethics statement for this submission. This statement is required if the study involved:<br><br>• Human participants<br>• Human specimens or tissue<br>• Vertebrate animals or cephalopods<br>• Vertebrate embryos or tissues<br>• Field research<br><br>Write "N/A" if the submission does not require an ethics statement.<br><br>General guidance is provided below. Consult the submission guidelines for detailed instructions. **Make sure that all information entered here is included in the Methods section of the manuscript.**	"N/A"

**Format for specific study types**<br><br>**Human Subject Research (involving human participants and/or tissue)**<br>• Give the name of the institutional review board or ethics committee that approved the study<br>• Include the approval number and/or a statement indicating approval of this research<br>• Indicate the form of consent obtained (written/oral) or the reason that consent was not obtained (e.g. the data were analyzed anonymously)<br><br>**Animal Research (involving vertebrate animals, embryos or tissues)**<br>• Provide the name of the Institutional Animal Care and Use Committee (IACUC) or other relevant ethics board that reviewed the study protocol, and indicate whether they approved this research or granted a formal waiver of ethical approval<br>• Include an approval number if one was obtained<br>• If the study involved *non-human primates*, add *additional details* about animal welfare and steps taken to ameliorate suffering<br>• If anesthesia, euthanasia, or any kind of animal sacrifice is part of the study, include briefly which substances and/or methods were applied<br><br>**Field Research**<br><br>Include the following details if this study involves the collection of plant, animal, or other materials from a natural setting:<br>• Field permit number<br>• Name of the institution or relevant body that granted permission	
**Data Availability**<br><br>Authors are required to make all data underlying the findings described fully available, without restriction, and from the time of publication. PLOS allows rare exceptions to address legal and ethical concerns. See the PLOS Data Policy and FAQ for detailed information.	Yes - all data are fully available without restriction

A Data Availability Statement describing where the data can be found is required at submission. Your answers to this question constitute the Data Availability Statement and **will be published in the article**, if accepted.<br><br>**Important:** Stating 'data available on request from the author' is not sufficient. If your data are only available upon request, select 'No' for the first question and explain your exceptional situation in the text box.<br><br>Do the authors confirm that all data underlying the findings described in their manuscript are fully available without restriction?	
**Describe where the data may be found in full sentences. If you are copying our sample text, replace any instances of XXX with the appropriate details.**<br><br>• If the data are **held or will be held in a public repository**, include URLs, accession numbers or DOIs. If this information will only be available after acceptance, indicate this by ticking the box below. For example: *All XXX files are available from the XXX database (accession number(s) XXX, XXX.*).<br>• If the data are all contained **within the manuscript and/or Supporting Information files**, enter the following: *All relevant data are within the manuscript and its Supporting Information files.*<br>• If neither of these applies but you are able to provide **details of access elsewhere**, with or without limitations, please do so. For example:<br><br>*Data cannot be shared publicly because of [XXX]. Data are available from the XXX Institutional Data Access / Ethics Committee (contact via XXX) for researchers who meet the criteria for access to confidential data.*<br><br>*The data underlying the results presented in the study are available from (include the name of the third party*	The dataset used for this stud was formerly available in the https://vizhub.healthdata.org/gbd-results/<br>However, in the current version of the website, the dataset is no longer available, due to GBD database updates. But the dataset is free accessible from corresponding authors without any restriction.

Powered by Editorial Manager® and ProduXion Manager® from Aries Systems Corporation

and contact information or URL).<br>• This text is appropriate if the data are owned by a third party and authors do not have permission to share the data.<br><br>* typeset	
Additional data availability information:	

Powered by Editorial Manager® and ProduXion Manager® from Aries Systems Corporation

Manuscript

Click here to access/download;Manuscript;APC manuscript (2) (2) (AutoRecovered).docx

Prevalence patterns of overweight and obesity in the world: An Age-Period-Cohort analysis

Ali Kabir, MD, MPH, MSc, PhD[1*], Arman Karimi Behnagh, MD[1]

[1]: Minimally Invasive Surgery Research Center, Iran University of Medical Sciences, Tehran, Iran

***Corresponding author: Ali Kabir**

Associate Professor of Epidemiology, Minimally Invasive Surgery Research Center, Iran University of Medical Sciences, Tehran, Iran

Address: Minimally Invasive Surgery Research Center, Mansouri Street, Niayesh Street, Sattarkhan Avenue, Tehran, Tehran, Iran

Mobile: +98912-388-5570

Tel: +9821-8670-2227

Fax: +9821-8670-2227

PO: 1449614535

E-mail: kabir.a@iums.ac.ir; aikabir@yahoo.com

Abstract

Purpose: This study aimed to evaluate the trends of obesity and overweight among adolescents using the age-period-cohort (APC) analysis.

Methods: Data for this study was provided by Institute for Health Metrics and Evaluation. Our data represents the cumulative prevalence trend of obesity and overweight in 195 countries between 1990 and 2015 in 5-year intervals. The age intervals were also considered to be 5 years. To perform APC analysis, R program software was used.

Results: Our analysis, showed that the trend of obesity and overweight inflated during the study period; however, this inflation lost his pace in recent cohorts. After fitting models to data, we understood that APC model was the best fitted model for our data. The trend for both outcomes (obesity and overweight) were similar. We observed that of the all three parameters, age had the most effect on the trend and the highest canonical coefficients. Also, the parameters had almost similar effect on both gender subgroups.

Conclusion: Overall, this study shows that the trend of obesity and overweight is increasing; however, the rates started to decline in recent generations (cohort effect) indicating that the modern lifestyle started to change in favor of become healthier.

Keywords: Obesity, Overweight, Prevalence, Age-Period-Cohort analysis

Introduction

In recent decades, the epidemic of obesity and overweight has become a global point of concern for health care systems, especially due to its influence on chronic conditions and overall life expectancy (1, 2). Owing to a combination of environmental factors, such as economic growth, sedentary lifestyle and increased intake of processed and ultra-processed food, the prevalence of obesity has been climbing steeply (1, 2). According to the latest World Health organization (WHO) report on obesity and overweight, the population of obese and overweight increased more than double since 1990 and currently almost 2.5 billion adult people live with obesity or overweight (3).

Comparing to normal population, obese population are at higher risk of poor health outcome during their life course(4, 5). Excess body weight is associated with adverse health outcomes on longevity, disability-free life years, quality of life, and productivity (4, 5). Moreover, a growing prevalence of childhood obesity, in particular, predicts an overwhelming burden of disease in the decades to come(6). However, such patterns can be predicted and also the high-risk groups can be determined using particular statistical methods. Very recently, there have been reports on the trend of global obesity globally. The results of these studies further support the finding of WHO report (5, 7).

Age period cohort (APC) analysis is a statistical approach by which time-varying variables may be better understood. In the field of epidemiology, these analyses are employed to distinguish the effects of three types of variables: age, period, and cohort(8). Age effects are the result of the intertwined social and biological factors which are often specific to each individual population. It must be noted that age effects arise from physiological alterations and social experiences only related to ageing. These effects are usually characterized by the different disease profiles observed in each age group (9). Extrinsic elements affecting all age groups in a similar manner, such as environmental, economic and geopolitical factors, exert their influence in the form of period effects.

It is important to know that changes to any part of the study methodology, such as definitions, data gathering or taxonomy is observed in the form of period effects(10). The final parameter of interest in APCs is the cohort effect, which are caused by the unique event exposures of population subgroups throughout time. Birth cohort is the most common among the cohort effects and is defined as the differences in outcome risks based on the year of birth (11). Cohort effects reflect the differences in distribution between age groups when faced with the same exposure. To simplify, cohort effects are experienced differently by each age group due to the result of age-specific features or

susceptibility to an exposure or event (11). In this study, we aimed to address the effect of each parameter in trend of overweight and obesity worldwide.

Method and Materials

Data source and description

Data was provided by the IHME, based on a study of GBD series(12). Despite improvements in quality of the data modelled and produced by IHME, there are several reasons that made us use an older version of GBD studies' datasets and that is the fact that in the recent databases, the obesity and overweight data were provided in a combined manner and under title of "high BMI". Thus, due to clear purpose of the current study, it was decided to use older datasets, in which the data of obesity and overweight prevalence reported separately.

According to the reference study (12), after data gathering, a mixed effects model was used to approximate and correct the data of each GBD region and age group due to particular biases like the self-report. Moreover, spatiotemporal Gaussian process regression was used to obtain the mean prevalences of overweight and obesity. In case of any missing data existence, the data was calculated from parameters and covariates of neighboring countries data using a linear model.

According to above mentioned explanations, we used the data of 195 countries to estimate the global prevalence of obesity and overweight. The mean number and rate of obese and overweight population based on different age and sex groups was used for our analysis. As pre-requisite of APC analysis, the age and period intervals were set to be 5-year. The data on world population was obtained from same source (https://ghdx.healthdata.org/record/ihme-data/gbd-2015-obesity-and-overweight-prevalence-1980-2015) in 18th of June 2018. Due to the structure of our dataset for obesity and overweight, we restructured data of world population in a 5 years interval for periods and age groups. A similar approach was employed for gender subgroups.

The challenges of APC analysis

Our data on obesity and overweight prevalence is comprised of data on prevalence rate in a consecutive number of years. To run an APC analysis, the model below is employed as a standard to discriminate the effects of age, period, and cohort from each other:

$$(1) \qquad \mu = \alpha_{age} + \beta_{period} + \gamma_{cohort} + \delta$$

Since each parameter of in this equation can be determined if the two other parameters are available. This rises a considerable collinearity between the parameters, which make it hard to the specify the exclusive effect of each parameter(13). This problem is called as identification problem in APC analysis, and so far, many solutions have been proposed to solve this problem(14). As such solutions, constrained coefficients GLIM estimator, proxy variables approach, nonlinear parametric transformation approach, log-linear models, and intrinsic estimator method can be mentioned(8, 11, 14-16).

As a result of linear dependency between the three components provided in equation (1), the model lacks ability to of identify the effect of each parameter in its current form. By knowing two out of three components, the other component of the equation can also be determined. As an instance, if one knows the value of age and period the third parameter, cohort, can subtracting the value of age from the value of period. Thus, this allows for an infinite solution pool, it becomes impossible to produce a correct estimate for the individual effect of each of the parameters. As a result, the unique assignment of the observed trends to each component becomes impossible.

Such an approach is faced with two inherent complications. First, strong prior information is crucial to the assumptions underlying the imposed constraints and empirical validation of these assumptions is not possible. Second, the results of the analyses are heavily influenced by these constraints, to a degree that different age, period, and cohort estimates may be estimated based on the choice of identified constraints (17). To date, numerous approaches have tried to address the identification conundrum in APC analysis. The estimable function, hierarchical-model approach and constrained generalized linear model are examples of such endeavors, however, they still rely heavily on the imposed constraints and assumptions(17).

In this study, we have employed the method introduced by Kuang et al.(16) and Nielsen et al.(18). This model is parametrized in terms of a freely varying vector provided by the following: three initial points, functions of the unidentified first differences, and the complete set of second differences of the time effects. Kuang et al. considered a parameter (i.e. prevalence rate) shaped by the second differences and the three predictor entries:

$$(2)\quad \xi = (\mu_{i1j1}, \mu_{i2j2}, \mu_{i3j3}, \Delta^2 \alpha_3 \ldots, \Delta^2 \alpha_I, \Delta^2 \beta_3 \ldots, \Delta^2 \beta_J, \Delta^2 \gamma_{h1+3} \ldots, \Delta^2 \gamma_{K-h2})$$

In this equation (2), I, J and K represent indices of age, period and cohort, and L + 1 is the lowest index of period. As a result, the contribution of age, cohort and period is modeled with regards to the cumulative sums of the double differences with respect to age, period and cohort, which are identifiable.

Statistical analysis

We estimated obesity and overweight prevalence rate for each five-year period from 1990 to 2015. To construct the rate of obesity and overweight prevalence, we used the world population data in a similar structure as the obesity and overweight prevalence. To perform APC analysis, we used the prevalence of obese and overweight individuals which plotted separately by age at report time, year of report, and birth cohort. Akaike Information Criterion (AIC) was used to assess the model fitness and smaller values indicate better fit. Deviance was calculated using two parameters, degree of freedom and log-likelihood. The following formula was considered: Deviance = − 2 × the difference in log-likelihood values for the nested models. In order to evaluate the impact of different time periods and cohorts, we compared the performance of models that did not include these specific terms (such as models with only one time parameter, PC, AC, and AP) with the performance of the model that included all three terms, APC. For the purpose of this study, we calculate the prevalence rate of obesity and overweight in each period, by dividing the number of people of with BMI>30 (for obesity) and 25>BMI>30 (for overweight) to number of people in the specific period. The R package “apc” was used to performed all the statistical analysis. All the analysis was performed in R (version 4.2.1, R core team, 2022).

Results

The global prevalence of obesity is growing. Prevalence increased with age and then declined however there is no consistent pattern on the peak respecting to the age groups. Recent periods had higher number of obese populations, however, the age distribution of obesity over different periods had slight changes (Fig 1 A). According to Fig 1&2, it can be seen that the rate of obesity is the highest in age of 50s in almost all periods. Also, in close inspection of the Fig 1, the rate decreases until age of 80, and after that the rate of obesity increased. It is of vital importance to mentioned that there is a decreasing trend in number of both total population and number obese people in older ages, however the rate of obesity prevalence increased after age of 80.

Moreover, the prevalence of obesity was rising in the same age groups of different periods and the earlier periods had lower rate of obesity in comparison to recent periods in the same age ranges (Fig. 2 A). Interestingly, the pattern was not completely parallel for all cohorts. In different periods the lowest rate of obesity belongs to younger age group such as 20s and 30s. Our subgroup analysis based on sex, revealed similar patterns. However, according to Fig. 1B the rate of obesity in females fluctuated between 0.01 to 0.2. This fluctuation for male population was ranged between 0.02 to 0.14 (Fig. 1C). Besides, the trends were very similar. In other words, the only difference between two sex subgroups was the value of rates however, the trend is almost the same.

Apart from obesity, the prevalence of overweight has also increased. This rising in the prevalence of overweight has been associated with age. Similar to obesity, in the recent periods the rate of overweight was higher and the age distribution of overweight has changed slightly in different periods. The lowest rate of overweight in each period belonged to younger age groups (Fig. 3). Furthermore, the lowest rate of obesity belongs to very young age groups including the population of 20s (Fig. 3). According to the Fig. 4, it can be seen that, the prevalence of overweight increased dramatically in each age group in recent periods.

After describing different trends of obesity and overweight based on the three-time varying parameters, we compared the different possible model estimations based on these three elements. Table 1 shows the deviance table for the global prevalence of obesity. Based on AIC value (-795.657) the APC model was the preferred model to be selected. Although, the age-cohort model showed better value for AIC, however, there was not significant difference between the two model (APC vs AC); Hence, we decided to continue our analysis using the APC model.

Table 2 presents the deviance table for the global overweight prevalence. Unlike the obesity, AIC value for the APC model was the lowest possible value (-868.014) and none of the other sub-models did better than APC. Therefore, the APC model was used to calculate the effect of three time-related parameter on prevalence of overweight.

There are different tools provided by the “apc” package of R to check the goodness of fit of the selected model (here APC model). Nonetheless, for the purpose of this study, we only include the probability transform plot. This graph shows for a specific age and period, the likelihood of retrieving the observed age-specific rates. Here, we checked the probability transform plot for the two different link functions to measure each parameter.

Usually, in majority of the available tools for APC analysis, the logarithmic link function (a Poisson model) used to calculate the coefficients of the each of the three parameters; however, according to the Figs. 5&6, using this link function usually provided observed values falling in the 1% tail of the fitted distribution (red triangles). Therefore, we select a gaussian model for the APC analysis. As can be seen in Figs. 5&6, implementing such a strategy provided a model with better goodness of fit.

The estimated reparametrized time effects were depicted in supplementary Fig. 7 and 8 for trend of obesity and overweight, respectively. In the first row of Fig. 7 and Fig. 8 the series of estimated double differences for each of age, period, and cohort can be observable. The cumulative effect of double differences at each age, period, and cohort is shown in the second row of plots. According to the second row in Figs. 7 and 8, age has accelerating effect to age 50, for the period the same effect can be seen at period of 2000 however in cohort similar effect is seen after the for the 1940’s cohorts and after. Based on supplementary Fig. 2A similar effect can be seen, however there are small shifts on time points in each time parameters. The model selection for each sex subgroup was demonstrated in supplementary tables 1-4. Based on the parameters represented in these two tables, again APC was the best model that fit the data. The results on subgroup analysis regarding each parameters effect is provided on supplementary Figs. 3-6.

Discussion

This study showed increasing trend in obesity and overweight in the worldwide. However, unlike other similar studies, in this report an ACP model was implemented to understand changes in the prevalence of obesity in a global scale.

Previously, the impact of period and cohort effects have been evaluated on obesity prevalence in a national scale (19-21). Although the APC analysis has been used on trend of different epidemiological and social outcomes, this study was the first study using this method on the prevalence of overweight and obesity in aggregated data of 195 countries.

Our study shows that almost in all periods, the people in the age of 50 had the highest rate of obese and overweight comparing to other age groups. After this group, age of 40 and 60 had the highest rate of obese people, respectively. This indicates that the trend of obesity is increasing by the age of 50 however after this age the number of this population started to decrease.

The increasing prevalence of obesity in recent decades is known as a global epidemic(22). Finkelstein et al. suggested that the prevalence of obesity will continue to increase rapidly, with a 30% rise in prevalence by 2030(23). Obesity is a complex interaction between genetic susceptibility and environmental factors(24). Recent decades have been marked by changes in the food production, storage and processing and consumption of processed foods and ready meals with high calorie and low cost that are more available, made person obese(25).

Based on our findings, it was observed that the effect of period as a time varying parameters, changed during our study period and it had positive impact on the trend in earlier periods, however, in the later periods it became a complete negative estimator of the trend. Since this study performed in global scale, major issues that impact whole nations (in varying scale) should be considered for explaining the transitions in effect of period. One key factor in this regard can be the development of new venues for behavioral changes, such as social medias. Earlier researches regarding the possible role of social media on development of obesity epidemy majorly emphasize on the positive effect of these new platforms on increasing the prevalence of obesity and overweight (26). However, later studies showed that physical, behavioral, and dietic interventions which provided through social media can pose great influence on reduction of obesity and overweight in general population(27).

Reducing barriers to trade and global commodities flow expands the Western diet, associated with increased consumption of sugar and fat and reduced consumption of legumes and vegetables, which is a factor in the rising prevalence of obesity in developed and developing countries(28). Low physical activity and sedentary lifestyles are risk factors of obesity and overweight(29). Growth of population size, population aging and urbanization especially in developing countries are the factors contributing epidemic of obesity(30). Changes in people's behavior and the increased number and marketing of snacks have considerable impact on weight gain and incident of obesity(31).

Obesity is a known predisposing factor for many chronic diseases, such as hypertension, different cancers, type 2 diabetes mellitus, and even psychological disorders(32). Our study shows the prevalence of younger adult obesity increased over the time. Younger adult obesity is linked with a increased risk of complications and one of the major concerns is that this population become obese adult with all the risks and complications(33). Severe obesity in younger age predicts development of obesity in adulthood and associated with more cardiovascular morbidity and higher risk of all-cause mortality (34).

In recent decades, improvements in public health and education, reducing mortality of infectious disease, advances in medical cares and management of risk factors reduced obese patient's mortality, making obese patients stay healthier and live longer. Our analyses show the prevalence of obesity in the age groups of 20 to 50 is rising, which may be due to obese children become obese adult, industrial life style and also interventions have not been effective in reducing obesity.

Similar to the rising rates of obesity, the occurrence of being overweight has also seen a growth with advancing age over the past few decades. This trend has led to a higher number of individuals falling into the category of being obese. We found that the rates of being overweight showed greater inflation in the younger cohorts than the older ones. In general, our study shows increases in percentage of overweight and obese people with age and period.

From our study it is apparent that peoples with overweight increased over the generations. Toxic environment reported in 1970 have contribution to increases in the population BMI and prevalence of overweight and in last decades it continued to become more toxic and obesogenic (35). Spending greater periods of life in obesogenic environment is related to greater risk of overweight of some generations. Later born cohorts are more susceptible to becoming overweight more rapidly meaning that prevention strategies can be effective in children and young adults and can prevent adult obesity.

Several limitations of our study should be noted. Our data is modeled and for some age groups, especially for older ages our data failed to achieve precise estimation of parameters. One major limitation of the current study, is the time span of our data. The data used in this study gives insight for trends of obesity and overweight for almost 10 years ago, however, it should be noted that there have been limited resources that provide very detailed data on prevalence of obesity and overweight in different age groups and periods. Even, in the current version of GBD studies, there are not subtypes for high BMI, such as obesity and overweight. This issue impede us from updating our time span and it

was somehow compulsory to work with this dataset. Also, since the duration of study period was limited, our predicted model unsuccessful to show considerable robustness, therefore we omitted the prediction model of APC analysis and only the analysis of determining effect of each parameter were provided in this study. However, APC analysis help to understand the prevalence of obesity in the worldwide, but the current study does not identify the causes of obesity and only examines the prevalence of obesity. Ecological fallacy is possible to occur. We were unable to examine countries in overweight, obesity and severe obesity separately. Due to lack of information, we could not study the prevalence of obesity in subgroups by race / ethnicity.

Conclusion

The analysis obtained from our study provides a better understanding of the effect of age, period, and cohort on the prevalence of overweight and obesity. Changes in social, cultural, and economic landscapes have the potential to influence individuals' lives on a global scale, leading to widespread impacts such as the increasing prevalence of obesity.

Declarations

Ethics approval and consent

The present study is in agreement with the Declaration of Helsinki. The Research Ethics Committees of National Institute for Medical Research Development (NIMAD) approved the study protocol with ethics code: IR.NIMAD.REC.1397.015 at 1 July 2018.

Consent for publication

There is an ethics approval from NIMAD. Moreover, data from all countries have been provided from Institute for Health Metrics and Evaluation (IHME). No consent for publication was applicable in this study, since this study does not include any experiment or any clinical intervention. It should be noted that the data used in this study was provided by IHME via https://vizhub.healthdata.org/gbd-results/ .

Availability of data and materials

Source: Institute for Health Metrics and Evaluation. Used with permission. All rights reserved. All the data used for this study can be obtained from https://ghdx.healthdata.org/record/ihme-data/gbd-2015-obesity-and-overweight-prevalence-1980-2015.

Competing interests

The authors declare no competing interests.

Funding

This work was supported by National Institute for Medical Research Development (NIMAD) (Grant numbers [971515]). Dr. Ali Kabir has received this research grant.

Authors' contributions

AKB: Data curation, formal analysis, writing – original draft, visualization, final approval, accountable to the whole of the study. AK: Conceptualization, project administration, funding acquisition, having accessed and verified data, validation, writing– review and editing, supervision, final approval, accountable to the whole of the study.

Acknowledgements

Research reported in this publication was supported by Elite Researcher Grant Committee under award number (971515) from the National Institute for Medical Research Development (NIMAD), Tehran, Iran. Also, we wish to warmly acknowledge the support of Institute For Health Metrics and Evaluation (IHME), University of Washington, USA for the data preparation of this project.

References

1. Health Effects of Overweight and Obesity in 195 Countries over 25 Years. New England Journal of Medicine. 2017;377(1):13-27.
2. Malik VS, Willett WC, Hu FB. Global obesity: trends, risk factors and policy implications. Nat Rev Endocrinol. 2013;9(1):13-27.
3. WHO. Obesity and overweight 2024 [Available from: https://www.who.int/news-room/fact-sheets/detail/obesity-and-overweight.
4. Abdelaal M, le Roux CW, Docherty NG. Morbidity and mortality associated with obesity. Ann Transl Med. 2017;5(7):161.

5. Chen Y, Ma L, Han Z, Xiong P. The global burden of disease attributable to high body mass index in 204 countries and territories: Findings from 1990 to 2019 and predictions to 2035. Diabetes Obes Metab. 2024.
6. Gonzalez-Alvarez MA, Lazaro-Alquezar A, Simon-Fernandez MB. Global Trends in Child Obesity: Are Figures Converging? Int J Environ Res Public Health. 2020;17(24).
7. Worldwide trends in underweight and obesity from 1990 to 2022: a pooled analysis of 3663 population-representative studies with 222 million children, adolescents, and adults. Lancet. 2024;403(10431):1027-50.
8. Yang Y, and Kenneth C. Land. Age-period-cohort analysis: new models, methods, and empirical applications. CRC Press, 2013.
9. Reither EN HR, Yang Y. Do birth cohorts matter? Age-period-cohort analyses of the obesity epidemic in the United States. Social science & medicine 2009;69(10):1439-48.
10. Keyes KM, Li G. Age–period–cohort modeling. Injury research: Theories, methods, and approaches: Springer; 2011. p. 409-26.
11. Keyes KM UR, Robinson W, Li G. What is a cohort effect? Comparison of three statistical methods for modeling cohort effects in obesity prevalence in the United States, 1971-2006. Soc Sci Med 2010;70(7):1100-8.
12. Collaborators GBDO, Afshin A, Forouzanfar MH, Reitsma MB, Sur P, Estep K, et al. Health Effects of Overweight and Obesity in 195 Countries over 25 Years. N Engl J Med. 2017;377(1):13-27.
13. O'Brien RM. Estimable functions in age-period-cohort models: a unified approach. Quality & Quantity. 2014;48(1):457-74.
14. Bell A. Age period cohort analysis: a review of what we should and shouldn't do. Ann Hum Biol. 2020;47(2):208-17.
15. Mdzinarishvili T, Sherman S. A heuristic solution of the identifiability problem of the age-period-cohort analysis of cancer occurrence: lung cancer example. PLoS One. 2012;7(4):e34362.
16. Kuang D, Nielsen B, Nielsen JP. Identification of the age-period-cohort model and the extended chain-ladder model. Biometrika. 2008;95(4):979-86.
17. Smith TR, Wakefield J. A Review and Comparison of Age–Period–Cohort Models for Cancer Incidence. Statistical Science. 2016;31(4):591-610, 20.
18. Nielsen B, Nielsen JP. Identification and forecasting in mortality models. ScientificWorldJournal. 2014;2014:347043.
19. Jacobsen BK, Njølstad I, Thune I, Wilsgaard T, Løchen ML, Schirmer H. Increase in weight in all birth cohorts in a general population: The Tromsø Study, 1974-1994. Archives of internal medicine. 2001;161(3):466-72.
20. Allman-Farinelli MA, Chey T, Bauman AE, Gill T, James WP. Age, period and birth cohort effects on prevalence of overweight and obesity in Australian adults from 1990 to 2000. European journal of clinical nutrition. 2008;62(7):898-907.
21. Kwon JW, Song YM, Park H, Sung J, Kim H, Cho SI. Effects of age, time period, and birth cohort on the prevalence of diabetes and obesity in Korean men. Diabetes care. 2008;31(2):255-60.
22. Obesity: preventing and managing the global epidemic. Report of a WHO consultation. World Health Organization technical report series. 2000;894:i-xii, 1-253.
23. Finkelstein EA, Khavjou OA, Thompson H, Trogdon JG, Pan L, Sherry B, et al. Obesity and severe obesity forecasts through 2030. American journal of preventive medicine. 2012;42(6):563-70.
24. Hruby A, Hu FB. The Epidemiology of Obesity: A Big Picture. PharmacoEconomics. 2015;33(7):673-89.
25. Alkerwi A, Crichton GE, Hébert JR. Consumption of ready-made meals and increased risk of obesity: findings from the Observation of Cardiovascular Risk Factors in Luxembourg (ORISCAV-LUX) study. The British journal of nutrition. 2015;113(2):270-7.
26. Christakis NA, Fowler JH. The spread of obesity in a large social network over 32 years. N Engl J Med. 2007;357(4):370-9.

27. Goodyear VA, Wood G, Skinner B, Thompson JL. The effect of social media interventions on physical activity and dietary behaviours in young people and adults: a systematic review. Int J Behav Nutr Phys Act. 2021;18(1):72.
28. Popkin BM, Adair LS, Ng SW. Global nutrition transition and the pandemic of obesity in developing countries. Nutrition reviews. 2012;70(1):3-21.
29. Jebb SA, Moore MS. Contribution of a sedentary lifestyle and inactivity to the etiology of overweight and obesity: current evidence and research issues. Medicine and science in sports and exercise. 1999;31(11 Suppl):S534-41.
30. Kelly T, Yang W, Chen CS, Reynolds K, He J. Global burden of obesity in 2005 and projections to 2030. International journal of obesity (2005). 2008;32(9):1431-7.
31. Caprio S, Santoro N, Weiss R. Childhood obesity and the associated rise in cardiometabolic complications. Nature Metabolism. 2020;2(3):223-32.
32. Daniels SR, Arnett DK, Eckel RH, Gidding SS, Hayman LL, Kumanyika S, et al. Overweight in children and adolescents: pathophysiology, consequences, prevention, and treatment. Circulation. 2005;111(15):1999-2012.
33. Poobalan A, Aucott L. Obesity Among Young Adults in Developing Countries: A Systematic Overview. Current obesity reports. 2016;5(1):2-13.
34. Bendor CD, Bardugo A, Pinhas-Hamiel O, Afek A, Twig G. Cardiovascular morbidity, diabetes and cancer risk among children and adolescents with severe obesity. Cardiovascular diabetology. 2020;19(1):79.
35. Cohen DA. Obesity and the built environment: changes in environmental cues cause energy imbalances. International journal of obesity (2005). 2008;32 Suppl 7(0 7):S137-42.

Table 1. Comparison of different models to Age-Period-Cohort model as reference for obesity prevalence

Model	-2logL	df.residual	LR vs.APC	df vs.APC	prob(>chi_sq)	AIC
**APC**	-877.657	56	NA	NA	NA	-795.657
**AP**	-750.394	75	127.264	19	0	-706.394
**AC**	-874.543	60	3.114	4	0.539	-800.543

**PC**	-533.705	70	343.952	14	0	-479.705
**A**	-471.869	80	405.789	24	0	-437.869
**P**	-433.432	90	444.225	34	0	-419.432
**C**	-411.477	75	466.18	19	0	-367.477

AIC: Akaike Information Criterion, LR statistics: likelihood ratio statistics, DF; degree of freedom

Table 2. Comparison of different models to Age-Period-Cohort model as reference for overweight prevalence

**Model**	**-2logL**	**df.residual**	**LR vs.APC**	**df vs.APC**	**prob(>chi_sq)**	**AIC**
**APC**	-950.014	56	NA	NA	NA	-868.014
**AP**	-706.338	75	243.676	19	0	-662.338
**AC**	-815.81	60	134.204	4	0	-741.81
**PC**	-435.474	70	514.54	14	0	-381.474
**A**	-560.298	80	389.716	24	0	-526.298
**P**	-342.951	90	607.063	34	0	-328.951
**C**	-392.863	75	557.151	19	0	-348.863

AIC: Akaike Information Criterion, LR statistics: likelihood ratio statistics, DF; degree of freedom

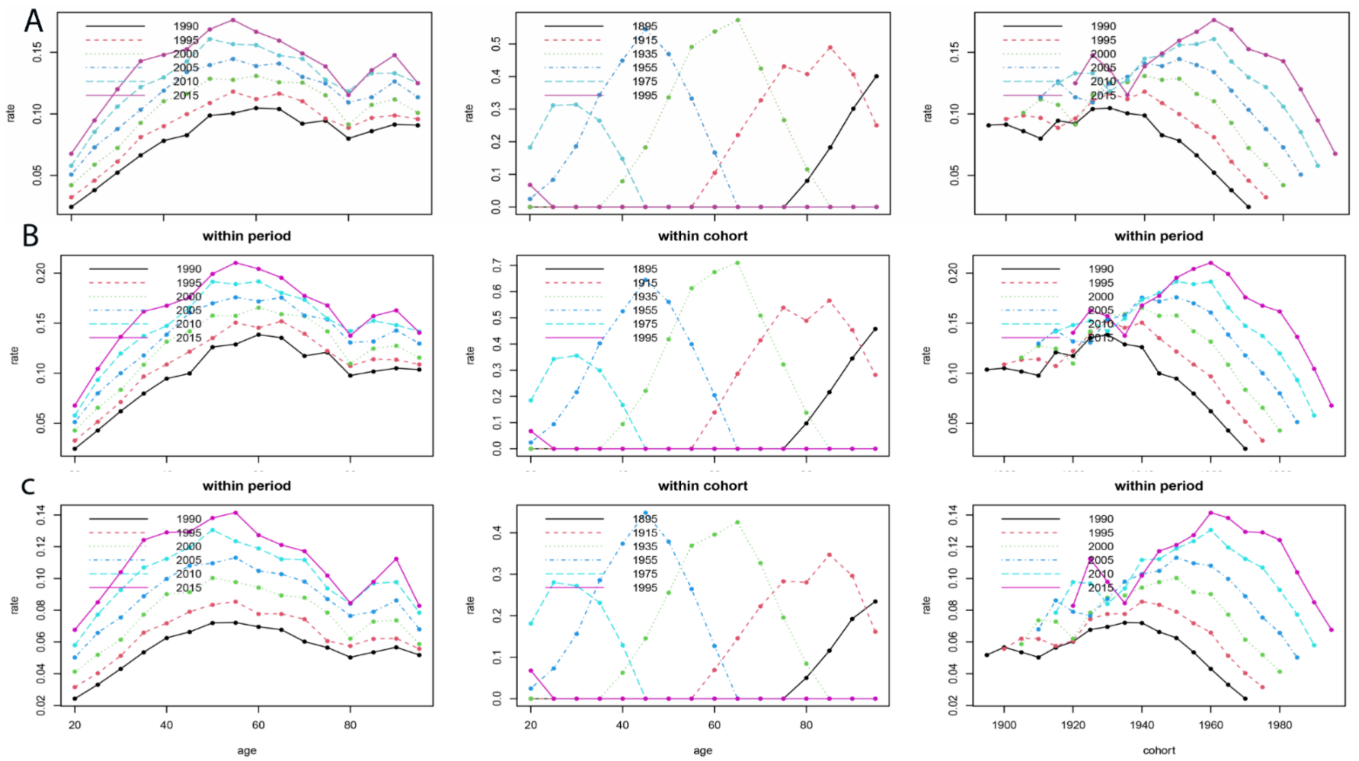

Figure 1. The trend of obesity prevalence rate within period-age, cohort-age, and period-cohort for both sexes(A), female(B), male(C)

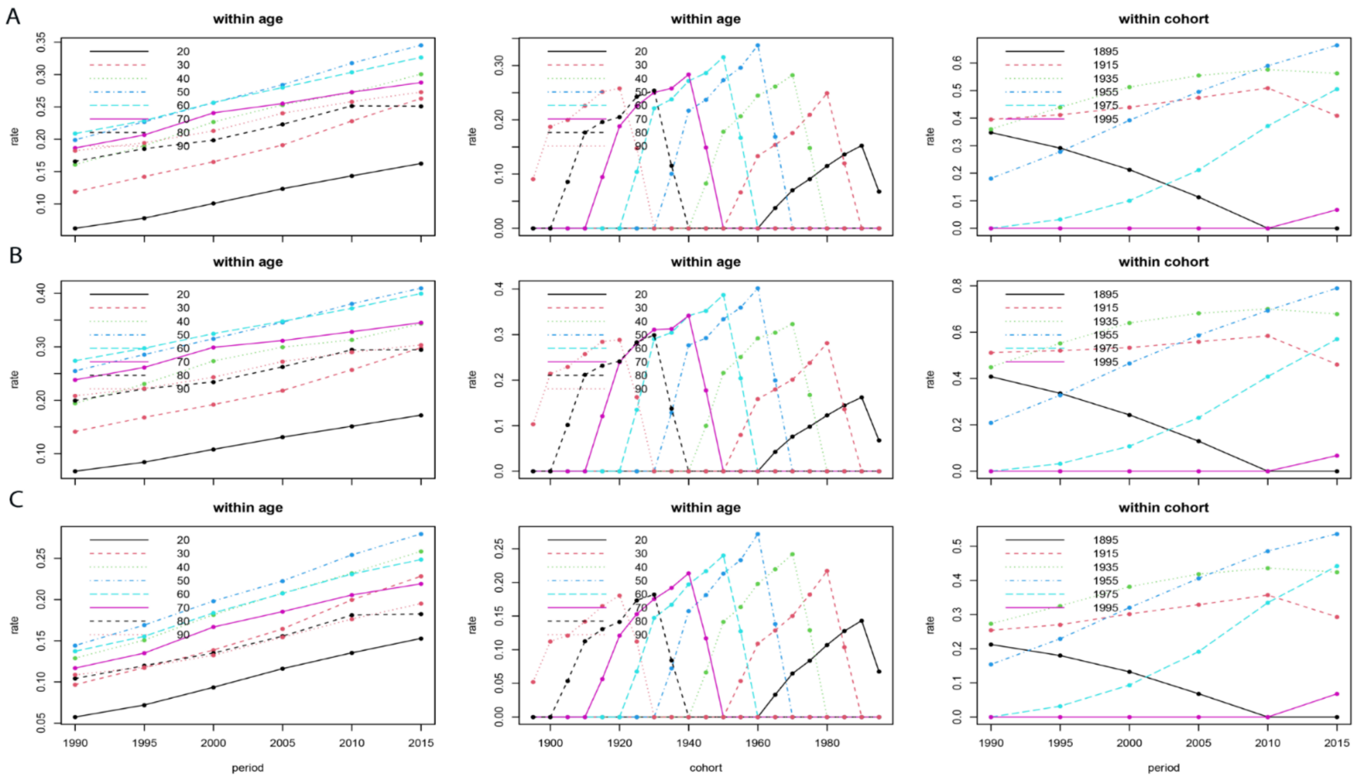

Figure 2. The trend of obesity prevalence rate within age-period, age-cohort, and cohort-period for both sexes(A), female(B), male(C)

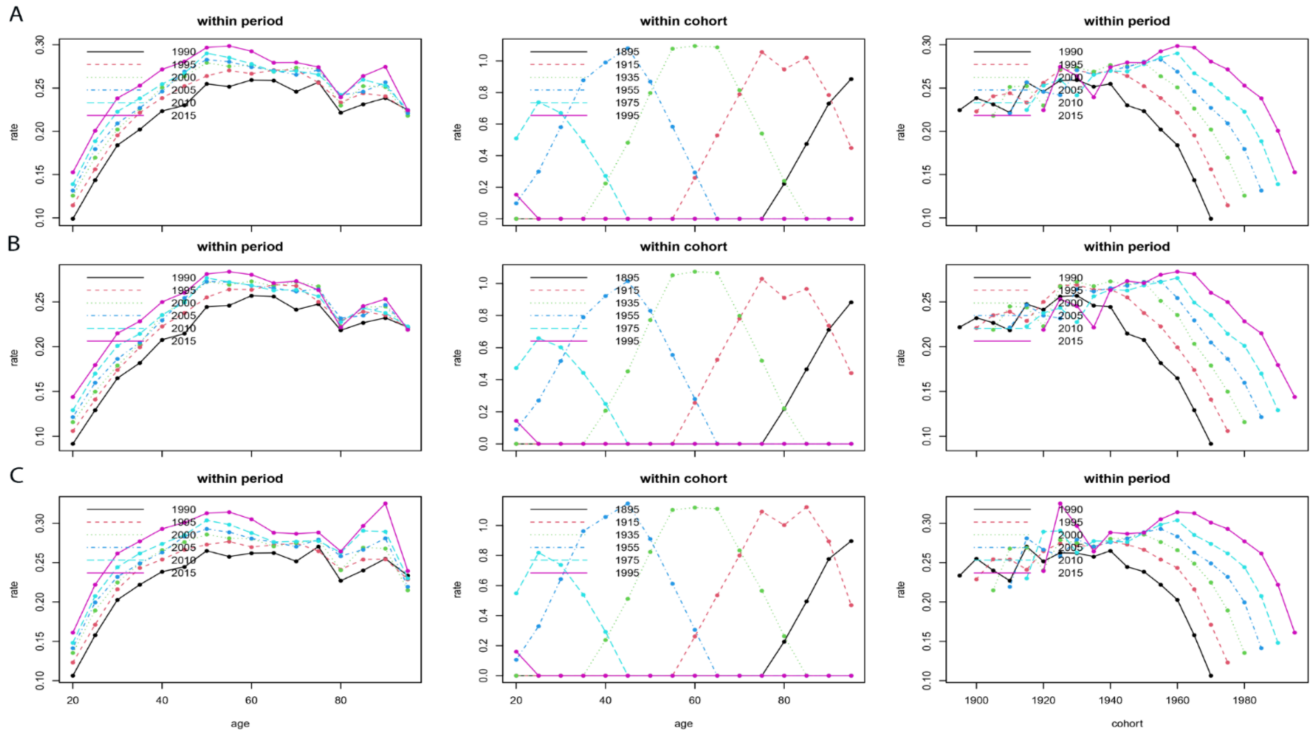

Figure 3. The trend of overweight prevalence rate within period-age, cohort-age, and period-cohort for both sexes(A), female(B), male(C)

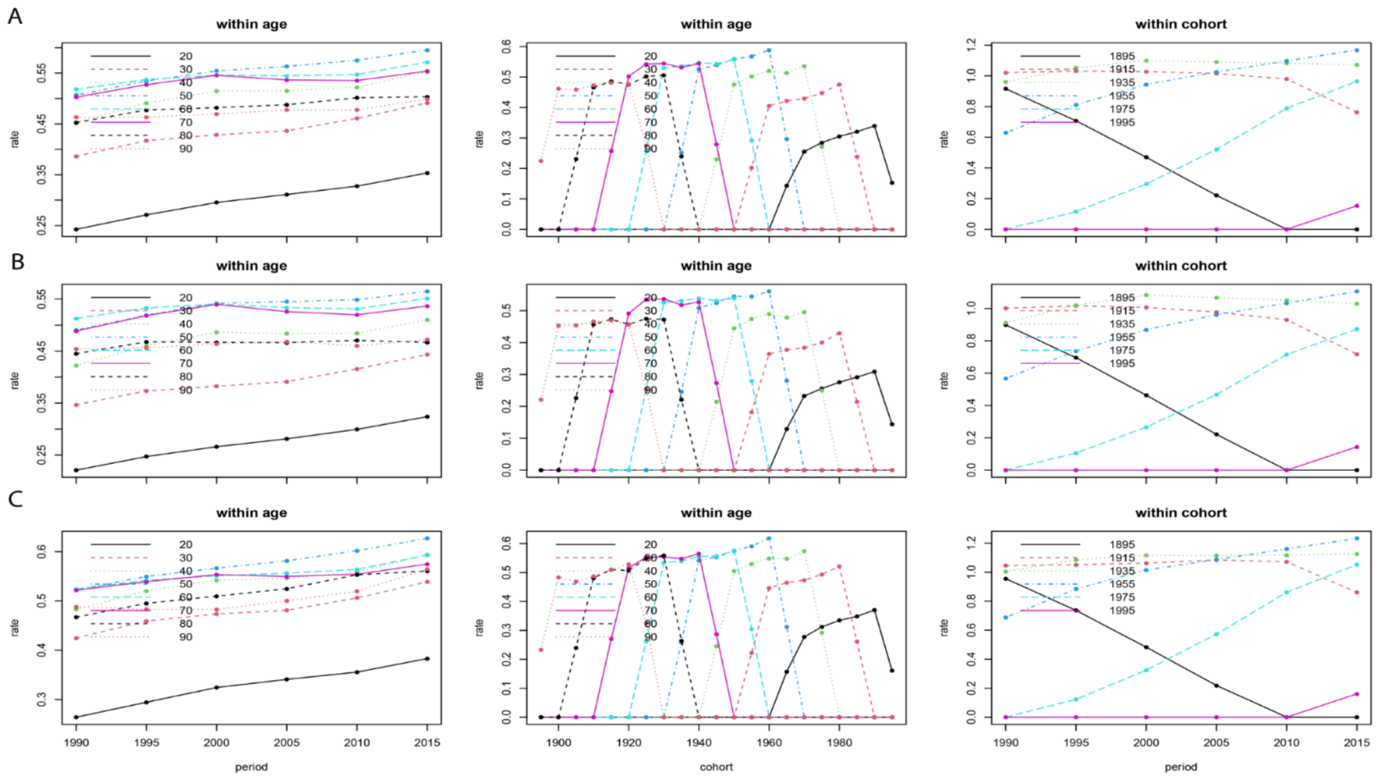

Figure 4. The trend of overweight prevalence rate within age-period, age-cohort, and cohort-period for both sexes(A), female(B), male(C)

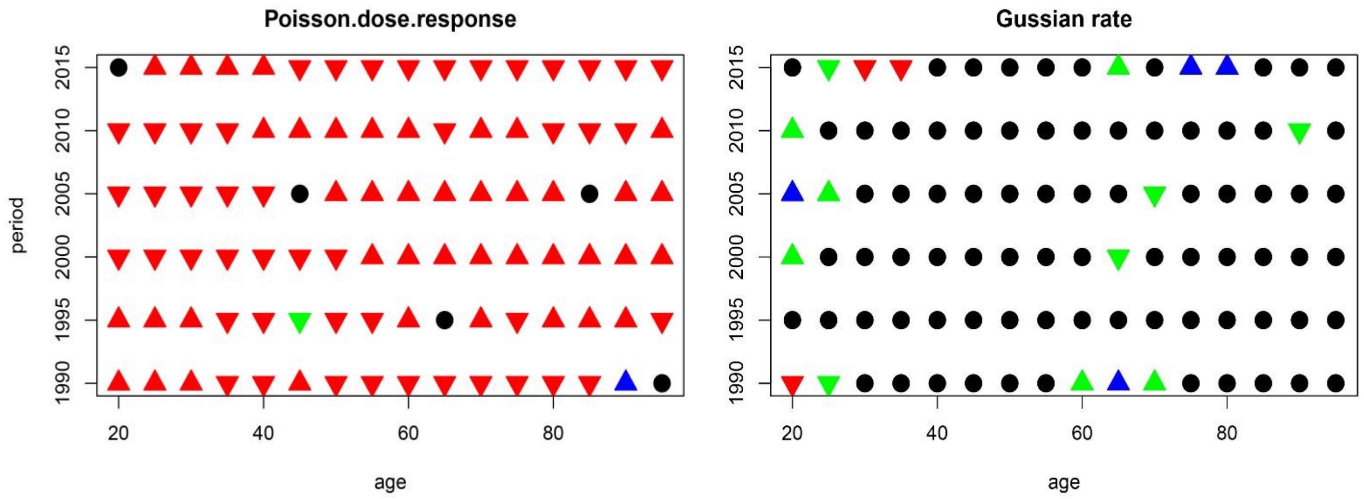

Figure 5. Probability transforms plots for the global obesity prevalence

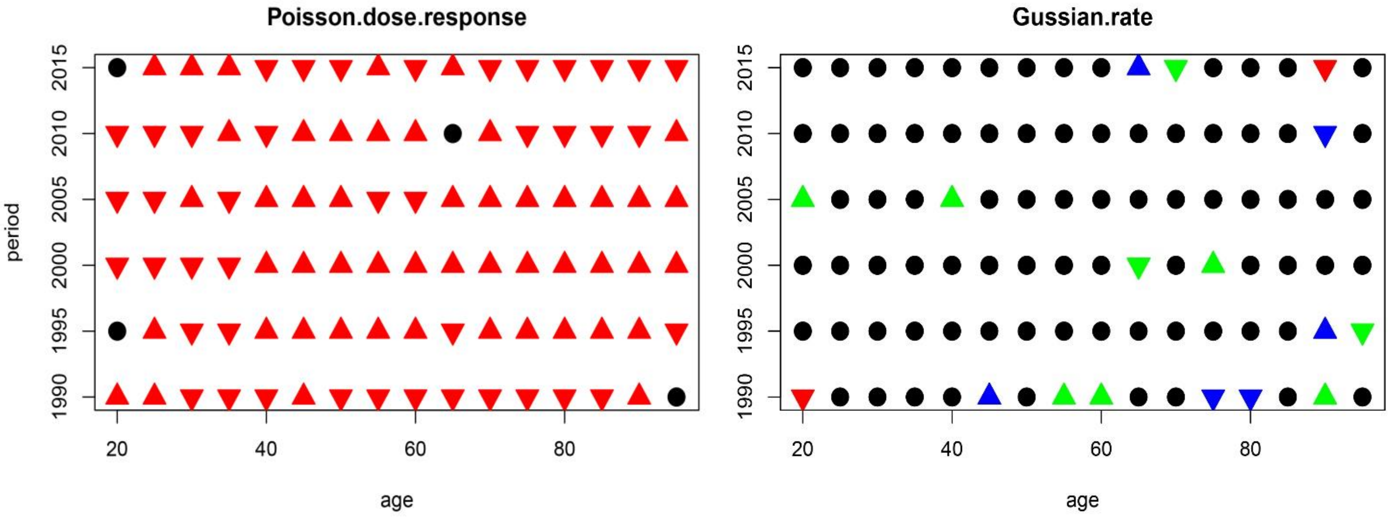

Figure 6. Probability transforms plots for the global overweight prevalence

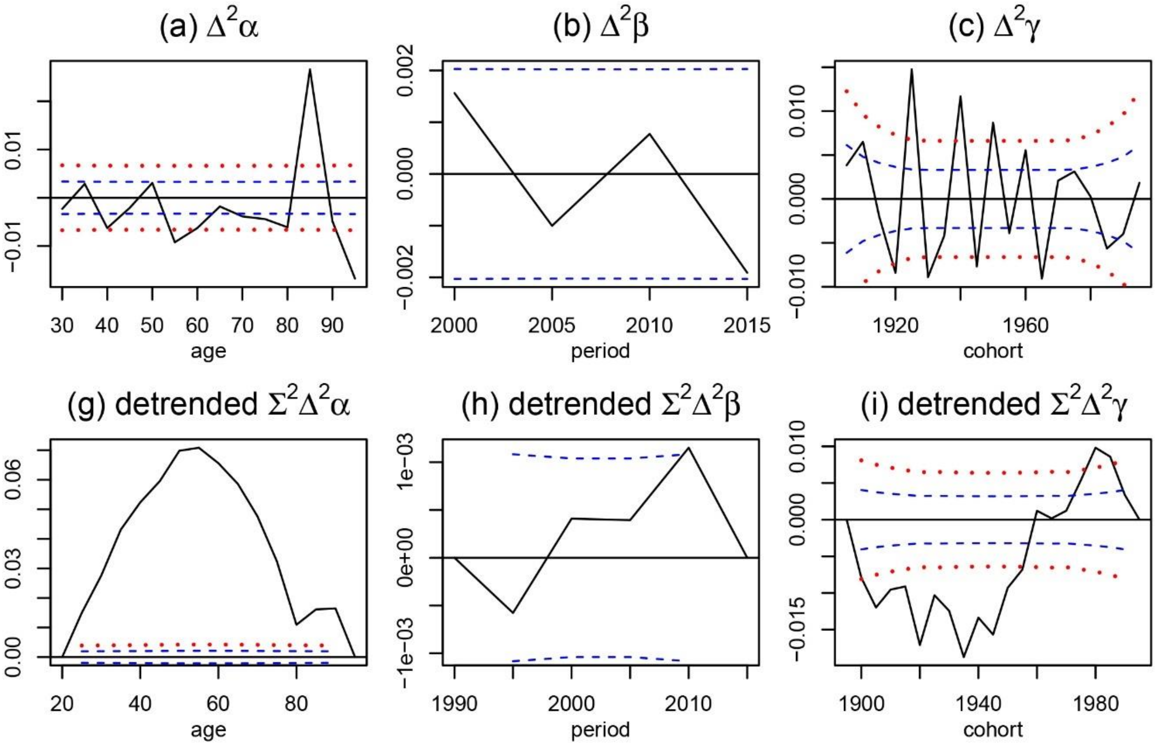

Figure 7. Plots of the results of the APC model fit on global obesity prevalence

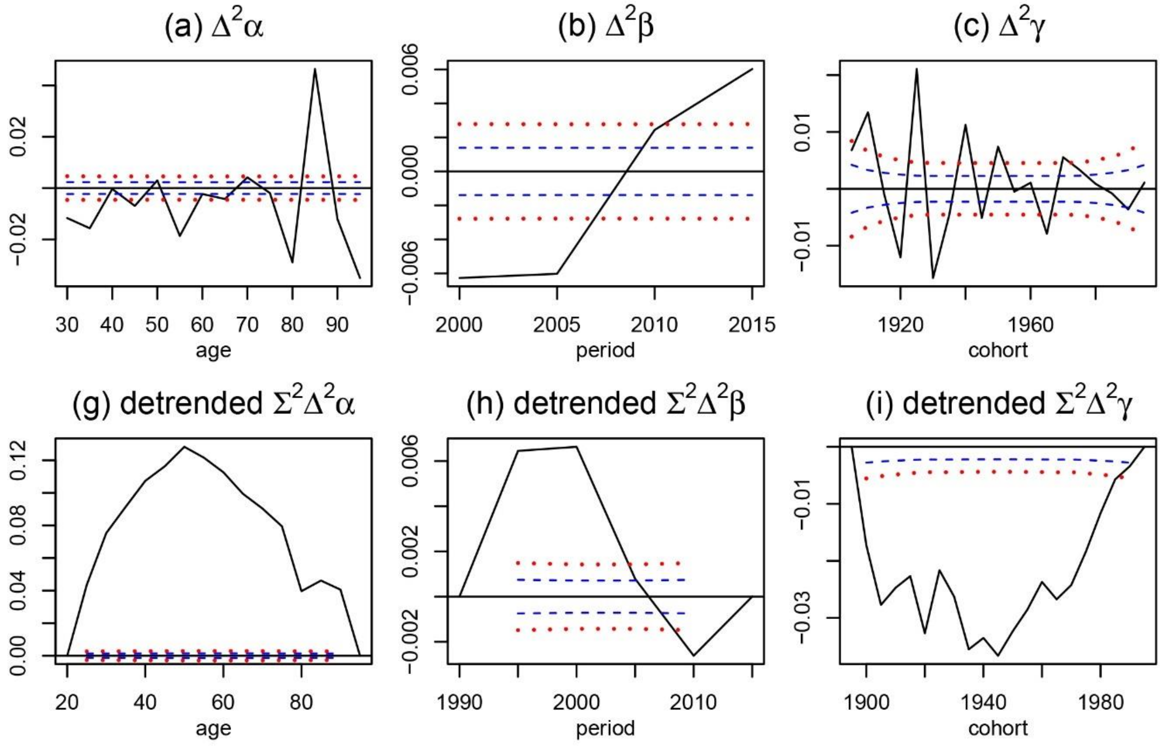

Figure 8. Plots of the results of the APC model fit on global overweight prevalence

Click here to access/download
Supporting Information
Figure1.docx

Click here to access/download
Supporting Information
TABLES SUPP.docx

Click here to access/download
Supporting Information
gather-checklist-apc.pdf
