# Supplementary material for: Prevalence patterns of overweight and obesity in the world: An age-period-cohort analysis
Source: PLoS One. 2025 Jun 18;20(6):e0324733. doi: 10.1371/journal.pone.0324733 (PMC12176128; doi:10.1371/journal.pone.0324733)
Supplement: Attachment — Submitted filename: Comments for 42238.docx [file pone.0324733.s003.docx]

**Reviewer’s comments for the Manuscript**

Thank you for submitting your manuscript, “Prevalence patterns of overweight and obesity in the world: An Age-Period-Cohort analysis,” to PLOS ONE. This review aims to provide constructive feedback to strengthen your research presentation. The study addresses a globally significant issue using a robust analytical framework, and your effort to employ the Age-Period-Cohort (APC) model is commendable. While the manuscript demonstrates strong potential, this critique highlights areas for improvement to enhance clarity, methodological transparency, and overall impact.

**Abstract**

1. There are grammatical issues, such as "inflation lost his pace," which detract from professionalism.
2. The phrase "modern lifestyle started to change in favor of become healthier" is unclear and could be refined for better readability.
3. Statistical findings, such as canonical coefficients, are mentioned but not quantified. Including specific metrics would strengthen the abstract.

**Introduction**

1. Over-reliance on previous reports without a critical evaluation of gaps they leave behind.
2. Certain statements, like "environmental factors such as economic growth," need clearer connections to obesity prevalence.
3. Some references (e.g., recent WHO data) might be outdated by the manuscript’s timeline, as newer reports are likely available.

**Methods**

1. The explanation of the identification problem in APC analysis is overly technical for a general research audience and could be simplified.
2. Potential limitations, such as biases in self-reported data or regional disparities, are acknowledged but not discussed in depth.
3. There is no mention of how missing data imputation might affect the robustness of results.

**Results**

1. While patterns are described, there is little interpretation of their public health implications.
2. Figures are referenced without providing explicit insights into anomalies, such as fluctuations in older age groups or gender-specific differences.
3. The increase in obesity prevalence in individuals over 80 years is highlighted but not explained adequately.

**Discussion**

1. The discussion is repetitive, reiterating results rather than exploring causative factors or implications.
2. Some claims, like the role of social media in reversing trends, lack direct evidence from the study and rely on secondary sources.
3. The potential impact of interventions is vaguely addressed, with no discussion of specific policies or strategies that have been successful.

**Conclusion**

1. The statement that modern lifestyles "started to change in favor of become healthier" is vague and unsupported by the data presented.
2. The conclusion could be strengthened by offering actionable recommendations or areas for future research.

**Strengths**

1. The study leverages a robust statistical model (APC) to dissect complex trends in obesity and overweight prevalence globally.
2. A large dataset from 195 countries ensures generalizability, and subgroup analyses add depth to findings.

**Limitations**

1. Data are outdated, which reduces the relevance of findings to current public health scenarios.
2. The study does not address heterogeneity across regions or income groups, which could mask significant disparities.
3. Missing an analysis of causal pathways or confounders influencing observed trends.

**Overall Recommendations**

1. Language and Grammar: Revise for grammatical errors and improve clarity throughout the manuscript.
2. Methodological Transparency: Expand on how data imputation and modeling decisions affect findings.
3. Contextual Depth: Include more nuanced discussions of regional disparities, socioeconomic factors, and policy implications.
4. Updated Data: If possible, integrate more recent data to improve the study's current relevance.
5. Figures and Tables: Enhance interpretation of visual data and ensure key trends are explicitly highlighted in the narrative.
